# Disability profiles in progressive multiple sclerosis reflect pathology distribution, independent of clinical phenotype

**DOI:** 10.1093/braincomms/fcag162

**Published:** 2026-05-02

**Authors:** Ermelinda De Meo, Amy E Jolly, Marco Ganzetti, Ferran Prados Carrasco, Baris Kanber, Jonathan Stutters, David McManus, Agne Kazlauskaite, Licinio Craveiro, Arman Eshaghi, James Cole, Declan Chard, Frederik Barkhof

**Affiliations:** NMR Research Unit, Department of Neuroinflammation, UCL Queen Square Institute of Neurology, Faculty of Brain Sciences, Queen Square MS Centre, University College London, London WC1B5EH, United Kingdom; NEUROFARBA, University of Florence, Florence 50100, Italy; NMR Research Unit, Department of Neuroinflammation, UCL Queen Square Institute of Neurology, Faculty of Brain Sciences, Queen Square MS Centre, University College London, London WC1B5EH, United Kingdom; Hoffman-La Roche Ltd., Roche-Pharma, Basel 4070, Switzerland; NMR Research Unit, Department of Neuroinflammation, UCL Queen Square Institute of Neurology, Faculty of Brain Sciences, Queen Square MS Centre, University College London, London WC1B5EH, United Kingdom; National Institute for Health Research (NIHR) University College London Hospitals (UCLH) Biomedical Research Centre, London NW1 2PG, United Kingdom; eHealth Center, Universitat Oberta de Catalunya, Barcelona 08018, Spain; NMR Research Unit, Department of Neuroinflammation, UCL Queen Square Institute of Neurology, Faculty of Brain Sciences, Queen Square MS Centre, University College London, London WC1B5EH, United Kingdom; NMR Research Unit, Department of Neuroinflammation, UCL Queen Square Institute of Neurology, Faculty of Brain Sciences, Queen Square MS Centre, University College London, London WC1B5EH, United Kingdom; NMR Research Unit, Department of Neuroinflammation, UCL Queen Square Institute of Neurology, Faculty of Brain Sciences, Queen Square MS Centre, University College London, London WC1B5EH, United Kingdom; Hoffman-La Roche Ltd., Roche-Pharma, Basel 4070, Switzerland; Hoffman-La Roche Ltd., Roche-Pharma, Basel 4070, Switzerland; NMR Research Unit, Department of Neuroinflammation, UCL Queen Square Institute of Neurology, Faculty of Brain Sciences, Queen Square MS Centre, University College London, London WC1B5EH, United Kingdom; Queen Square Institute of Neurology and UCL Hawkes Institute, University College London, London WC1V 6LJ, United Kingdom; NMR Research Unit, Department of Neuroinflammation, UCL Queen Square Institute of Neurology, Faculty of Brain Sciences, Queen Square MS Centre, University College London, London WC1B5EH, United Kingdom; National Institute for Health Research (NIHR) University College London Hospitals (UCLH) Biomedical Research Centre, London NW1 2PG, United Kingdom; NMR Research Unit, Department of Neuroinflammation, UCL Queen Square Institute of Neurology, Faculty of Brain Sciences, Queen Square MS Centre, University College London, London WC1B5EH, United Kingdom; Queen Square Institute of Neurology and UCL Hawkes Institute, University College London, London WC1V 6LJ, United Kingdom; Department of Radiology and Nuclear Medicine, Amsterdam UMC, Vrije Universiteit, Amsterdam, 22660, 1100 DD, Netherlands

**Keywords:** multiple sclerosis, progression, disability profiles, MRI, cognition

## Abstract

Multiple sclerosis (MS) is a clinically heterogeneous disease affecting both neurological and cognitive functions. In this study, we defined clinical profiles based on combined cognitive and motor assessments in progressive MS patients, examined their overlap with classical progressive phenotypes and linked each profile to MRI measures of network disconnection and grey matter atrophy. We analyzed baseline data from 580 participants [277 primary progressive (PP)MS, 303 secondary progressive (SP)MS] in the phase 3b CONSONANCE (NCT03523858) study. Clinical assessments included Expanded Disability Status Scale, 9-Hole Peg Test, timed 25-Foot Walk Test, Symbol Digit Modalities Test and Brief Visuospatial Memory Test Revised. MRI acquisition comprised Fluid Attenuated Inversion Recovery for lesion segmentation and 3D-T1 weighted images for disconnectome reconstruction and volumetry. Independent component analysis was applied to regional disconnection and volumes to, respectively, identify patterns (ICs) of disconnection and grey matter atrophy. Latent profile analysis (LPA) identified disability profiles from multimodal clinical data. To identify the variables most strongly associated with each LPA-defined profile, we trained one-versus-all eXtreme Gradient Boosting classifiers and computed SHapley Additive exPlanations values. Based on prevailing clinical manifestation, LPA delineated three profiles: motor disability (*n* = 138, 23.8%), cognitive disability (*n* = 181, 31.2%) and global disability (*n* = 261, 45.0%). Profile prevalence did not differ by clinical phenotype (χ^2^, *P* = 0.675). Compared with the other profiles, motor disability one was associated with relatively preserved connectivity and tissue volume in the limbic and frontal networks and preserved volumes in the supplementary motor cortex and postcentral gyrus. The cognitive disability profile had relatively lower connectivity and volumes in the cerebellar vermis, temporal pole, posterior cingulate gyrus parietal operculum, lower default-mode-network connectivity and prefrontal–visual integration network volumes. The global disability profile had lower fronto-parietal-network connectivity and lower grey matter, superior frontal gyrus, pallidum, cuneus and cerebellum volumes. Our findings reveal that progressive MS encompasses at least three clinically and biologically distinct subtypes transcending the traditional PPMS/SPMS divide. The cognitive disability profile is driven by focal network disconnection and targeted grey matter loss in critical cognitive hubs, whereas the global disability profile reflects widespread grey matter atrophy across motor and associative regions. Identifying these profiles could be useful in clinical trials by matching outcome measures to underlying patterns of pathology and their clinical manifestations.

## Teaser

De Meo *et al*. analyzed data from 580 progressive multiple sclerosis patients using clinical tests and MRI-derived disconnectome and volumetry. Latent profile analysis identified three subtypes—motor, cognitive and global disability—each with distinct signatures, characterized by differential contribution of network disconnection and grey matter atrophy transcending traditional primary and secondary progressive multiple sclerosis classifications.

## Graphical Abstract

**Graphical Abstract:**
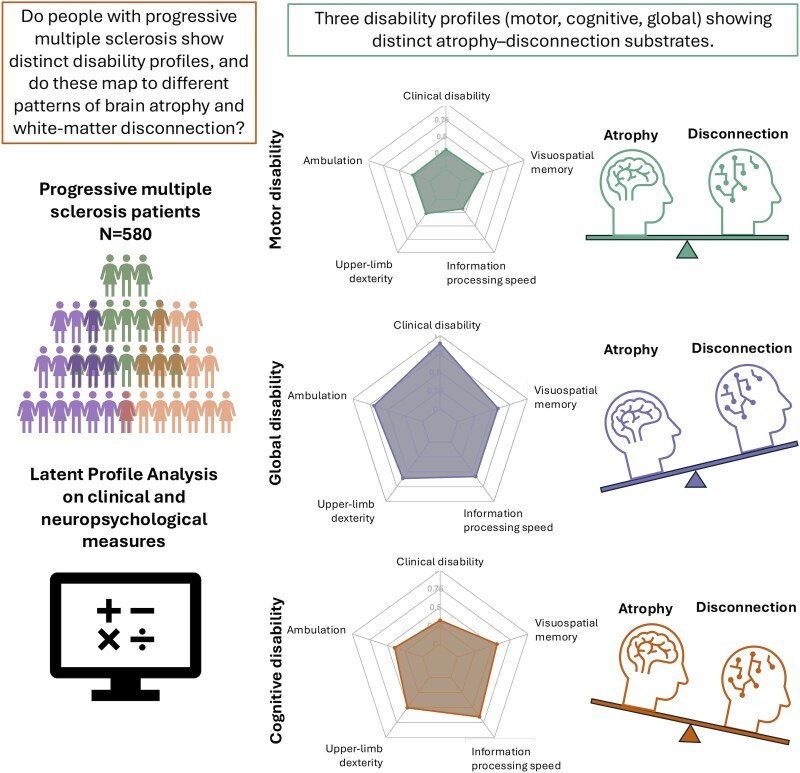

## Introduction

Multiple sclerosis (MS) is heterogenous in the pattern and severity of functions it affects. However, patients are typically categorized according to broad phenotypes that reflect the clinical presentation at the disease onset or during transition phases, i.e. relapsing-remitting, primary progressive (PP) and secondary progressive (SP) MS. Additionally, severity is often defined using the Expanded Disability Status Scale (EDSS), which is heavily weighted towards ambulation deficits.^1,2^ As a result, progressive deficits that are functionally relevant may be missed, in particular if it is assumed that people with MS evolve in a monotonic fashion according to clinical descriptors. In fact, distinct neurological and cognitive deficit profiles may be observed even in people with the same MS clinical phenotype and similar EDSS scores. Latent profile analysis (LPA) has identified subgroups with divergent patterns of cognitive deficits that are independent of clinical phenotype, suggesting that MS clinical heterogeneity is not random and that conventional phenotypic categories do not mirror this clinical diversity.^3^ LPA considers multiple dimensions simultaneously in a so-called latent space and identifies which dimensions are more commonly observed across certain patients, allowing them to cluster them according to the probability of belonging to a specific group.^4-7^ To date, LPA studies have not focused specifically on progressive MS, yet it is during the progressive phase of disease that people accrue most of their impairment.^8^

Given this, clinical (and MRI) separation between LPA subgroups should be clearer in progressive cohorts compared with those including people with earlier and more focally inflammatory disease, and so otherwise overlooked subtypes may be observed. Moreover, it has already been shown that disease-modifying treatment for progressive MS shows greater effects on disability progression in specific subgroups of patients (based on the presence of ongoing focal inflammatory activity rather than deeper phenotyping).^9,10^

As with neurological and cognitive deficits, the distribution and magnitude of MS pathology in the brain, assessed using MRI, are also highly heterogeneous. Yet, MRI measures that are typically assessed at a whole-brain level usually explain less than half of the variance in clinical outcomes.^11^ Considering that cognitive, motor or sensory functions rely on specific networks,^12-14^ there has been an evolution towards network-based MRI analyses. White matter approaches have typically assessed how lesions intersect with tracts and derived whole-brain ‘disconnectomes’ that reflect overall network disruption, rather than matching patterns of disconnection to clinical outcomes.^15^ In grey matter, atrophy does not homogeneously affect the whole brain,^16,17^ but preferentially involves regions associated with specific neural networks (e.g. motor).^18,19^ The application of independent component analysis (ICA) to structural MRI has identified covarying regional grey matter atrophy (spanning multiple cortical and deep grey matter structures) that is associated with clinical disability.^20^ White and grey matter network-based metrics appear to outperform whole-brain measures in explaining clinical outcomes, but the possibility that differential effects on networks may explain which clinical domains are most affected in people with MS has not been explored^6,19,21^ We hypothesize that clinical subgroups identified using LPA may reflect differential patterns of damage to neural networks.

With a view to better understanding and explaining clinical heterogeneity, we studied a large cohort of people with PPMS and SPMS and tested three hypotheses: (i) despite similar EDSS scores, clinical subgroups can be identified based on patterns of cognitive and neurological function; (ii) LPA-derived clinical subgroups span across conventional MS phenotypes and (iii) the distribution of pathology in terms of disconnection and grey matter atrophy differs between LPA-derived subgroups. We did so by applying LPA to cognitive and neurological measures to identify homogenous subgroups across cognitive and neurological impairment. We then tested how these profiles mapped onto PPMS versus SPMS and compared MRI markers at three levels of analysis: (i) whole-brain metrics (total lesion volume, global disconnectome burden, normalized brain, grey and white matter volumes); (ii) region-specific measures (disconnection and volume for individual ROIs such as thalamus, basal ganglia, brainstem and cortical regions) and (iii) network patterns identified via ICA (components reflecting covarying atrophy and disconnection across interconnected clusters).

## Materials and methods

### Participants

We analyzed baseline data from 580 people with progressive MS enrolled in the CONSONANCE study (NCT03523858), an ongoing prospective, multicentre, single-arm, phase 3b study evaluating the effectiveness and safety of ocrelizumab in progressive MS. Only participants with complete clinical and MRI data at the time of analysis were included.

### Clinical and cognitive assessment

All participants underwent clinical and functional assessments including EDSS, 9-Hole Peg Test (9HPT) [mean of left and right hand], timed 25-Foot Walking Test (25FWT) and cognitive assessments including oral Symbol Digit Modalities Test (SDMT) and the Brief Visuospatial Memory Test Revised (BVMT-R).

### MRI acquisition

The following MRI sequences were obtained: 2D T2-weighted (T2-w) Fast Spin Echo (FSE), 2D Proton Density weighted (PD-w), 2D Fluid Attenuated Inversion Recovery (FLAIR) and 3D T1-weighted (T1-w). Further details, including the complete MRI protocol and acquisition parameters for the different scanners, are reported in Supplementary Table 1. In this study, we used 2D T2-w FSE, 2D PD-w and 2D FLAIR for lesion identification and segmentation, and 3D T1-w for brain tissue and grey matter segmentation.

### Latent profile analysis

LPA is a person-centred clustering technique that can identify subgroups based on multiple features. LPA is based on mixture models^4^ that analyze the joint distribution of a set of continuous observed variables as a function of a finite and mutually exclusive unobserved components using a latent categorical variable (or *profile*).^22,23^ LPA does not require any *a priori* categorization of the observed variables and so enables a more granular examination of heterogeneity within and between latent-level groupings when compared with traditional clustering methods.^4,24^ We performed LPA^4,25^ using scores from EDSS, 9HPT, 25FWT, SDMT and BVMT-R. Despite its recognized limitations, the EDSS was retained because it remains the standard clinical measure in treatment trials and clinical practice and adds complementary information on non-cognitive functional systems (e.g. brainstem, sensory, cerebellar and sphincteric domains) not captured by the other measures. To standardize all scores on a common scale, we normalized each measure individually using the empirical cumulative distribution function (ECDF).^26^ The ECDF assigns a value between 0 and 1 to each score based on its percentile rank within the distribution of that specific measure. This resulted in a severity scale for each measure, where a score of 0 corresponds to the best performance (indicating the least impairment) and a score of 1 represents the worst performance (indicating the most severe impairment). By normalizing one measure at a time, we ensured that all measures were directly comparable on a consistent scale. An important step of LPA is the selection of the best-fitting model, usually a parsimonious model aiming at identifying stable profiles or classes. Models with up to 10 profiles were run, and Bayesian Information Criterion (BIC) and Akaike Information Criterion (AIC) were used to determine the optimal number of profiles, in line with Nylund *et al*.^27^ and Scrucca *et al*.^28^ We then performed 10-fold cross-validation (CV) to assess generalisability and stability. Both criteria mentioned above (AIC and BIC) are widely employed in model selection, balancing goodness-of-fit with model complexity. Lower absolute BIC and AIC values indicate better model fit, with a trade-off between improving fit and avoiding overfitting. A systematic evaluation of these metrics across models with varying numbers of profiles was performed, and the optimal model was selected based on the point at which additional profiles provided diminishing returns in terms of fit improvement. Posterior probabilities (ranging from 0 to 1) represent the likelihood of an individual belonging to each latent profile, given their observed data and the parameters of the selected model. Participants were classified into the profile for which they had the highest posterior probability. For this analysis, we used tidyLPA R package (https://data-edu.github.io/tidyLPA/).^29^

### Image pre-processing

All MRI scans underwent visual quality controls for motion, slice drop-out and other imaging artefacts. Only scans that passed were retained for analysis. As part of the lesion segmentation pipeline,^30^ the 3D T1-w images were aligned to a common space using a two-step procedure. First, a 9 degrees-of-freedom affine registration—comprising three rotations, three translations and three scaling steps—was performed to align each image to the MNI152 template (ICBM 2009c Nonlinear Symmetric; https://nist.mni.mcgill.ca/icbm-152-nonlinear-atlases-2009/). From this registration, a six-parameter rigid-body transformation (rotation and translation only) was extracted and applied to the image. This approach, referred to as stereotactic orientation, ensures that no scaling is applied, thereby preserving native anatomical dimensions. All additional MRI sequences were rigidly registered to the corresponding 3D T1-w image, and the concatenated transformations were used to resample them into the same stereotactic space.^31^ Lesions were identified and segmented using a two-stage classification approach. First, a voxel-wise Bayesian probabilistic classification of healthy tissue and lesions was performed, with prior probabilities derived from a multi-atlas label fusion approach.^30^ The intensity likelihoods were based on 2D T2-w, 3D T1-w, 2D PD-w and 2D FLAIR intensities. Second, a lesion-level random forest classification was applied to the voxels labelled as potentially lesional in the first step.^32^ Lesions were then reviewed and, when required, edited by two experienced observers (EDM and AJ).

Lesion-filled^33^ 3D T1-w images were linearly interpolated to 1 mm isotropic resolution. Subsequently, geodesic information flow (GIF) was used to compute the total intracranial volume, and segment brain tissue (grey matter, white matter) and cerebrospinal fluid.^34^ To provide regional segmentations of 123 cortical and deep grey matter regions, the Neuromorphometrics Desikan-Killiany-Tourville (DKT) atlas was used. Global and regional grey matter volumes were normalized by the total intracranial volume.

For disconnectome analyses, lesion-filled 3D T1-w images were registered to the ICBM152 2009a space (corresponding to the space of the healthy control tractogram we used, see next section for details).^35,36^ This was done in two steps: first, each lesion-filled 3D T1-w was linearly aligned to the template using an affine transformation (12 degrees of freedom, including translations, rotations, scaling and shearing) to correct for global differences in head position and brain size. Second, a high-dimensional non-linear registration was applied to the same template to accommodate local anatomical variability and atrophy. The resulting affine and non-linear deformation fields were then applied to the corresponding lesion masks using nearest-neighbour interpolation to preserve lesion boundaries. All registrations were visually inspected by two observers (AJ and EdM), to ensure they were adequate (See Supplementary Video 1).

### Network disconnection quantification

Disconnectomes were generated at the whole-brain level, where a disconnectome represents a map of brain regions likely to be structurally disconnected (including partially) due to lesions in the connecting tracts.^37^ To quantify whole brain and regional disconnection, we implemented a pipeline described by Ravano *et al*. (https://gitlab.com/acit-lausanne/lesion-disconnectomics), 15 using a healthy control tractogram from the human connectome project (HCP 1065). For further details about the construction of the template please refer to the HCP 1065 website (https://brain.labsolver.org/hcp_template.html) and the associated publications.^38,39^ A custom template was built using software package *DSI Studio* (https://dsi-studio.labsolver.org) and all resulting disconnectome analyses utilized the same software. Individual lesion masks were registered to the HCP 1065 template space and intersected with the healthy tractogram using *DSI Studio* to identify white matter streamlines that pass through the lesions. Using the DKT grey matter atlas to define regions of interest (ROIs), a connectome was constructed to represent lesion-affected connectivity or affected connectome (AC), quantified as the total number of streamlines passing through lesions and connecting specific grey matter regions. This connectome is represented as an *N* × *N* adjacency matrix, where *N* is the number of ROIs defined by the DKT atlas. Each element M_{ij} in the matrix indicates the total number of streamlines passing through lesions and connecting ROI i to ROI j, thereby quantifying the extent of connectivity disruption between all pairs of grey matter regions. To quantify disconnection as the proportion (%) of streamlines affected by lesions, the following calculation was used:


$$\mathrm{Disconnectome}\;=\;1\;\text{-}\frac{\left(HC-AC\right)}{\;HC}$$

where AC represents the affected connectome of the individual with MS and HC represents the healthy connectome derived from the HCP 1065 tractogram. Values of disconnection can range from 0 (representing preserved connectivity) to 1 (representing complete disconnection).

### Patterns of disconnection and grey matter damage

To identify patterns of grey matter damage and disconnection, we separately run ICA on grey matter volume (obtained in native space using GIF segmentation of lesion-filled 3D T1-w images and normalized by the total intracranial volume) and regional disconnection data (proportion of streamlines from/to each region intersecting the lesion mask). ICA was performed using the FastICA^40^ implementation in R (https://github.com/cran/fastICA/blob/master/R/fastICA.R), which applies a fixed-point algorithm to estimate the unmixing matrix that maximizes non-Gaussian component signals. The non-Gaussian contrast function was set to logcosh, as recommended for robustness, and components were extracted in the symmetric mode to estimate all sources simultaneously. Convergence was assessed using a fixed tolerance (tol = 1 × 10^−4^) and a maximum of 200 iterations; at convergence, the estimated components are decorrelated and statistically independent in the ICA sense. We run FastICA^40^ with 20 components, a commonly used number in neuroimaging studies, which provides a balance between capturing meaningful signals and avoiding overfitting.^41^ Considering the number of regions (*n* = 123) with lower dimensionalities (e.g. 10–15 components), anatomically and functionally distinct systems tend to be merged into broad, non-specific patterns (e.g. sensorimotor, visual and associative regions loading on the same component) while higher model orders (≥25–30 components) produce fragmented maps with small and spatially scattered clusters. The output of ICA provided independent components reflecting spatial patterns of grey matter volumes (when using regional grey matter volume as input) or disconnection (when using regional disconnection as input).

### Statistical analysis

Comparisons of demographic and clinical parameters between PPMS and SPMS and among the newly defined disability profiles were performed using linear regression and mixed-effects models, chi-square test or non-parametric tests, as appropriate (normal distribution was assessed by visual inspection and Kolmogorov–Smirnov test). Age, sex, disease duration (defined as the time between symptom onset and the date of enrolment in the study) and scanner model (for MRI variables) were included as covariates when comparing clinical, neuropsychological and MRI measures between PPMS and SPMS, as well as across the newly defined disability profiles.

To test the effect of clinical phenotype among participants with equivalent EDSS and duration, we performed 1:1 nearest-neighbour propensity score matching (without replacement) using propensity scores from a logistic regression with EDSS and disease duration as covariates, applying a 0.2-standard deviation calliper on the pooled propensity-score distribution.

To identify the variables most strongly associated with each LPA-defined profile, we first trained a one-versus-all classification model for each profile using eXtreme Gradient Boosting (XGBoost), an ensemble-based gradient boosting algorithm. The XGBoost classifiers were trained in a one-versus-all scheme using stratified 10-fold outer CV. Within each outer training fold, hyperparameters were tuned by stratified 5-fold inner CV with early stopping and built-in regularization, selecting the configuration with the highest mean validation area under the curve (AUC). The selected model was then refitted on the full outer-training fold and used to predict the held-out fold, yielding out-of-fold predictions for every participant. To avoid interpretative leakage, we computed SHapley Additive exPlanations (SHAP) values using the TreeSHAP algorithm on the held-out fold data with the corresponding fold-specific models and aggregated these across folds. In this framework, each classifier estimates the probability of membership in a given profile versus the remainder of the cohort. SHAP values provide a transparent and interpretable measure of feature importance by quantifying the contribution of each variable to the model’s predictions.^42^ Unlike traditional feature importance metrics, SHAP values account for feature interactions and collinearity, making them particularly suitable for identifying biologically meaningful variables rather than merely optimizing predictive accuracy.^43,44^

Feature relevance was visualized through Beeswarm plots,^45^ capturing both the magnitude and direction of each feature’s influence. Features were selected based on their mean absolute SHAP values, retaining only those contributing most significantly to phenotype classification. This method allows for a robust and interpretable identification of key biomarkers underlying clinical disability, facilitating a more nuanced understanding of MS heterogeneity and potential personalized approaches to disease monitoring).

As a sensitivity analyses, (i) we re-run LPA separately in PP and SPMS (See Supplementary Fig 6 and Supplementary Tables 7–10) and (ii) we applied Least Absolute Shrinkage and Selection Operator (LASSO) regression to select the most relevant feature for each LPA-derived profile (See Supplementary Fig. 7). Statistical significance was corrected for multiple comparisons (False Discovery Rate),^46^ and the threshold for significance was set at corrected *P* < 0.05. Statistical analysis was performed using R 4.2.2.

## Results

### Clinical, demographic and conventional MRI features of the study cohort

Of the 580 participants, 277 had PPMS and 303 SPMS. Demographic, clinical and conventional MRI measures are summarized in Table 1.

**Table 1:** Summarizes demographic, clinical, neuropsychological and conventional MRI variables of patients included in the study

	All (*n* = 580)	PPMS (*n* = 277)	SPMS (*n* = 303)	FDR-corrected *P* values
Mean age (SD)	48.4 (9.2)	49.5 (8.8)	47.5 (9.5)	0.008
Gender (F:M)	310:270	134:143	176:127	0.02
Disease duration from symptom onset (years)	11.6 (8.0)	7.2 (4.3)	15.7 (8.4)	<0.001
EDSS, median (IQR)	5.5 (4.0–6.0)	4.5 (4.0–6.0)	6.0 (4.5–6.0)	<0.001
25FWT, mean (SD), seconds	14.9 (17.2)	12.7 (14.3)	17.0 (19.3)	0.003
9HPT, mean (SD), seconds	32.5 (15.5)	30.7 (12.4)	34.1 (17.7)	0.009
SDMT, mean (SD)	42.6 (13.7)	43.0 (13.8)	42.1 (13.6)	0.45
BVMT-R, mean (SD)	18.8 (8.1)	19.0 (8.1)	18.7 (8.1)	0.68
T2 lesion volume (SD), ml	17.425 (15.515)	16.147 (15.822)	18.593 (15.160)	0.007
Normalized a brain volume, mean (SD)	0.808 (0.020)	0.810 (0.020)	0.806 (0.021)	0.004
Normalized a grey matter volume, mean (SD)	0.439 (0.012)	0.440 (0.012)	0.438 (0.013)	0.10
Normalized a white matter volume, mean (SD)	0.369 (0.014)	0.370 (0.014)	0.367 (0.014)	0.007
Total intracranial volume, mean (SD), ml	1461.057 (146.945)	1481.297 (151.443)	1442.558 (140.429)	0.001

Abbreviations: FDR = false discovery rate; F = female; M = male; PPMS = primary progressive MS; SD = standard deviation; SPMS = secondary progressive MS.

^a^Normalized volumes are obtained by dividing individual volumes by the total intracranial volume.

### Three disability profiles are seen

**Figure 1:**
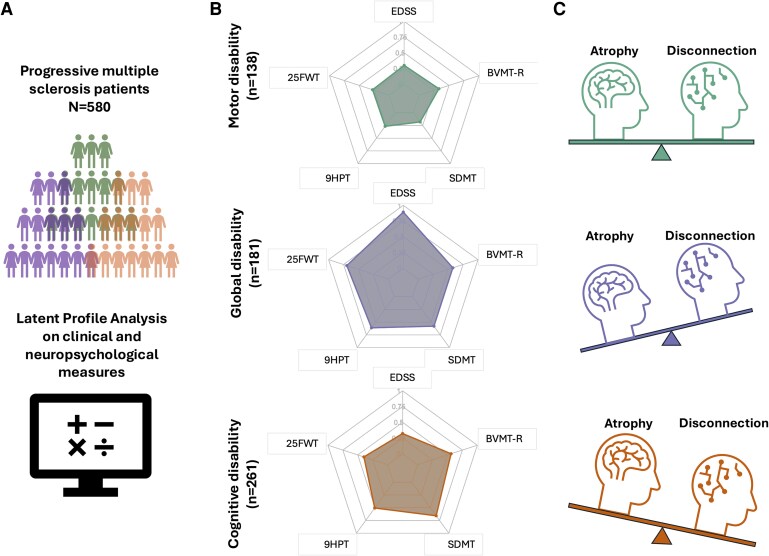
**Data-driven disability profiling reveals distinct atrophy–disconnection substrates in progressive multiple sclerosis.** Progressive multiple sclerosis patients (*n* = 580) were grouped using latent profile analysis (A) of clinical disability, motor function and neuropsychological test scores into three disability profiles (B)—motor (*n* = 138), global (*n* = 181), and cognitive (*n* = 261)—defined by the domain with the greatest impairment. (C) summarizes the relative contributions of brain atrophy and white matter disconnection for each profile. Abbreviations: EDSS = Expanded Disability Status Scale, 9HPT = Nine-Hole Peg Test, 25FWT = timed 25-Foot Walking Test, SDMT = Symbol Digit Modalities Test, BVMT-R = Brief Visuospatial Memory Test-Revised.

**Figure 2:**
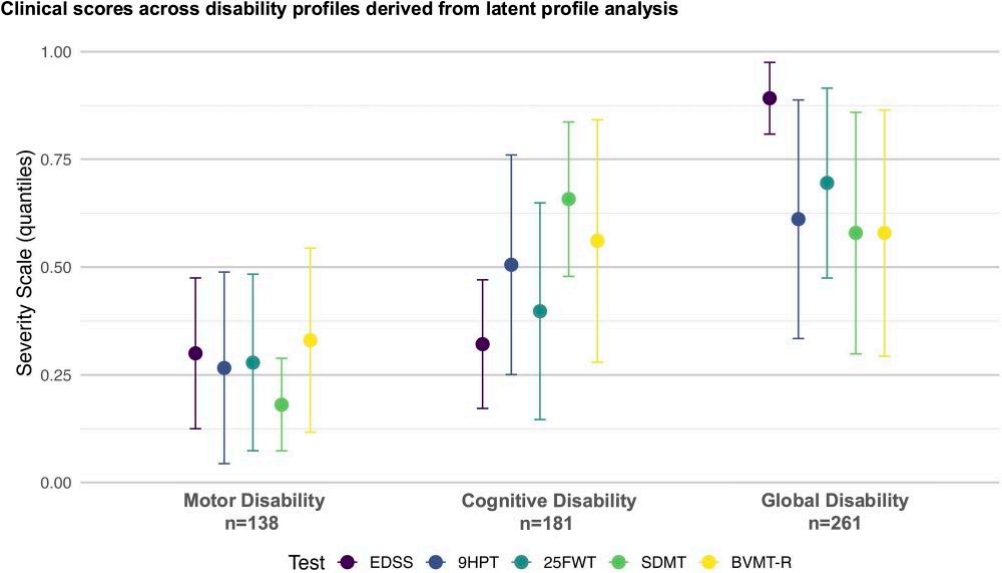
**Clinical scores across disability profiles derived from latent profile analysis.** Summarizes the means and standard deviations of the normalized test scores—derived via the empirical cumulative distribution function—for each assessment across the three disability profiles. Abbreviations: EDSS = Expanded Disability Status Scale, 9HPT = Nine-Hole Peg Test, 25FWT = timed 25-Foot Walking Test, SDMT = Symbol Digit Modalities Test, BVMT-R = Brief Visuospatial Memory Test-Revised.

Models with 1 to 10 profiles were run. Based on the lowest absolute (best) BIC and AIC scores (Supplementary Table 2 reports BIC and AIC for all models tested), the model with three profiles was identified as having the best fit. Based on the severity of the impairment in the clinical and neuropsychological measures used for LPA, the three profiles were designated as follows: (i) ‘Motor disability’ characterized by motor disability and preserved cognitive functions (*n* = 138, 23.8%); (ii) ‘Cognitive Disability’ characterized by severe cognitive impairment and moderate physical disability (*n* = 181, 31.2%) and (iii) ‘Global disability’ characterized by severe global disability and moderate cognitive impairment (*n* = 261, 45.0%). The mean (SD) posterior probability of membership was 0.89 (0.12), 0.99 (0.01) and 0.96 (0.09), respectively, indicating a high likelihood of individuals belonging to each latent profile. Figure 1 provides a graphical representation of study findings, and Fig. 2 summarizes the severity of impairment across the various tests for the different disability profiles.

### Disability profiles are not explained by demographic features or MS phenotype

Table 2 and Supplementary Fig. 1 present the between-group comparisons among the disability profiles. No significant differences in age or sex were observed across the identified disability profiles. The global disability profile was associated with longer disease duration, and more frequently had SPMS, compared to the other two LPA subgroups. To test the effect of clinical phenotype among participants with similar disability and disease duration, we identified groups of PPMS (*n* = 157) and SPMS (*n* = 157) propensity-matched for EDSS and disease duration (more details are available in Supplementary Fig. 2). Within these matched groups, the distribution of clinical phenotypes across the newly identified disability profiles did not differ: motor disability (PPMS/SPMS: 37/38), cognitive disability (PPMS/SPMS: 50/43) and global disability (PPMS/SPMS: 70/76), (chi-square test) FDR-corrected *P*-value = 0.675.

**Table 2:** Summarizes demographic, clinical and conventional MRI features among the newly defined disability profiles

	Profiles			Comparison, FDR-corrected *P*-value		
	Profile 1 Motor disability (*n* = 138)	Profile 2 Cognitive disability (*n* = 181)	Profile 3 Global disability (*n* = 261)	Profile 1 versus 2	Profile 1 versus 3	Profile 2 versus 3
Mean age (SD), years	47.9 (8.8)	47.8 (9.3)	49.2 (9.3)	0.945^a^	0.401^a^	0.182^a^
Sex (F:M), *n*	71:67	93:88	146:115	1.00^b^	1.00^b^	1.00^b^
Mean disease duration (SD), years	10.1 (7.1)	10.0 (8.0)	13.5 (8.0)	0.997^a^	**<0.001** ^a^	**<0.001** ^a^
Clinical phenotype (PPMS:SPMS), *n*	75:63	105:76	97:164	1.000^b^	**<0.001** ^b^	**<0.001** ^b^
Mean lesion volume (SD), ml	10.799 (14.661)	21.628 (14.780)	18.013 (15.349)	**<0.001** ^c^	**<0.001** ^c^	**0**.**032**^c^
Normalized brain volume, mean (SD)^e^	0.817 (0.018)	0.807 (0.019)	0.803 (0.020)	**<0.001** ^c^	**<0.001** ^c^	0.124^c^
Normalized grey matter volume, mean (SD)^e^	0.444 (0.011)	0.440 (0.012)	0.436 (0.013)	**0**.**007**^c^	**<0.001** ^c^	**0**.**029**^c^
Normalized white matter volume, mean (SD)^e^	0.374 (0.013)	0.368 (0.014)	0.367 (0.014)	**<0.001** ^c^	**<0.001** ^c^	0.828^c^
Total intracranial volume, mean (SD), ml	1486.184 (130.708)	1465.147 (150.622)	1444.935 (150.896)	0.418^c^	**0.023** ^c^	**0**.**340**^c^
EDSS, median (IQR)	4.0 (3.0–5.0)	4.5 (3.5–5.0)	6.0 (6.0–6.5)	0.181^d^	**<0.001** ^d^	**<0.001** ^d^
25FWT, mean (SD), seconds	7.2 (2.8)	9.5 (5.5)	22.8 (22.9)	0.393^d^	**<0.001** ^d^	**<0.001** ^d^
9HPT, mean (SD), seconds	24.3 (5.7)	30.5 (9.2)	38.3 (19.7)	**<0.001** ^d^	**<0.001** ^d^	**<0.001** ^d^
SDMT, mean (SD)	56.5 (6.1)	36.8 (9.3)	39.2 (14.1)	**<0.001** ^d^	**<0.001** ^d^	**0**.**033**^d^
BVMT-R, mean (SD)	23.9 (5.8)	17.6 (7.8)	17.0 (8.1)	**<0.001** ^d^	**<0.001** ^d^	0.754^d^

Abbreviations: FDR = false discovery rate; F = female; M = male; PPMS = primary progressive MS; SD = standard deviation; SPMS = secondary progressive MS.

Bold type indicates statistical significance.

^a^Linear regression models.

^b^Chi-square test.

^c^Mixed effect models, adjusted for age, sex, disease duration and including the scanner as random effect.

^d^Linear regression models adjusted for age, sex, disease duration.

^e^Normalized volumes are obtained by dividing individual volumes by the total intracranial volume.

### Disability profiles are associated with conventional whole-brain MRI volumetric measures

People with cognitive disability profile had higher lesion volumes than those in the other two subgroups. Both global and cognitive disability profiles were associated with lower normalized brain, grey matter and white matter volume compared to the motor disability group. Patients with global disability profile also had lower normalized whole brain and grey matter volumes compared to those with cognitive disability profile and lower intracranial volume compared to the motor disability profile (Table 2 and Supplementary Fig. 3 report detailed comparisons of conventional MRI measures among the different disability profiles).

### Regional grey matter volumes differ between disability profiles

Across disability profiles, the clearest separation of the motor disability group from the remaining was in subcortical volume loss. The cognitive disability profile showed more pronounced loss in the thalamus (β = −0.242, SE = 0.051; FDR-corrected *P* < 0.001) and ventral diencephalon (β = −0.217, SE = 0.051; FDR-corrected *P* < 0.001), together with basal-ganglia reductions—caudate (β = −0.172, SE = 0.051; FDR-corrected *P* = 0.001), pallidum (β = −0.152, SE = 0.051; FDR-corrected *P* = 0.001–0.005) and right putamen (β = −0.109, SE = 0.051; FDR-corrected *P* = 0.032). Cortical effects were smaller but consistent, with lower volumes in the posterior cingulate (β = −0.116, SE = 0.051; FDR-corrected *P* = 0.014–0.038), middle temporal cortex (β = −0.113, SE ≈ 0.051; FDR-corrected *P* = 0.021–0.031), middle cingulate (β = −0.127, SE = 0.052; FDR-corrected *P* = 0.004–0.048) and the supplementary motor cortex (β = −0.161, SE = 0.051; FDR-corrected *P* ≤ 0.001–0.007).

The global disability profile mirrored these subcortical losses—again involving the thalamus (β = −0.229, SE = 0.052; FDR-corrected *P* < 0.001) and ventral diencephalon (β = −0.211, SE = 0.052; FDR-corrected *P* < 0.001)—and extended further across the basal ganglia. Cortical reductions were broader, spanning the posterior cingulate (β = −0.136, SE = 0.052; FDR-corrected *P* = 0.002–0.038), middle temporal (β = −0.138, SE ≈ 0.052; FDR-corrected *P* = 0.004–0.016), middle cingulate (β = −0.117, SE = 0.053; FDR-corrected *P* = 0.027–0.030) and supplementary motor regions (β = −0.176, SE = 0.052; FDR-corrected *P*≤0.001). Infratentorial and posterior involvement was also evident, with loss in the pons (β = −0.209, SE = 0.053; FDR-corrected *P* < 0.001), brainstem (β = −0.139, SE = 0.052; FDR-corrected *P* = 0.007), cerebellar cortex (β = −0.147, SE = 0.052; FDR-corrected *P* = 0.004–0.006), and precuneus (β = −0.148, SE = 0.052; FDR-corrected *P*≤0.001–0.040), together with the medial superior frontal gyrus (β = −0.141, SE = 0.051; FDR-corrected *P* = 0.001–0.026).

Head-to-head contrasts indicated more severe volume loss in global disability compared to cognitive disability profile in vermal lobules I–V (β = 0.102, SE = 0.046, FDR-corrected *P* = 0.026), right cuneus (β = 0.096, SE = 0.045, FDR-corrected *P* = 0.033), precuneus (β = 0.095, SE = 0.045, FDR-corrected *P* = 0.036), middle occipital gyrus (β = 0.110, SE = 0.045, FDR-corrected *P* = 0.015) and left medial superior frontal gyrus (β = 0.114, SE = 0.044, FDR-corrected *P* = 0.009). By contrast, cognitive disability profile had more severe volume loss in the right gyrus rectus (β = −0.104, SE = 0.046, FDR-corrected *P* = 0.023) and left posterior orbital gyrus (β = −0.093, SE = 0.044, FDR-corrected *P* = 0.036). All comparisons are reported in Supplementary Table 3.

### ICA-derived patterns of regional grey matter volumes differ between disability profiles

**Figure 3:**
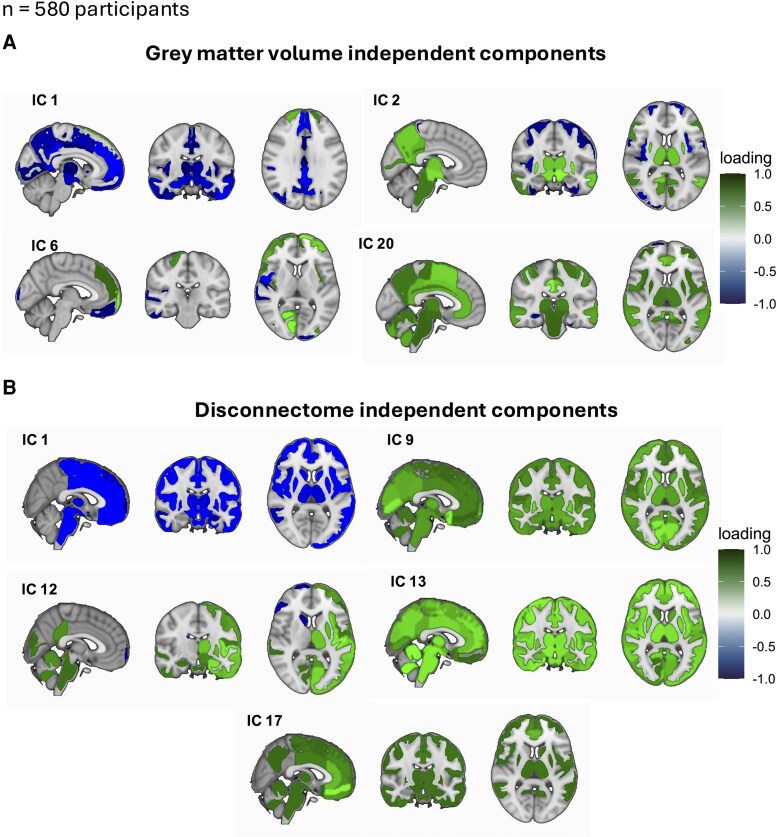
**Independent component analysis.** Summarizes the regional loadings contributing to independent components (ICs) derived from volumes (A) and of disconnection (B). Only ICs whose component scores differed significantly among the newly defined disability profiles are shown. Loadings are displayed on a blue-green scale ranging from −1 to 1, with negative loadings in blue and positive loadings in green. Top 10 regions by loading for components in (A) Top 10 regions by loading: IC1: left anterior orbital gyrus: −0.424, left inferior temporal gyrus: −0.418, right superior frontal gyrus, medial segment: −0.409, left posterior cingulate gyrus: −0.394, left entorhinal area: −0.391, left temporal pole: −0.390, right anterior orbital gyrus: −0.379, left gyrus rectus: −0.363, left amygdala: −0.352, left frontal pole: −0.341; IC2: left anterior insula: −0.375, left ventral diencephalon: 0.356, right putamen: 0.332, left putamen: 0.328, right ventral diencephalon: 0.322, right superior occipital gyrus: −0.309, right anterior insula: −0.302, left middle temporal gyrus: 0.300, left superior frontal gyrus: −0.296, right precuneus: 0.296; IC6: right gyrus rectus: −0.421, right central operculum: −0.371, right orbital part of inferior frontal gyrus: −0.333, right calcarine cortex: 0.321, left frontal pole: 0.320, right medial orbital gyrus: −0.320, right medial frontal cortex: −0.305, left middle frontal gyrus: 0.279, left superior frontal gyrus: 0.259, right triangular part of inferior frontal gyrus: 0.237; IC20: right middle cingulate gyrus: 0.513, left supplementary motor cortex: 0.421, right anterior cingulate gyrus: 0.395, left middle cingulate gyrus: 0.382, right posterior cingulate gyrus: 0.369, left anterior cingulate gyrus: 0.352, left posterior cingulate gyrus: 0.346, right supplementary motor cortex: 0.321, left middle temporal gyrus: 0.315, left cerebellum exterior: 0.312. Top 10 regions by loading for components in (B): IC1: left lingual gyrus: −0.195, right lingual gyrus: −0.190, right cuneus: −0.174, left cuneus: −0.164, left calcarine cortex: −0.163, right calcarine cortex: −0.160, left middle occipital gyrus: −0.145, right middle occipital gyrus: −0.144, left superior occipital gyrus: −0.137, right superior occipital gyrus: −0.127; IC9: left angular gyrus: 0.227, right angular gyrus: 0.222, right precuneus: 0.213, left precuneus: 0.210, left posterior cingulate gyrus: 0.205, right posterior cingulate gyrus: 0.202, left superior frontal gyrus medial segment: 0.193, right superior frontal gyrus medial segment: 0.185, left middle temporal gyrus: 0.178, right middle temporal gyrus: 0.171; IC12: left inferior frontal gyrus triangular part: 0.194, right inferior frontal gyrus triangular part: 0.189, left opercular part of inferior frontal gyrus: 0.176, right opercular part of inferior frontal gyrus: 0.172, left middle frontal gyrus: 0.160, right middle frontal gyrus: 0.157, right frontal pole: 0.145, left frontal pole: 0.140, right insula: 0.137, left insula: 0.134; IC13: right postcentral gyrus: −0.202, left postcentral gyrus: −0.196, right precentral gyrus: −0.188, left precentral gyrus: −0.182, left paracentral lobule: −0.173, right paracentral lobule: −0.171, left supplementary motor area: −0.165, right supplementary motor area: −0.162, left superior parietal lobule: −0.154, right superior parietal lobule: −0.149; IC17: left cerebellum crus I: 0.240, right cerebellum crus I: 0.235, left cerebellum VI: 0.223, right cerebellum VI: 0.218, vermis VI: 0.211, vermis VII: 0.203, left cerebellum crus II: 0.198, right cerebellum crus II: 0.194, left cerebellum lobule VIII: 0.180, right cerebellum lobule VIII: 0.178. Abbreviations: IC = independent component.

We identified 20 independent components globally explaining the 80% of variance among the regional grey matter volumetric measures. Significant differences in ICA loadings of regional volumetric patterns among the newly identified disability profiles are summarized in Table 3, while all the comparisons are summarized in Supplementary Table 4. Briefly, a small set of components best separated the profiles: default mode network (DMN) (IC1), thalamo–striatal/posterior-DMN–temporal network (IC2), prefrontal–visual integration network (IC6), and medial motor/control network (IC20). Relative to the motor group, the cognitive profile showed a selective reduction in IC2. By contrast, the global profile showed higher IC1 and lower IC2 and IC20 loadings than motor profiles consistent with widespread limbic/default-mode–frontal reweighting alongside medial motor/control network volume loss. Compared to global disability profile, the cognitive disability profile showed more severe volume loss in the prefrontal–visual integration network. The contribution of individual region volumes to IC loadings showing significant differences among the disability profiles is represented in Fig. 3A, while a graphical representation of all 20 components is reported in Supplementary Fig. 4.

**Table 3:** Summarizes significant differences in IC loadings of regional volume patterns among the disability profiles

	Cognitive disability versus Motor disability			Global disability versus Motor disability			Cognitive disability versus Global disability		
	β coef	SE	FDR-corrected *P* values	β coef	SE	FDR-corrected *P* values	β coef	SE	FDR-corrected *P* values
IC 1	0.075	0.047	0.441	0.153	0.048	**0**.**023**	−0.067	0.041	0.441
IC 2	−0.204	0.051	**0**.**004**	−0.151	0.052	**0**.**043**	−0.063	0.044	0.446
IC 6	0.100	0.052	0.289	−0.060	0.053	0.526	0.156	0.045	**0**.**018**
IC 20	−0.125	0.051	0.095	−0.171	0.052	**0**.**020**	0.034	0.044	0.666

Abbreviations: FDR = false discovery rate; IC = independent component; SE = standard error.

Bold type indicates statistical significance.

Top 10 regions by loading: IC1: left anterior orbital gyrus: −0.424, left inferior temporal gyrus: −0.418, right superior frontal gyrus, medial segment: −0.409, left posterior cingulate gyrus: −0.394, left entorhinal area: −0.391, left temporal pole: −0.390, right anterior orbital gyrus: −0.379, left gyrus rectus: −0.363, left amygdala: −0.352, left frontal pole: −0.341; IC2: left anterior insula: −0.375, left ventral diencephalon: 0.356, right putamen: 0.332, left putamen: 0.328, right ventral diencephalon: 0.322, right superior occipital gyrus: −0.309, right anterior insula: −0.302, left middle temporal gyrus: 0.300, left superior frontal gyrus: −0.296, right precuneus: 0.296; IC6: right gyrus rectus: −0.421, right central operculum: −0.371, right orbital part of inferior frontal gyrus: −0.333, right calcarine cortex: 0.321, left frontal pole: 0.320, right medial orbital gyrus: −0.320, right medial frontal cortex: −0.305, left middle frontal gyrus: 0.279, left superior frontal gyrus: 0.259, right triangular part of inferior frontal gyrus: 0.237; IC20: right middle cingulate gyrus: 0.513, left supplementary motor cortex: 0.421, right anterior cingulate gyrus: 0.395, left middle cingulate gyrus: 0.382, right posterior cingulate gyrus: 0.369, left anterior cingulate gyrus: 0.352, left posterior cingulate gyrus: 0.346, right supplementary motor cortex: 0.321, left middle temporal gyrus: 0.315, left cerebellum exterior: 0.312.

### Disability profiles are associated with whole-brain measures of disconnection

The cognitive disability group showed more marked whole-brain disconnection compared to the motor disability (0.527 ± 0.183 versus 0.352 ± 0.166, β = 0.421 SE = 0.050, FDR-corrected *P* < 0.001) and global disability subgroups (versus 0.469 ± 0.191, β = 0.157, SE = 0.042, FDR-corrected *P* < 0.001), and those with global disability also had more disconnection compared to the motor disability group (β = 0.282, SE = 0.050, FDR-corrected *P* < 0.001).

### Regional disconnection differs among disability profiles

Supplementary Table 5 summarizes all comparisons in regional disconnections among the different profiles. Compared to the motor disability profile, the cognitive disability profile showed significantly greater disconnection across all regions explored, with the largest effects in the cerebellum (β = 0.404, SE = 0.049, FDR-corrected *P* < 0.001), thalamus (β = 0.411, SE = 0.049, FDR-corrected *P* < 0.001) and ventral diencephalon (β = 0.411, SE = 0.049, FDR-corrected *P* < 0.001). The global disability profile likewise exhibited widespread disconnection relative to the motor group, albeit of smaller magnitude. Direct comparison of cognitive versus global disability profile identified significantly greater disconnection in the cognitive group in the pons (β = 0.164, SE = 0.043, FDR-corrected *P* = 0.050), brainstem (β = 0.170, SE = 0.043, FDR-corrected *P* = 0.029), right posterior insula (β = 0.180, SE = 0.044, FDR-corrected *P* = 0.016), right thalamus (β = 0.169, SE = 0.043, FDR-corrected *P* = 0.035), right anterior insula (β = 0.175, SE = 0.044, FDR-corrected *P* = 0.028), right middle frontal gyrus (β = 0.177, SE = 0.043, FDR-corrected *P* = 0.019), right precentral gyrus (β = 0.167, SE = 0.043, FDR-corrected *P* = 0.045), right superior frontal gyrus (β = 0.174, SE = 0.043, FDR-corrected *P* = 0.025), right transverse temporal gyrus (β = 0.169, SE = 0.044, FDR-corrected *P* = 0.049) and left temporal pole (β = 0.167, SE = 0.043, FDR-corrected *P* = 0.045). No other regions differed significantly between the two disability profiles.

### ICA-derived patterns of regional disconnection differ between disability profiles

We identified 20 independent components globally explaining the 90% of variance among the regional disconnection. Significant differences in ICA loadings of regional disconnection patterns among the newly identified disability profiles are summarized in Table 4, while all the comparisons are summarized in Supplementary Table 6. Briefly, a small set of disconnection components best separated the profiles: occipital/visual network (IC1), DMN (IC9), fronto-insular/cingulo-opercular control network (IC12), sensorimotor network axis (IC13) and cerebellar–vermian/brainstem network (IC17). Relative to the motor disability profile, the cognitive disability profile showed selectively higher loadings on IC9, IC12 and IC13, corresponding to more severe disconnection in DMN and fronto-insular/cingulo-opercular control network, with relatively preserved connectivity in the sensorimotor network. By contrast, the global disability profile showed higher loadings of IC9, IC13 and IC17 corresponding to greater disconnection in DMN and cerebellar network, with relatively preserved connectivity in the sensorimotor network. Compared to the global disability profile, the cognitive disability profile showed greater connectivity in sensorimotor network (IC13) and occipital/visual network (IC1).

**Table 4:** Summarizes significant differences in IC loadings of regional disconnection patterns

	Cognitive disability versus Motor disability			Global disability versus Motor disability			Cognitive disability versus Global disability		
	β coef	SE	FDR-corrected *P* values	β coef	SE	FDR-corrected *P* values	β coef	SE	FDR-corrected *P* values
IC 1	−0.094	0.052	0.207	0.038	0.053	0.735	−0.130	0.045	**0**.**033**
IC 9	0.147	0.051	0.033	0.176	0.052	**0**.**013**	−0.017	0.045	0.927
IC 12	0.200	0.052	**0**.**004**	0.117	0.053	0.136	0.090	0.045	0.167
IC 13	0.299	0.050	**0**.**000**	0.174	0.051	**0**.**013**	0.136	0.044	**0**.**026**
IC 17	−0.075	0.052	0.446	0.146	0.053	**0**.**041**	0.017	0.046	0.927

Abbreviations: IC = independent component; FDR = false discovery rate; SE = standard error.

Bold type indicates statistical significance.

Top 10 regions by loading: IC1: left lingual gyrus: −0.195, right lingual gyrus: −0.190, right cuneus: −0.174, left cuneus: −0.164, left calcarine cortex: −0.163, right calcarine cortex: −0.160, left middle occipital gyrus: −0.145, right middle occipital gyrus: −0.144, left superior occipital gyrus: −0.137, right superior occipital gyrus: −0.127; IC9: left angular gyrus: 0.227, right angular gyrus: 0.222, right precuneus: 0.213, left precuneus: 0.210, left posterior cingulate gyrus: 0.205, right posterior cingulate gyrus: 0.202, left superior frontal gyrus medial segment: 0.193, right superior frontal gyrus medial segment: 0.185, left middle temporal gyrus: 0.178, right middle temporal gyrus: 0.171; IC12: left inferior frontal gyrus triangular part: 0.194, right inferior frontal gyrus triangular part: 0.189, left opercular part of inferior frontal gyrus: 0.176, right opercular part of inferior frontal gyrus: 0.172, left middle frontal gyrus: 0.160, right middle frontal gyrus: 0.157, right frontal pole: 0.145, left frontal pole: 0.140, right insula: 0.137, left insula: 0.134; IC13: right postcentral gyrus: −0.202, left postcentral gyrus: −0.196, right precentral gyrus: −0.188, left precentral gyrus: −0.182, left paracentral lobule: −0.173, right paracentral lobule: −0.171, left supplementary motor area: −0.165, right supplementary motor area: −0.162, left superior parietal lobule: −0.154, right superior parietal lobule: −0.149; IC17: left cerebellum crus I: 0.240, right cerebellum crus I: 0.235, left cerebellum VI: 0.223, right cerebellum VI: 0.218, vermis VI: 0.211, vermis VII: 0.203, left cerebellum crus II: 0.198, right cerebellum crus II: 0.194, left cerebellum lobule VIII: 0.180, right cerebellum lobule VIII: 0.178.

The contribution of disconnection in individual regions to IC loadings showing significant differences among the disability profiles is represented in Fig. 3B, while the graphical representation of all 20 components is reported in Supplementary Fig. 5.

### Regional rather than whole-brain MRI measures contribute most to disability profile separation

**Figure 4:**
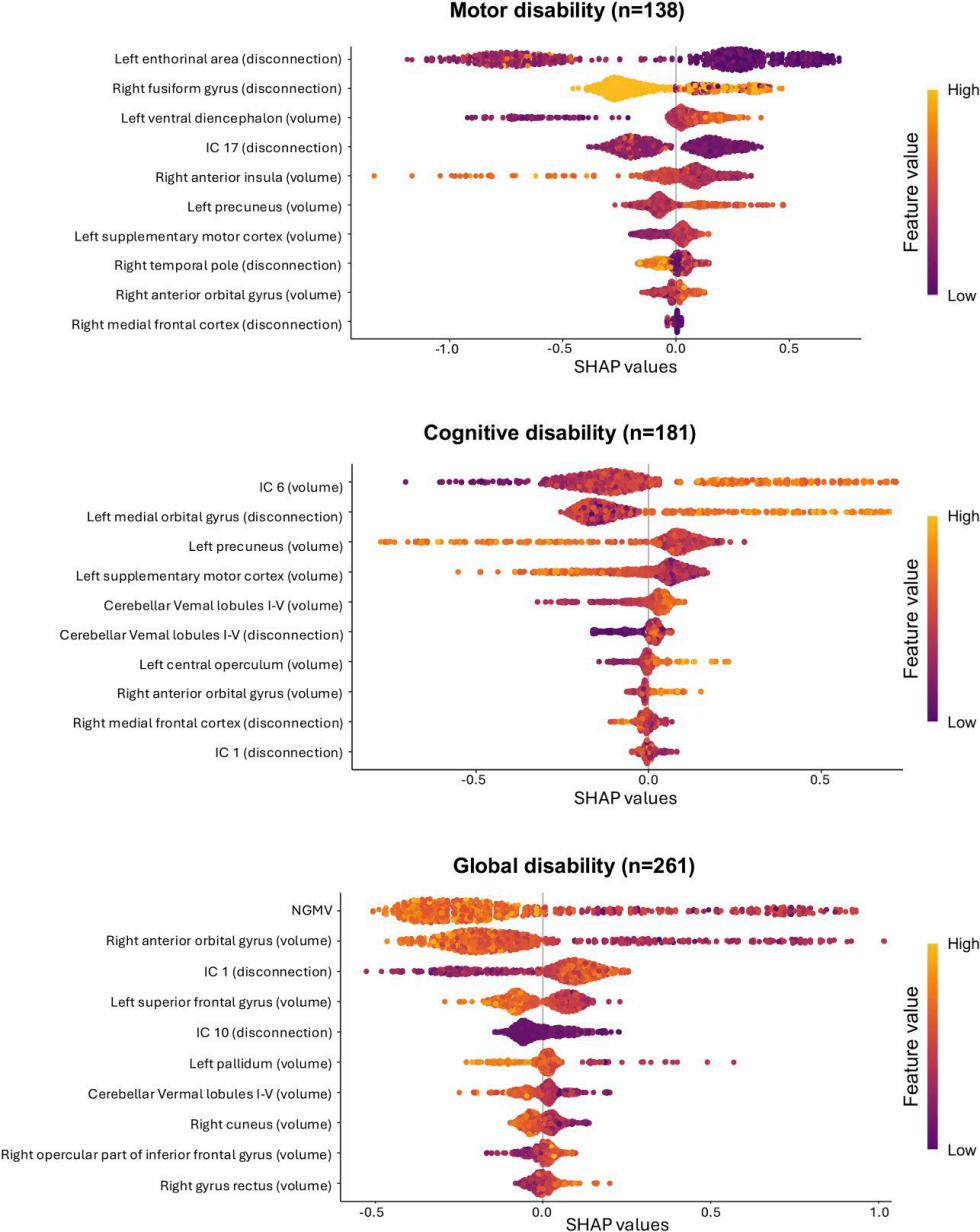
**SHAP beeswarm plots for disability phenotypes.** Each dot represents an individual observation. Dot colour reflects feature value (purple = low, yellow = high). The *x*-axis shows SHapley Additive exPlanations (SHAP) values (feature contribution to the model output for that phenotype): positive values increase the probability of classification into the target class, negative values reduce it. Distance from zero indicates contribution magnitude. Features on the *y*-axis are ordered by mean absolute SHAP value (most influential at the top). Vertical spread reflects inter-individual variability and non-linear/interaction effects. Abbreviations: IC = independent component; NGMV = normalized grey matter volume; SHAP = SHapley Additive exPlanations.

In a combined model of whole brain, regional, and ICA components (derived from tissue volumes and disconnection measures), the motor disability profile was characterized by higher connectivity and volume in regions belonging to limbic and frontal networks, and by preserved volumes in supplementary motor cortex volume and postcentral gyrus, relative to the other profiles. The cognitive disability profile was characterized by volume loss and disconnection in cerebellar vermal lobules, temporal pole, posterior cingulate gyrus and parietal operculum, together with one disconnection IC (lower loading of IC1) and one grey matter volume IC (higher loading of IC6). The global disability profile was primarily influenced by lower normalized grey matter volume, along with two disconnection ICs (higher loading of IC1 and IC10), and lower volumes in the superior frontal gyrus, pallidum, cuneus and cerebellum. Figure 4 shows beeswarm plots for each disability profile, summarizing magnitude and direction of each feature’s contribution to disability profile characterization. Results of the sensitivity analyses are summarized in Supplementary Fig. 6.

## Discussion

In a large cohort of patients with progressive MS, we identified for the first time different (latent) impairment profiles which are largely independent from clinical phenotypes, and that are characterized by differential patterns of brain damage, including regional grey matter atrophy and disconnected networks. These findings suggest that clinical heterogeneity can be explained by latent non-random patterns.

### Clinically relevant LPA profiles

Our analysis identified three profiles, one with mild disability across multiple neurological and cognitive parameters, one with marked disability across multiple neurological and cognitive measures (largest cohort) and one with moderately impaired physical function but marked cognitive impairment. Disability profiles did not differ in age or sex distribution but a higher proportion of SPMS patients, and people with longer disease durations were observed in the global disability subgroup. However, this was not confirmed when examining the distribution of clinical phenotypes across our newly defined disability profiles after matching PPMS and SPMS groups for EDSS and disease duration. These findings further indicate that the disability profiles capture variability in clinical manifestations that are not reflected by conventional PPMS and SPMS phenotypes.^47^

Although the global disability profile was characterized by a longer disease duration compared to both the cognitive and motor disability profiles, this does not necessarily indicate that it represents a more advanced stage along a shared disease trajectory. As example, if the global disability profile had evolved from the cognitive disability profile, one would expect similar MRI features to be present. However, the two profiles showed distinct MRI characteristics, suggesting that they may reflect divergent rather than sequential disease pathways. Taken together, these observations lead us to hypothesize that these groups are distinct, both in terms of clinical outcomes and the regional distribution of disconnection and volume loss, thus not simply a continuum differing only by disease duration. By contrast, we cannot draw an equivalent inference for the motor-disability profile: it shows milder cognitive and motor impairment and more limited disconnection and atrophy and may represent an earlier state that could evolve into one of the other profiles, although this requires longitudinal confirmation. In future work, it would be of interest to assess longitudinal clinical changes, to determine if the motor disability group remains stable or transitions and confirm that the global and cognitive disability groups remain distinct and tend to progress in the clinical domains already most affected. Our findings suggest that they will, and this has important implications for the way in which we assess clinical progression. Specifically for the group with cognitive impairment, EDSS scores will be inadequate and in routine clinical practice, where cognition is not typically assessed, progressive cognitive deficits will be missed.

Indeed, although the EDSS is the most commonly used measure of clinical disability in both routine care and research, it is heavily weighted towards motor function and ambulation and therefore fails to capture more subtle, yet functionally important, changes such as upper limb impairment, fatigue and cognitive dysfunction.^2^ This limitation is even more relevant in progressive MS, where ambulation is the main determinant of EDSS scores, while changes in cognitive functioning (which are not adequately reflected by the EDSS) are both common and clinically meaningful.^48^

### MRI patterns of pathology differ between LPA profiles

In characterizing the disability profiles, we began at the global level (whole brain) and progressively narrowed our focus to the individual brain regions while also examining patterns of regional volumes and disconnection across groups of regions. A key finding across all levels of analysis was that the cognitive disability profile was consistently associated with higher lesion burden and, consequently, greater disconnection across the brain. In contrast, the global disability profile demonstrated a stronger association with grey matter atrophy. It is important to note that our cognitive assessment focused on the SDMT and BVMT-R, while other cognitive domains not captured by these measures may correlate more closely with regional tissue loss than with disconnection. Nonetheless, our results underscore the value of this approach in identifying clinically meaningful disease subtypes and linking them to distinct underlying pathological mechanisms—findings which may have important implications for treatment responsiveness. Moreover, they align with the prevailing view that grey matter neurodegeneration in MS is only partially lesion-dependent, as supported by previous studies.^49^

Considering regional MRI findings in the disability profiles, differences in grey matter volumes and disconnection distinguished them. The cognitive disability profile was characterized by greater disconnection and volume loss in brain regions encompassing fronto-parietal network and DMN, playing a major role in cognitive function.^50,51^ Among these, the prefrontal cortex plays a crucial role in executive functions,^52^ working memory,^53^ attention regulation and cognitive flexibility,^54^ while precuneus and anterior insula regulate the balance between internal and external attention.^55^ Finally, another key region whose disconnection and volume loss significantly contributed to this profile was the cerebellum, underscoring once again its role in cognitive processing alongside its well-known contribution to motor coordination.^56^

The global disability profile was characterized by global grey matter atrophy, alongside volume loss in regions known to be associated with both cognitive and motor outcomes. This pattern reflects a widespread neurodegenerative process, where the involvement of the prefrontal cortex and cerebellum may contribute to cognitive impairment^50,56^ (albeit less severe than in the cognitive disability profile) while basal ganglia degeneration and broader grey matter atrophy likely drive the motor dysfunction and overall neurological disability in this group. Indeed, both grey matter volume^57^ and basal ganglia^58^ are known to contribute to performance in the 25FWT and 9HPT, with poorer performance in these tests, and higher EDSS scores, characterizing this profile. In line with the established association between frontoparietal integrity and performance on visuospatial tasks,^59^ the global disability profile also showed more severe frontoparietal disconnection. Conversely, connectivity within the visual network was relatively preserved, distinguishing this profile from the cognitive disability profile and consistent with less impaired SDMT performance, which depends on rapid visual encoding and visuo-attentional scanning.^60^

A lower intracranial volume (a potential proxy of brain reserve)^61^ was observed in the global disability compared to the motor disability group. It is also worth noting that brain reserve showed a protective effect only against the accrual of neurological disability and of brain atrophy, as previously observed,^61^ while other factors like cognitive reserve and its proxies (premorbid intelligence quotient, leisure activities and education)^62^ need to be considered while looking at cognitive performance.

### Regional volumes and disconnection drive profile classification

We used a SHAP analysis approach to determine the relative contribution of the whole brain, regional and independent component features to differences between the three disability profiles. This yields a model where the relative contribution of each feature can be directly assessed. Another approach is LASSO, typically used to produce an optimized model, removing features that do not significantly contribute to its predictive power. Indeed, as a sensitivity analysis, we also ran LASSO using the same input features (Supplemental Materials). Although the modelling approaches differ—with LASSO prioritizing sparsity and linear feature selection, and SHAP offering a more interpretable framework that captures non-linear effects, feature interactions and stable contributions at both the global and individual level—both consistently identified regional MRI features as important. This convergence reinforces the conclusions drawn from the SHAP analysis, highlighting the relevance of regional MRI measures in distinguishing subgroups of individuals with MS who exhibit similar clinical outcomes.

### Potential applications

If replicated longitudinally and validated in independent cohorts, these disability profiles could underpin more personalized care. Profile assignment may inform treatment choice and sequencing. Monitoring precision could be improved by tracking profile-specific biomarkers and network-informed MRI indices (component loadings and regional volumes/disconnection) alongside appropriate clinical measures, including standardized cognitive testing that the EDSS does not capture. From a research perspective, these profiles offer a basis for trial stratification and enrichment, alignment of endpoints with affected networks and hypothesis-driven evaluation of treatment–mechanism links. They also may help identify prioritizing treatment targets and profile-defined subgroups.

### Limitations

This study has several limitations. Firstly, as participants were recruited within an industry-sponsored clinical trial, selection effects and protocol-driven exclusions may limit cohort representativeness and the generalisability of our findings. We did not include healthy participants, so we could only assess relative clinical and MRI differences between MS subgroups, not absolute abnormalities. Additionally, the number of cognitive tests was limited, preventing a more detailed characterization of cognitive performance. In particular, California Verbal Learning Test (the third component of the Brief International Cognitive Assessment for Multiple Sclerosis) was not included because of the trial’s multilingual range across centres. For the same reason, we did not use norm-based z-scores, characterized by cross-site/language heterogeneity. While this does not undermine the key findings of this study, given that distinct cognitive profiles, each associated with different pathological substrates, have also been identified in a phenotypically mixed cohort,^3^ it is likely that with more clinical measures, more clinical subtypes could be found. Another potential limitation of our study lies in the choice of clustering methodology. While numerous clustering approaches exist—ranging from k-means and hierarchical clustering to Gaussian mixture models and more recent deep learning–based techniques—validating the biological and clinical relevance of clustering solutions remains inherently challenging. We employed LPA, a model-based probabilistic approach allowing for the inclusion of continuous variables and accounting for heterogeneity within the population by estimating the probability of membership for each individual, rather than forcing deterministic classification. Additionally, LPA provides statistical criteria (e.g. BIC, AIC) to guide model selection, enhancing reproducibility and interpretability. While no clustering method is without limitations, the use of LPA in this context strengthens the rigour and clinical relevance of our subgroup identification, particularly given the complexity and overlapping features of MS phenotypes.

From the MRI point of view, as first, we could not employ data harmonization methods due to the small number of participants at each site (we included scanner model as a covariate in our analysis); however, this will have added noise rather than bias to the data and so cannot plausibly account for our findings. For our disconnectome analysis, we used an atlas-based approach and could not run a tractography-based analysis, as we did not obtain diffusion tensor imaging data, so we could not complement proportion-of-tract disconnection with indices of microstructural damage. Furthermore, our voxelwise analyses rely on non-linear registration to a common template, which can be suboptimal in people with substantial white matter atrophy. Although all registrations were carefully quality-controlled, residual misalignment in severely atrophic brains cannot be excluded but would be expected to dilute rather than inflate regional group differences. Finally, the profiles we identified were derived from clinical measures; although we observed differing MRI substrates across profiles, a substantial degree of overlap likely remains between the underlying pathological processes.

## Conclusion

In conclusion, we have shown distinct disability subgroups that are seen across the PPMS and SPMS spectrum, which are better explained by regional rather than global brain pathology. If validated prospectively and in independent cohorts, these profiles could inform clinical monitoring (e.g. adding standardized cognitive testing where relevant), aid personalized treatment decisions and enable biomarker-driven trial stratification.

## Supplementary Material

fcag162_Supplementary_Data

## Data Availability

Data are available from Roche upon reasonable request.
