# Supplementary material for: Disability profiles in progressive multiple sclerosis reflect pathology distribution, independent of clinical phenotype
Source: Brain Commun. 2026 May 2;8(3):fcag162. doi: 10.1093/braincomms/fcag162 (PMC13201091; doi:10.1093/braincomms/fcag162)
Supplement: fcag162_Supplementary_Data [file fcag162_supplementary_data.zip › Supplementary_material.docx]

***Supplementary material***

- **Supplementary Table 1.** MRI acquisition parameters for the different study sites.
- **Propensity Matching**
- **Supplementary Table 2.** LPA model fit indices for model for 1 to 10 profiles
- **Supplementary Figure 1.** Clinical features of the disability profiles.
- **Supplementary Figure 2.** Propensity matching of primary and secondary progressive multiple sclerosis according to Expanded Disability Status Scale and disease duration.
- **Supplementary Figure 3.** Conventional MRI features of the disability profiles.
- **Supplementary Table 3.** Summary of regional volume comparisons across the disability profiles
- **Supplementary Figure 4.** Independent components derived from of grey matter regional volumes
- **Supplementary Table 4.** Summary of all comparisons of Independent Component loadings for regional volume patterns across the disability profiles
- **Supplementary Table 5.**  Summary of all regional disconnection comparisons among the disability profiles
- **Supplementary Figure 5.** Independent components derived from of grey matter regional disconnection
- **Supplementary Table 6.** Summary of all Independent Component loading comparisons for regional disconnection patterns across defined disability phenotypes
- **Sensitivity analysis: (Latent Profile Analysis) LPA Profiles in PP and SPMS**
- **Supplementary Figure 6.** Clinical scores across disability profiles in PP and SPMS
- **Supplementary Table 7.** Summary of disability profile × clinical phenotype (SPMS vs PPMS) interaction terms for regional volumetric measures.
- **Supplementary Table 8.** Summary of disability profile × clinical phenotype (SPMS vs PPMS) interaction terms for Independent Component loadings of regional volume patterns.
- **Supplementary Table 9.** Summary of disability profile × clinical phenotype (SPMS vs PPMS) interaction terms for regional disconnection
- **Supplementary Table 10.** Summary of disability profile × clinical phenotype (SPMS vs PPMS) interaction terms for independent component loadings of regional disconnection patterns.
- **Sensitivity analysis: feature selection**
- **Supplementary Figure 7.** Summarizes selected features by using LASSO for each disability profile

**Supplementary Table 1.** MRI acquisition parameters for the different study sites.

| **2D T2-weighted** | | | | | | | | | | | | | | |
| --- | --- | --- | --- | --- | --- | --- | --- | --- | --- | --- | --- | --- | --- | --- |
|  | **Sequence type** | **TR (ms)** | **TE (ms)** | **ETL/Turbo factor** | **FOV (APxRL)** | | | | **Matrix** | | | | **Slice thickness (mm)** | |
| **Siemens 1.5T** | 2D TSE | 5980 or 5000 | 80 | 1 | AP:256  RL:192 | | | | 256 | | | | 3 | |
| **Siemens 3T** | 2D TSE | 4500 | 80 | 11 | AP:256  RL:192 | | | | 256 | | | | 3 | |
| **GE 1.5T** | FSE-XL | 5120 | 80 | 8 | AP:256  RL:192 | | | | 256x256 | | | | 3 | |
| **GE 3T** | FSE-XL | 5300 | 70 | 8 | AP:256  RL:192 | | | | 256x256 | | | | 3 | |
| **Philips 1.5T** | 2D TSE | 4900 | 80 | 8 | AP:256  RL:192 | | | | 256 | | | | 3 | |
| **Philips 3T** | 2D TSE | 6100 | 80 | 8 | AP:256  RL:192 | | | | 256 | | | | 3 | |
| **Toshiba 1.5T** | 2D FSE | 5000 | 75 | 9 | AP:256  RL:192 | | | | 256 | | | | 3 | |
| **2D PD-weighted** | | | | | | | | | | | | | | |
|  | **Sequence type** | **TR (ms)** | **TE (ms)** | **ETL/Turbo factor** | **FOV (APxRL)** | **Matrix** | | | | **Slice thickness (mm)** | | | | |
| **Siemens 1.5T** | 2D TSE | 2980 or 2200 | 12 | 3 | AP:256  RL:192 | 256 | | | | 3 | | | | |
| **Siemens 3T** | 2D TSE | 2200 | 9.2 | 4 | AP:256  RL:192 | 256 | | | | 3 | | | | |
| **GE 1.5T** | FSE-XL | 2200 | 12 | 3 | AP:256  RL:192 | 256x256 | | | | 3 | | | | |
| **GE 3T** | FSE-XL | 2200 | 8 | 3 | AP:256  RL:192 | 256x256 | | | | 3 | | | | |
| **Philips 1.5T** | 2D TSE | 2200 | 15 | 3 | AP:256  RL:192 | 256 | | | | 3 | | | | |
| **Philips 3T** | 2D TSE | 2200 | 10 | 3 | AP:256  RL:192 | 256 | | | | 3 | | | | |
| **Toshiba 1.5T** | 2D FSE | 2000 | 12 | 4 | AP:256  RL:192 | 256 | | | | 3 | | | | |
| **2D FLAIR** | | | | | | | | | | | | | | |
|  | **Sequence type** | **TR (ms)** | **TE (ms)** | **TI**  **(ms)** | **ETL/Turbo factor** | | **FOV (APxRL)** | | | | **Matrix** | | | **Slice thickness (mm)** |
| **Siemens 1.5T** | 2D TSE-IR | 9000 9400 | 80 | 2500 | 9 | | AP:256  RL:192 | | | | 256 | | | 3 |
| **Siemens 3T** | 2D TSE-IR | 9000 | 80 | 2500 | 9 | | AP:256  RL:192 | | | | 256 | | | 3 |
| **GE 1.5T** | 2D T2-FLAIR | 9000 | 80 | 2250 | 8 | | AP:256  RL:192 | | | | 256x256 | | | 3 |
| **GE 3T** | T2-FLAIR | 9000 | 80 | 2500 | 10 | | AP:256  RL:192 | | | | 256x256 | | | 3 |
| **Philips 1.5T** | 2D TIR | 9000 | 80 | 2500 | 9 | | AP:256  RL:192 | | | | 256 | | | 3 |
| **Philips 3T** | 2D TIR | 9000 | 80 | 2500 | 12 | | AP:256  RL:192 | | | | 256 | | | 3 |
| **Toshiba 1.5T** | 2D FSE+15 nBW slt | 9000 | 90 | 2500 | 11 | | AP:256  RL:192 | | | | 256 | | | 3 |
| **3D T1-weighted** | | | | | | | | | | | | | | |
|  | **Sequence type** | **TR (ms)** | **TE (ms)** | **Flip angle (°)** | **ETL/Turbo factor** | | **FOV (APxRL)** | | | | **Matrix** | | | **Slice thickness (mm)** |
| **Siemens 1.5T** | 3D FLASH | 30 | 11 | 30 | 9 | | AP:256  RL:192 | | | | 256 | | | 3 |
| **Siemens 3T** | 3D FLASH | 28 | 6 | 27 | 9 | | AP:256  RL:192 | | | | 256 | | | 3 |
| **GE 1.5T** | 3D SPGR | 30 | 7 | 30 | 8 | | AP:256  RL:192 | | | | 256x256 | | | 3 |
| **GE 3T** | 3D SPGR | 30 | 6 | 27 | 10 | | AP:256  RL:192 | | | | 256x256 | | | 3 |
| **Philips 1.5T** | 3D FFE with “T1 contrast” enabled | 30 | 7 | 30 | 9 | | AP:256  RL:192 | | | | 256 | | | 3 |
| **Philips 3T** | 3D FFE with “T1 contrast” enabled | 28 | 4 | 27 | 12 | | AP:256  RL:192 | | | | 256 | | | 3 |
| **Toshiba 1.5T** | FE3D fc | 30 | 7 | 30 | 11 | | AP:256  RL:192 | | | | 256 | | | 3 |
| **3D-Leptomeningeal FLAIR** | | | | | | | | | | | | | | |
|  | **Sequence type** | **TR (ms)** | **TE (ms)** | **TI**  **(ms)** | **ETL/Turbo factor** | | **FOV (FHxAP)** | | | | **Matrix** | | | **Slice thickness (mm)** |
| **Siemens 1.5T** | 3D SPACE | 8000 | 335 | 2280 | 242 | | FH: 256  AP: 256 | | | | 256x256 | | | 1 |
| **Siemens 3T** | 3D SPACE | 6000 | 335 | 1800 | 282 | | FH: 256  AP: 256 | | | | 256x256 | | | 1 |
| **GE 1.5T** | 3D CUBE | 6000 | 125 | 1860 | 140 | | FH: 256  AP: 256 | | | | 256x256 | | | 1 |
| **GE 3T** | 3D CUBE | 6000 | 125 | 1860 | 140 | | FH: 256  AP: 256 | | | | 256x256 | | | 1 |
| **Philips 1.5T** | 3D VISTA | 8000 | 355 | 2280 | 110 | | FH: 256  AP: 256 | | | | 256x256 | | | 1 |
| **Philips 3T** | 3D VISTA | 4800 | 378 | 1800 | 202 | | FH: 256  AP: 256 | | | | 256x256 | | | 1 |
| **Toshiba 1.5T** | 3D MVOX | 8000 | 355 | 2280 | 242 | | FH: 256  AP: 256 | | | | 256x256 | | | 1 |
| **3D-Susceptibility Weighted Imaging** | | | | | | | | | | | | | | |
|  | **Sequence type** | **TR (ms)** | **TE (ms)** | **Flip**  **angle °** | **FOV (APxRL)** | | | **Matrix** | | | | **Slice thickness (mm)** | | |
| **Siemens 3T** | 3D FLASH | 46 | TE_1_: 4.9 ms  TE_2_: 13 ms  TE_3_: 20 ms  TE_4_: 27 ms  TE_5_: 34 ms  TE_6_: 41 ms | 20 | AP: 230  RL: 192 | | | 288x288 | | | | 0.8 | | |
| **GE 3T** | 3D SPGR |  |  |  | AP: 230  RL: 192 | | | 288x288 | | | | 0.8 | | |
| **Philips 3T** | 3D FFE with “T1  contrast” enabled |  |  |  | AP: 230  RL: 192 | | | 288x288 | | | | 0.8 | | |
| **Single-voxel proton MRS (1.5 Tesla only)** | | | | | | | | | | | | | | |
|  | | | | Water suppressed | | | | Water unsuppressed | | | | | | |
| **Sequence type (volume selection)** | | | | PRESS | | | | PRESS | | | | | | |
| **TR (ms)** | | | | 3000 | | | | 3000 | | | | | | |
| **TE (ms)** | | | | 30 | | | | 30 | | | | | | |
| **Voxel size (GE, Philips)** | | | | L-R 50 mm  A-P 70 mm  S-I 18 mm | | | | L-R 50 mm  A-P 70 mm  S-I 18 mm | | | | | | |
| **Voxel size (Siemens)** | | | | L-R 40 mm  A-P 40 mm  S-I 20 mm | | | | L-R 40 mm  A-P 40 mm  S-I 20 mm | | | | | | |
| **Spectral bandwidth (Hz)** | | | | 1000 | | | | 1000 | | | | | | |
| **# sample points** | | | | 1024 | | | | 1024 | | | | | | |
| **# averages** | | | | 128 | | | | 16 | | | | | | |
| **Approximate scan time** | | | | 6:24 min | | | | 1 min | | | | | | |

**Propensity matching**

We used propensity-score matching to estimate the effect of clinical phenotype independently among participants with equivalent EDSS and duration. Propensity scores were computed as the predicted probability of phenotype assignment from a logistic regression including EDSS and disease duration as covariates. Participants were then matched using 1:1 nearest-neighbour matching without replacement. To permit flexible yet well-balanced matches, we imposed a 0.2 standard-deviation (SD) calliper on the pooled propensity-score distribution, restricting matches to pairs whose propensity scores differed by no more than 0.2 SD. Compared with stricter callipers (e.g., 0.1), this choice increased the pool of eligible matches and improved retention while maintaining acceptable balance.^1^ Individuals without an eligible match within the calliper were excluded from the matched set. Balance between phenotypes was examined by comparing the distributions of the covariates before and after matching, and all downstream analyses pertaining to phenotype effects were conducted on the matched sample.

**Supplementary Table 2.** LPA model fit indices for model for 1 to 10 profiles.

| **Number of profiles** | **AIC** | **BIC** |
| --- | --- | --- |
| 1 | 367.040 | 456.049 |
| 2 | 236.277 | 418.746 |
| *3 | -34.767 | 241.162 |
| 4 | -91.912 | 277.477 |
| 5 | -174.516 | 288.333 |
| 6 | -228.056 | 328.253 |
| 7 | -246.296 | 403.473 |
| 8 | -257.240 | 485.989 |
| 9 | -231.641 | 605.048 |
| 10 | -276.479 | 653.669 |

*3 profile model was selected as it provided the smallest absolute BIC and AIC values.

**Abbreviations:** AIC=Akaike Information Criterion; BIC=Bayesian Information Criterion.

**Supplementary Figure 1.** Clinical features of the disability profiles.

Motor disability (n=138)

Cognitive disability (n=181)

Global disability (n=261)

**A**

**C**

**D**

**B**

Between-group comparisons of age (Panel A), sex (Panel B), disease duration (Panel C), and clinical phenotypes (Panel D) across the newly defined disability profiles. Statistical analysis from linear regression models for continuous variables and chi-square test for factorial variables.

**Abbreviations:** PPMS=Primary Progressive Multiple Sclerosis; SPMS= Secondary Progressive Multiple Sclerosis; F=females, M=Males. *p* values:*<0.05; ** <0.01; ***<0.001; ****<.001.

**Supplementary Figure 2.** Propensity matching of primary and secondary progressive multiple sclerosis according to Expanded Disability Status Scale and disease duration.

 Distribution of disease duration (Panel A) and Expanded Disability Status Scale (EDSS) (Panel B) in primary progressive (PPMS) and secondary progressive multiple sclerosis (SPMS) patients. Panel C shows standardized mean differences for EDSS and disease duration between PPMS and SPMS before and after propensity-score matching.

**Abbreviations:** EDSS=Expanded Disability Status Scale, PP=primary progressive; SP=secondary progressive; MS=multiple sclerosis.

**Supplementary Figure 3.** Conventional MRI features of the disability profiles.

**C**

**B**

**A**

**E**

**D**

Motor disability (n=138)

Cognitive disability (n=181)

Global disability (n=261)

Between-group comparisons of lesion volume (Panel A), normalized brain volume (Panel B), normalized grey matter volume (Panel C), normalized white matter volume (Panel D), total intracranial volume (Panel E) across the newly defined disability profiles.

Statistical analysis from linear regression models including age, sex, disease duration and scanner model as covariates.

**Abbreviations:** TIV=total intracranial volume.

**Supplementary Table 3.** Summary of regional volume comparisons across the disability profiles.

|  |  | **Cognitive disability *vs* Motor disability** | | | **Global disability *vs* Motor disability** | | | **Cognitive disability *vs* Global disability** | | |
| --- | --- | --- | --- | --- | --- | --- | --- | --- | --- | --- |
|  | **Side** | **β coef** | **SE** | ***FDR-corrected* *p***  **values** | **β coef** | **SE** | ***FDR-corrected* *p***  **values** | **β coef** | **SE** | ***FDR-corrected* *p***  **values** |
| Accumbens Area | R | 0.033 | 0.053 | 0.534 | 0.069 | 0.053 | 0.196 | -0.032 | 0.046 | 0.488 |
|  | L | 0.021 | 0.053 | 0.683 | 0.028 | 0.054 | 0.599 | -0.005 | 0.046 | 0.916 |
| Amygdala | R | 0.024 | 0.052 | 0.638 | -0.037 | 0.053 | 0.479 | 0.059 | 0.045 | 0.192 |
|  | L | 0.014 | 0.052 | 0.792 | -0.021 | 0.053 | 0.688 | 0.034 | 0.046 | 0.461 |
| Pons |  | -0.109 | 0.052 | **0.035** | -0.209 | 0.053 | **<0.001** | 0.086 | 0.045 | 0.057 |
| Brainstem |  | -0.094 | 0.051 | 0.063 | -0.139 | 0.052 | **0.007** | 0.036 | 0.044 | 0.417 |
| Caudate Nucleus | R | -0.167 | 0.050 | **0.001** | -0.139 | 0.051 | **0.006** | -0.037 | 0.044 | 0.394 |
|  | L | -0.177 | 0.051 | **0.001** | -0.118 | 0.052 | **0.025** | -0.067 | 0.045 | 0.136 |
| Cerebellum Exterior | R | -0.086 | 0.051 | 0.092 | -0.144 | 0.052 | **0.006** | 0.049 | 0.044 | 0.276 |
|  | L | -0.076 | 0.050 | 0.133 | -0.149 | 0.051 | **0.004** | 0.064 | 0.044 | 0.149 |
| Hippocampus | R | -0.015 | 0.052 | 0.765 | -0.012 | 0.053 | 0.823 | -0.004 | 0.045 | 0.922 |
|  | L | -0.037 | 0.052 | 0.480 | -0.039 | 0.053 | 0.463 | 0.000 | 0.045 | 0.992 |
| Pallidum | R | -0.164 | 0.051 | **0.001** | -0.152 | 0.052 | **0.004** | -0.023 | 0.045 | 0.613 |
|  | L | -0.141 | **0.050** | 0.005 | -0.189 | 0.051 | **<0.001** | 0.035 | 0.044 | 0.421 |
| Putamen | R | -0.109 | 0.051 | 0.032 | -0.139 | 0.052 | **0.007** | 0.021 | 0.044 | 0.641 |
|  | L | -0.083 | 0.051 | 0.110 | -0.123 | 0.053 | **0.019** | 0.033 | 0.045 | 0.470 |
| Thalamus | R | -0.252 | **0.050** | 0.000 | -0.235 | 0.051 | **<0.001** | -0.033 | 0.044 | 0.456 |
|  | L | -0.231 | 0.051 | 0.000 | -0.223 | 0.052 | **<0.001** | -0.023 | 0.044 | 0.604 |
| Ventral diencephalon | R | -0.215 | 0.051 | 0.000 | -0.185 | 0.052 | **<0.001** | -0.043 | 0.045 | 0.337 |
|  | L | -0.219 | 0.051 | 0.000 | -0.236 | 0.052 | **<0.001** | 0.001 | 0.045 | 0.976 |
| Cerebellar Vermal Lobules I V |  | 0.023 | 0.052 | 0.665 | -0.085 | 0.053 | 0.110 | 0.102 | 0.046 | **0.026** |
| Cerebellar Vermal Lobules VI VII |  | -0.009 | 0.053 | 0.859 | 0.011 | 0.053 | 0.835 | -0.020 | 0.046 | 0.667 |
| Cerebellar Vermal Lobules VIII X |  | 0.087 | 0.053 | 0.097 | 0.031 | 0.053 | 0.560 | 0.058 | 0.046 | 0.204 |
| Basal Forebrain | R | -0.064 | 0.052 | 0.221 | 0.000 | 0.053 | 0.995 | -0.065 | 0.046 | 0.159 |
|  | L | -0.125 | 0.052 | **0.017** | -0.107 | 0.053 | **0.043** | -0.025 | 0.046 | 0.591 |
| Anterior cingulate gyrus | R | -0.049 | 0.052 | 0.350 | -0.027 | 0.053 | 0.613 | -0.024 | 0.045 | 0.602 |
|  | L | -0.037 | 0.052 | 0.475 | -0.054 | 0.053 | 0.310 | 0.013 | 0.045 | 0.773 |
| Anterior insula | R | 0.085 | 0.052 | 0.100 | 0.118 | 0.053 | **0.025** | -0.025 | 0.045 | 0.581 |
|  | L | 0.082 | 0.051 | 0.111 | 0.053 | 0.052 | 0.310 | 0.032 | 0.045 | 0.474 |
| Anterior orbital gyrus | R | -0.045 | 0.049 | 0.365 | -0.113 | 0.050 | **0.025** | 0.061 | 0.043 | 0.162 |
|  | L | -0.085 | 0.050 | 0.091 | -0.111 | 0.051 | **0.030** | 0.019 | 0.044 | 0.665 |
| Angular gyrus | R | 0.031 | 0.052 | 0.542 | -0.013 | 0.053 | 0.809 | 0.043 | 0.045 | 0.338 |
|  | L | 0.011 | 0.052 | 0.829 | -0.038 | 0.053 | 0.467 | 0.047 | 0.045 | 0.300 |
| Calcarine cortex | R | 0.018 | 0.052 | 0.730 | -0.089 | 0.053 | 0.092 | 0.101 | 0.045 | 0.026 |
|  | L | -0.015 | 0.053 | 0.771 | -0.041 | 0.054 | 0.442 | 0.023 | 0.046 | 0.615 |
| Central operculum | R | 0.011 | 0.052 | 0.840 | 0.080 | 0.053 | 0.133 | -0.064 | 0.046 | 0.162 |
|  | L | 0.108 | 0.052 | **0.038** | 0.091 | 0.053 | 0.083 | 0.022 | 0.045 | 0.622 |
| Cuneus | R | 0.016 | 0.051 | 0.754 | -0.086 | 0.052 | 0.102 | 0.096 | 0.045 | **0.033** |
|  | L | -0.081 | 0.051 | 0.114 | -0.027 | 0.053 | 0.605 | -0.056 | 0.045 | 0.216 |
| Entorhinal area | R | -0.009 | 0.053 | 0.857 | -0.026 | 0.053 | 0.620 | 0.015 | 0.046 | 0.739 |
|  | L | -0.061 | 0.052 | 0.241 | -0.124 | 0.053 | **0.020** | 0.054 | 0.046 | 0.235 |
| Frontal operculum | R | -0.064 | 0.051 | 0.216 | 0.011 | 0.052 | 0.839 | -0.074 | 0.045 | 0.102 |
|  | L | -0.020 | 0.051 | 0.692 | 0.015 | 0.052 | 0.774 | -0.034 | 0.045 | 0.446 |
| Frontal pole | R | 0.054 | 0.049 | 0.276 | -0.021 | 0.050 | 0.684 | 0.073 | 0.044 | 0.094 |
|  | L | 0.032 | 0.049 | 0.519 | -0.005 | 0.050 | 0.928 | 0.036 | 0.043 | 0.407 |
| Fusiform gyrus | R | -0.004 | 0.050 | 0.943 | -0.054 | 0.051 | 0.295 | 0.047 | 0.044 | 0.291 |
|  | L | -0.053 | 0.051 | 0.297 | -0.069 | 0.052 | 0.183 | 0.011 | 0.045 | 0.798 |
| Gyrus rectus | R | -0.041 | 0.052 | 0.429 | 0.067 | 0.053 | 0.204 | -0.104 | 0.046 | **0.023** |
|  | L | -0.032 | 0.053 | 0.547 | 0.002 | 0.053 | 0.967 | -0.034 | 0.046 | 0.463 |
| Inferior occipital gyrus | R | 0.085 | 0.052 | 0.104 | 0.054 | 0.053 | 0.306 | 0.034 | 0.045 | 0.452 |
|  | L | 0.068 | 0.052 | 0.191 | 0.102 | 0.053 | 0.055 | -0.027 | 0.046 | 0.550 |
| Inferior temporal gyrus | R | -0.122 | 0.052 | **0.019** | -0.047 | 0.053 | 0.376 | -0.078 | 0.046 | 0.086 |
|  | L | -0.123 | 0.050 | **0.014** | -0.089 | 0.051 | 0.084 | -0.041 | 0.044 | 0.357 |
| Lingual gyrus | R | 0.029 | 0.051 | 0.571 | -0.043 | 0.052 | 0.410 | 0.069 | 0.045 | 0.124 |
|  | L | -0.014 | 0.051 | 0.781 | -0.002 | 0.052 | 0.976 | -0.013 | 0.045 | 0.776 |
| Lateral orbital gyrus | R | -0.081 | 0.052 | 0.122 | -0.024 | 0.053 | 0.649 | -0.059 | 0.046 | 0.203 |
|  | L | -0.008 | 0.052 | 0.873 | -0.004 | 0.053 | 0.934 | -0.004 | 0.046 | 0.926 |
| Middle cingulate gyrus | R | -0.103 | 0.052 | **0.048** | -0.118 | 0.053 | **0.027** | 0.007 | 0.045 | 0.879 |
|  | L | -0.150 | 0.052 | **0.004** | -0.115 | 0.053 | **0.030** | -0.043 | 0.045 | 0.347 |
| Medial frontal cortex | R | -0.036 | 0.052 | 0.485 | -0.028 | 0.053 | 0.596 | -0.010 | 0.045 | 0.822 |
|  | L | -0.012 | 0.052 | 0.813 | -0.038 | 0.053 | 0.477 | 0.023 | 0.046 | 0.617 |
| Middle frontal gyrus | R | 0.013 | 0.052 | 0.795 | -0.067 | 0.053 | 0.208 | 0.076 | 0.045 | 0.096 |
|  | L | 0.009 | 0.052 | 0.856 | -0.050 | 0.053 | 0.343 | 0.056 | 0.045 | 0.216 |
| Middle occipital gyrus | R | 0.096 | 0.051 | 0.060 | -0.015 | 0.052 | 0.780 | 0.110 | 0.045 | **0.015** |
|  | L | 0.011 | 0.052 | 0.835 | -0.026 | 0.053 | 0.623 | 0.035 | 0.046 | 0.440 |
| Medial orbital gyrus | R | -0.064 | 0.051 | 0.211 | -0.009 | 0.052 | 0.858 | -0.056 | 0.045 | 0.218 |
|  | L | -0.041 | 0.052 | 0.429 | 0.015 | 0.053 | 0.777 | -0.055 | 0.046 | 0.227 |
| Postcentral gyrus medial segment | R | 0.057 | 0.052 | 0.276 | 0.042 | 0.053 | 0.430 | 0.018 | 0.046 | 0.698 |
|  | L | 0.041 | 0.053 | 0.431 | -0.032 | 0.054 | 0.550 | 0.071 | 0.046 | 0.122 |
| Precentral gyrus medial segment | R | -0.021 | 0.052 | 0.689 | 0.049 | 0.053 | 0.358 | -0.067 | 0.046 | 0.145 |
|  | L | -0.063 | 0.052 | 0.226 | -0.035 | 0.053 | 0.514 | -0.031 | 0.046 | 0.501 |
| Superior frontal gyrus medial segment | R | -0.086 | 0.050 | 0.087 | -0.114 | 0.051 | **0.026** | 0.021 | 0.044 | 0.640 |
|  | L | -0.080 | 0.050 | 0.109 | -0.167 | 0.051 | **0.001** | 0.076 | 0.043 | 0.079 |
| Middle temporal gyrus | R | -0.116 | 0.050 | **0.021** | -0.149 | 0.051 | **0.004** | 0.022 | 0.044 | 0.611 |
|  | L | -0.110 | 0.051 | **0.031** | -0.126 | 0.052 | **0.016** | 0.007 | 0.045 | 0.867 |
| Occipital pole | R | 0.011 | 0.049 | 0.824 | 0.017 | 0.050 | 0.736 | -0.005 | 0.043 | 0.910 |
|  | L | -0.015 | 0.050 | 0.765 | 0.069 | 0.051 | 0.180 | -0.080 | 0.044 | 0.073 |
| Occipital fusiform gyrus | R | -0.009 | 0.051 | 0.855 | -0.020 | 0.052 | 0.707 | 0.009 | 0.045 | 0.842 |
|  | L | -0.073 | 0.052 | 0.157 | -0.075 | 0.053 | 0.153 | -0.003 | 0.045 | 0.952 |
| Opercular part of the inferior frontal gyrus | R | -0.033 | 0.052 | 0.533 | 0.008 | 0.053 | 0.880 | -0.040 | 0.046 | 0.381 |
|  | L | -0.063 | 0.052 | 0.227 | -0.036 | 0.053 | 0.500 | -0.030 | 0.045 | 0.514 |
| Orbital part of the inferior frontal gyrus | R | -0.094 | 0.051 | 0.069 | -0.062 | 0.052 | 0.236 | -0.036 | 0.045 | 0.423 |
|  | L | -0.077 | 0.052 | 0.141 | -0.070 | 0.053 | 0.188 | -0.012 | 0.046 | 0.800 |
| Posterior cingulate gyrus | R | -0.126 | 0.051 | **0.014** | -0.165 | 0.052 | **0.002** | 0.028 | 0.045 | 0.533 |
|  | L | -0.105 | 0.050 | **0.038** | -0.107 | 0.051 | **0.038** | -0.005 | 0.044 | 0.916 |
| Precuneus | R | -0.079 | 0.051 | 0.125 | -0.186 | 0.052 | **<0.001** | 0.095 | 0.045 | **0.036** |
|  | L | -0.138 | 0.052 | **0.008** | -0.109 | 0.053 | **0.040** | -0.037 | 0.045 | 0.415 |
| Parahippocampal gyrus | R | -0.012 | 0.052 | 0.819 | 0.058 | 0.053 | 0.280 | -0.066 | 0.046 | 0.152 |
|  | L | -0.032 | 0.053 | 0.545 | -0.015 | 0.054 | 0.777 | -0.018 | 0.046 | 0.701 |
| Posterior insula | R | 0.112 | 0.052 | **0.032** | 0.090 | 0.053 | 0.087 | 0.027 | 0.045 | 0.548 |
|  | L | 0.041 | 0.052 | 0.428 | 0.027 | 0.053 | 0.608 | 0.016 | 0.046 | 0.726 |
| Parietal operculum | R | 0.049 | 0.052 | 0.351 | 0.048 | 0.053 | 0.367 | 0.004 | 0.046 | 0.929 |
|  | L | 0.037 | 0.053 | 0.479 | 0.001 | 0.054 | 0.988 | 0.037 | 0.046 | 0.427 |
| Postcentral gyrus | R | -0.003 | 0.052 | 0.958 | -0.054 | 0.053 | 0.312 | 0.048 | 0.046 | 0.299 |
|  | L | 0.056 | 0.052 | 0.283 | 0.000 | 0.053 | 0.995 | 0.057 | 0.046 | 0.218 |
| Posterior orbital gyrus | R | -0.021 | 0.051 | 0.673 | 0.050 | 0.052 | 0.335 | -0.068 | 0.045 | 0.127 |
|  | L | -0.029 | 0.050 | 0.560 | 0.068 | 0.051 | 0.187 | -0.093 | 0.044 | **0.036** |
| Planum polare | R | 0.037 | 0.053 | 0.480 | 0.024 | 0.053 | 0.657 | 0.015 | 0.046 | 0.744 |
|  | L | 0.071 | 0.053 | 0.176 | 0.034 | 0.054 | 0.521 | 0.039 | 0.046 | 0.395 |
| Precentral gyrus | R | -0.006 | 0.052 | 0.902 | -0.019 | 0.053 | 0.727 | 0.011 | 0.046 | 0.811 |
|  | L | -0.047 | 0.052 | 0.361 | -0.027 | 0.053 | 0.614 | -0.022 | 0.045 | 0.620 |
| Planum temporale | R | 0.040 | 0.052 | 0.435 | 0.073 | 0.053 | 0.166 | -0.028 | 0.045 | 0.539 |
|  | L | -0.052 | 0.052 | 0.323 | -0.059 | 0.053 | 0.266 | 0.004 | 0.046 | 0.936 |
| Subcallosal area | R | 0.013 | 0.053 | 0.805 | 0.042 | 0.053 | 0.433 | -0.026 | 0.046 | 0.570 |
|  | L | 0.018 | 0.053 | 0.738 | 0.067 | 0.054 | 0.212 | -0.045 | 0.046 | 0.331 |
| Superior frontal gyrus | R | 0.014 | 0.049 | 0.781 | -0.073 | 0.050 | 0.145 | 0.082 | 0.043 | 0.059 |
|  | L | 0.014 | 0.050 | 0.772 | -0.107 | 0.051 | **0.036** | 0.114 | 0.044 | **0.009** |
| Supplementary motor cortex | R | -0.181 | 0.051 | **<0.001** | -0.167 | 0.052 | **0.001** | -0.025 | 0.045 | 0.579 |
|  | L | -0.140 | 0.051 | **0.007** | -0.185 | 0.052 | **<0.001** | 0.033 | 0.045 | 0.461 |
| Supramarginal gyrus | R | -0.087 | 0.052 | 0.095 | -0.045 | 0.053 | 0.391 | -0.044 | 0.045 | 0.325 |
|  | L | -0.027 | 0.052 | 0.611 | -0.041 | 0.053 | 0.446 | 0.011 | 0.046 | 0.805 |
| Superior occipital gyrus | R | 0.016 | 0.052 | 0.761 | -0.038 | 0.053 | 0.477 | 0.051 | 0.046 | 0.264 |
|  | L | 0.063 | 0.052 | 0.229 | 0.039 | 0.053 | 0.463 | 0.026 | 0.046 | 0.564 |
| Superior parietal lobule | R | 0.025 | 0.051 | 0.624 | -0.018 | 0.053 | 0.739 | 0.042 | 0.045 | 0.358 |
|  | L | -0.101 | 0.051 | **0.050** | -0.121 | 0.052 | **0.022** | 0.012 | 0.045 | 0.797 |
| Superior temporal gyrus | R | -0.096 | 0.051 | 0.060 | -0.019 | 0.052 | 0.714 | -0.078 | 0.045 | 0.080 |
|  | L | -0.114 | 0.050 | **0.024** | -0.143 | 0.051 | **0.006** | 0.019 | 0.044 | 0.665 |
| Temporal pole | R | 0.080 | 0.052 | 0.127 | 0.044 | 0.053 | 0.406 | 0.038 | 0.046 | 0.402 |
|  | L | 0.026 | 0.052 | 0.622 | -0.065 | 0.053 | 0.219 | 0.087 | 0.046 | 0.058 |
| Triangular part of the inferior frontal gyrus | R | 0.004 | 0.052 | 0.935 | -0.041 | 0.053 | 0.435 | 0.043 | 0.046 | 0.347 |
|  | L | -0.034 | 0.052 | 0.518 | -0.032 | 0.053 | 0.542 | -0.003 | 0.046 | 0.939 |
| Transverse temporal gyrus | R | -0.093 | 0.053 | 0.079 | -0.070 | 0.053 | 0.192 | -0.027 | 0.046 | 0.554 |
|  | L | -0.012 | 0.053 | 0.821 | -0.031 | 0.053 | 0.559 | 0.017 | 0.046 | 0.707 |

**Abbreviations**: L = left; R = right, SE = standard error, FDR = False Discovery Rate.

**Supplementary Figure 4.** Regional loadings of independent components (ICs) derived from of grey matter regional volumes (number of patients examined = 580).

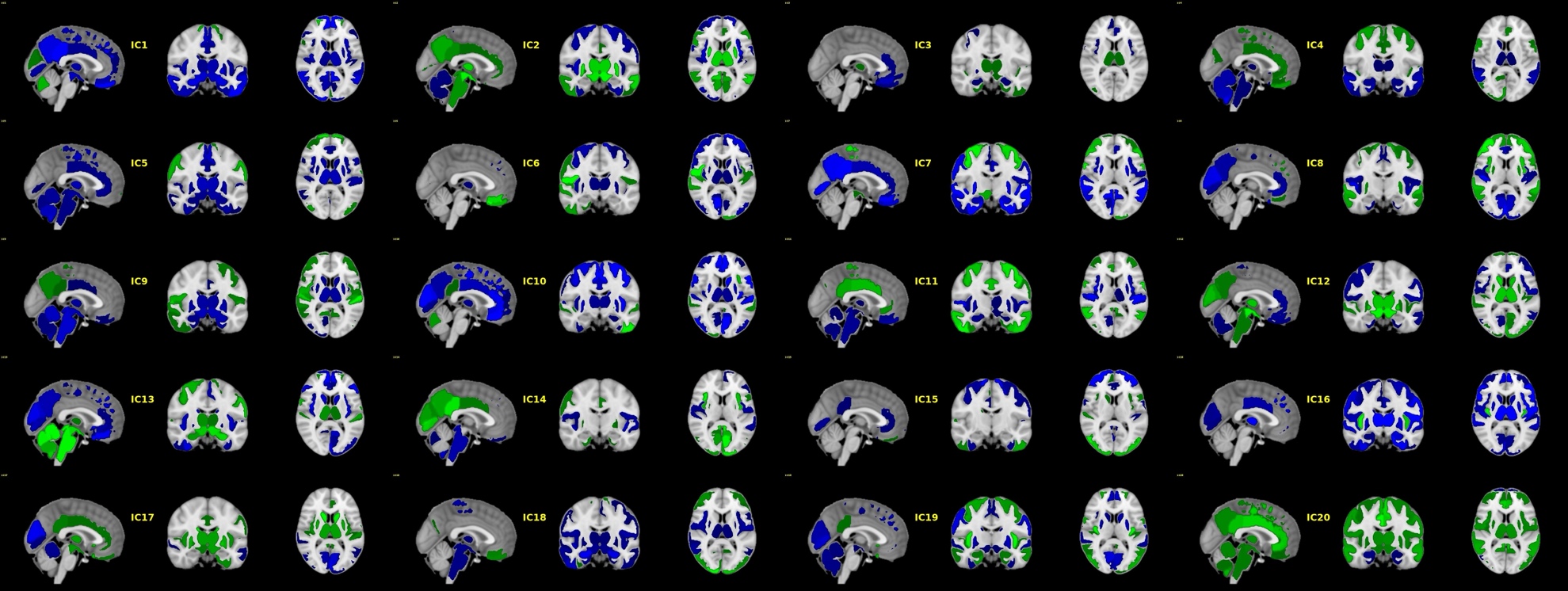


**Abbreviations:** IC=independent component.

**Supplementary Table 4.** Summary of all comparisons of Independent Component loadings for regional volume patterns across the disability profiles.

|  | **Cognitive disability *vs* Motor disability** | | | **Global disability *vs* Motor disability** | | | **Cognitive disability *vs* Global disability** | | |
| --- | --- | --- | --- | --- | --- | --- | --- | --- | --- |
|  | **β coef** | **SE** | ***FDR-corrected* *p***  **values** | **β coef** | **SE** | ***FDR-corrected* *p***  **values** | **β coef** | **SE** | ***FDR-corrected* *p***  **values** |
| **IC 1** | 0.075 | 0.047 | 0.441 | 0.153 | 0.048 | **0.023** | -0.067 | 0.041 | 0.441 |
| **IC 2** | -0.204 | 0.051 | **0.004** | -0.151 | 0.052 | **0.043** | -0.063 | 0.044 | 0.446 |
| **IC 3** | -0.056 | 0.052 | 0.531 | -0.015 | 0.053 | 0.867 | -0.042 | 0.046 | 0.594 |
| **IC 5** | 0.089 | 0.052 | 0.410 | 0.063 | 0.053 | 0.526 | 0.031 | 0.046 | 0.728 |
| **IC 6** | 0.100 | 0.052 | 0.289 | -0.060 | 0.053 | 0.526 | 0.156 | 0.045 | **0.018** |
| **IC 7** | -0.069 | 0.051 | 0.471 | -0.028 | 0.052 | 0.731 | -0.043 | 0.044 | 0.582 |
| **IC 8** | -0.063 | 0.052 | 0.526 | -0.024 | 0.053 | 0.743 | -0.040 | 0.045 | 0.618 |
| **IC 9** | 0.011 | 0.052 | 0.898 | 0.032 | 0.053 | 0.728 | -0.019 | 0.045 | 0.761 |
| **IC 10** | 0.024 | 0.049 | 0.740 | 0.032 | 0.050 | 0.728 | -0.006 | 0.043 | 0.915 |
| **IC 11** | -0.053 | 0.052 | 0.548 | -0.031 | 0.053 | 0.728 | -0.024 | 0.046 | 0.737 |
| **IC 12** | -0.127 | 0.051 | 0.095 | -0.128 | 0.052 | 0.095 | -0.007 | 0.045 | 0.915 |
| **IC 13** | -0.011 | 0.052 | 0.898 | -0.082 | 0.053 | 0.446 | 0.065 | 0.045 | 0.446 |
| **IC 14** | -0.029 | 0.052 | 0.731 | 0.034 | 0.053 | 0.728 | -0.061 | 0.046 | 0.471 |
| **IC 15** | 0.038 | 0.049 | 0.666 | 0.127 | 0.050 | 0.095 | -0.081 | 0.043 | 0.305 |
| **IC 16** | 0.076 | 0.052 | 0.446 | 0.031 | 0.053 | 0.728 | 0.046 | 0.045 | 0.548 |
| **IC 17** | -0.075 | 0.052 | 0.446 | -0.027 | 0.053 | 0.739 | -0.050 | 0.046 | 0.526 |
| **IC 18** | -0.003 | 0.052 | 0.952 | -0.058 | 0.053 | 0.526 | 0.051 | 0.046 | 0.526 |
| **IC 19** | 0.072 | 0.050 | 0.446 | 0.114 | 0.051 | 0.159 | -0.034 | 0.044 | 0.666 |
| **IC 20** | -0.125 | 0.051 | 0.095 | -0.171 | 0.052 | **0.020** | 0.034 | 0.044 | 0.666 |

**Abbreviations**: IC = independent component, SE = standard error, FDR=False Discovery Rate.

**Supplementary Table 5.**  Summary of all regional disconnection comparisons among the disability profiles.

|  |  | **Cognitive disability *vs* Motor disability** | | | **Global disability *vs* Motor disability** | | | **Cognitive disability *vs* Global disability** | | |
| --- | --- | --- | --- | --- | --- | --- | --- | --- | --- | --- |
|  | **Side** | **β coef** | **SE** | ***FDR-corrected* *p***  **values** | **β coef** | **SE** | ***FDR-corrected* *p***  **values** | **β coef** | **SE** | ***FDR-corrected* *p***  **values** |
| Accumbens Area | R | 0.367 | 0.050 | **<0.001** | 0.267 | 0.051 | **<0.001** | 0.118 | 0.044 | 0.998 |
|  | L | 0.331 | 0.051 | **<0.001** | 0.249 | 0.051 | **0.001** | 0.099 | 0.044 | 0.999 |
| Amygdala | R | 0.361 | 0.050 | **<0.001** | 0.273 | 0.051 | **<0.001** | 0.106 | 0.044 | 0.999 |
|  | L | 0.369 | 0.050 | **<0.001** | 0.250 | 0.050 | **<0.001** | 0.136 | 0.043 | 0.637 |
| Pons |  | 0.400 | 0.049 | **<0.001** | 0.253 | 0.050 | **<0.001** | 0.164 | 0.043 | **0.050** |
| Brainstem |  | 0.411 | 0.049 | **<0.001** | 0.259 | 0.050 | **<0.001** | 0.170 | 0.043 | **0.029** |
| Caudate Nucleus | R | 0.280 | 0.051 | **<0.001** | 0.149 | 0.052 | 0.998 | 0.141 | 0.045 | 0.616 |
|  | L | 0.313 | 0.051 | **<0.001** | 0.219 | 0.052 | **0.010** | 0.109 | 0.044 | 1.000 |
| Cerebellum Exterior | R | 0.403 | 0.049 | **<0.001** | 0.264 | 0.050 | **<0.001** | 0.157 | 0.043 | 0.102 |
|  | L | 0.406 | 0.049 | **<0.001** | 0.261 | 0.050 | **<0.001** | 0.162 | 0.043 | 0.060 |
| Hippocampus | R | 0.316 | 0.050 | **<0.001** | 0.248 | 0.051 | **0.001** | 0.084 | 0.044 | 1.000 |
|  | L | 0.400 | 0.049 | **<0.001** | 0.292 | 0.050 | **<0.001** | 0.128 | 0.043 | 1.000 |
| Pallidum | R | 0.397 | 0.049 | **<0.001** | 0.285 | 0.050 | **<0.001** | 0.131 | 0.043 | 0.972 |
|  | L | 0.381 | 0.050 | **<0.001** | 0.257 | 0.051 | **<0.001** | 0.141 | 0.044 | 0.480 |
| Putamen | R | 0.405 | 0.049 | **<0.001** | 0.265 | 0.050 | **<0.001** | 0.158 | 0.043 | 0.107 |
|  | L | 0.402 | 0.049 | **<0.001** | 0.268 | 0.050 | **<0.001** | 0.151 | 0.043 | 0.172 |
| Thalamus | R | 0.406 | 0.049 | **<0.001** | 0.254 | 0.050 | **<0.001** | 0.169 | 0.043 | **0.035** |
|  | L | 0.415 | 0.049 | **<0.001** | 0.272 | 0.050 | **<0.001** | 0.161 | 0.043 | 0.064 |
| Ventral diencephalon | R | 0.389 | 0.049 | **<0.001** | 0.246 | 0.050 | **<0.001** | 0.160 | 0.043 | 0.082 |
|  | L | 0.432 | 0.049 | **<0.001** | 0.288 | 0.049 | **<0.001** | 0.163 | 0.043 | 0.051 |
| Cerebellar Vermal Lobules I V |  | 0.400 | 0.049 | **<0.001** | 0.278 | 0.050 | **<0.001** | 0.141 | 0.043 | 0.412 |
| Cerebellar Vermal Lobules VI VII |  | 0.026 | 0.052 | 1.000 | 0.143 | 0.053 | 1.000 | -0.107 | 0.046 | 1.000 |
| Cerebellar Vermal Lobules VIII X |  | 0.394 | 0.049 | **<0.001** | 0.260 | 0.050 | **<0.001** | 0.151 | 0.043 | 0.187 |
| Basal Forebrain | R | 0.385 | 0.050 | **<0.001** | 0.285 | 0.051 | **<0.001** | 0.119 | 0.044 | 1.000 |
|  | L | 0.387 | 0.050 | **<0.001** | 0.277 | 0.051 | **<0.001** | 0.128 | 0.043 | 1.000 |
| Anterior cingulate gyrus | R | 0.355 | 0.050 | **<0.001** | 0.270 | 0.051 | **<0.001** | 0.103 | 0.044 | 1.000 |
|  | L | 0.368 | 0.050 | **<0.001** | 0.244 | 0.051 | **0.001** | 0.140 | 0.044 | 0.551 |
| Anterior insula | R | 0.355 | 0.050 | **<0.001** | 0.193 | 0.051 | 0.062 | 0.175 | 0.044 | **0.028** |
|  | L | 0.353 | 0.049 | **<0.001** | 0.235 | 0.050 | **0.001** | 0.133 | 0.043 | 0.763 |
| Anterior orbital gyrus | R | 0.331 | 0.050 | **<0.001** | 0.204 | 0.051 | **0.030** | 0.141 | 0.044 | 0.551 |
|  | L | 0.333 | 0.050 | **<0.001** | 0.219 | 0.051 | **0.007** | 0.129 | 0.044 | 1.000 |
| Angular gyrus | R | 0.354 | 0.050 | **<0.001** | 0.228 | 0.051 | **0.003** | 0.142 | 0.044 | 0.444 |
|  | L | 0.340 | 0.050 | **<0.001** | 0.253 | 0.051 | **<0.001** | 0.104 | 0.044 | 1.000 |
| Calcarine cortex | R | 0.293 | 0.051 | **<0.001** | 0.232 | 0.052 | **0.003** | 0.076 | 0.045 | 1.000 |
|  | L | 0.335 | 0.051 | **<0.001** | 0.250 | 0.051 | **0.001** | 0.102 | 0.044 | 1.000 |
| Central operculum | R | 0.277 | 0.051 | **<0.001** | 0.160 | 0.052 | 0.786 | 0.128 | 0.045 | 1.000 |
|  | L | 0.282 | 0.051 | **<0.001** | 0.188 | 0.052 | 0.111 | 0.106 | 0.044 | 1.000 |
| Cuneus | R | 0.368 | 0.050 | **<0.001** | 0.294 | 0.051 | **<0.001** | 0.093 | 0.043 | 1.000 |
|  | L | 0.317 | 0.050 | **<0.001** | 0.262 | 0.051 | **<0.001** | 0.072 | 0.044 | 1.000 |
| Entorhinal area | R | 0.343 | 0.050 | **<0.001** | 0.235 | 0.051 | **0.002** | 0.123 | 0.044 | 1.000 |
|  | L | 0.311 | 0.050 | **<0.001** | 0.261 | 0.051 | **<0.001** | 0.067 | 0.044 | 1.000 |
| Frontal operculum | R | 0.286 | 0.051 | **<0.001** | 0.150 | 0.052 | 1.000 | 0.146 | 0.045 | 0.405 |
|  | L | 0.323 | 0.050 | **<0.001** | 0.218 | 0.051 | **0.008** | 0.120 | 0.044 | 1.000 |
| Frontal pole | R | 0.335 | 0.050 | **<0.001** | 0.211 | 0.051 | **0.016** | 0.138 | 0.044 | 0.663 |
|  | L | 0.366 | 0.050 | **<0.001** | 0.245 | 0.051 | **0.001** | 0.138 | 0.044 | 0.626 |
| Fusiform gyrus | R | 0.345 | 0.050 | **<0.001** | 0.260 | 0.051 | **<0.001** | 0.103 | 0.044 | 1.000 |
|  | L | 0.339 | 0.050 | **<0.001** | 0.304 | 0.051 | **<0.001** | 0.055 | 0.044 | 1.000 |
| Gyrus rectus | R | 0.277 | 0.051 | **<0.001** | 0.169 | 0.052 | 0.464 | 0.119 | 0.045 | 1.000 |
|  | L | 0.282 | 0.051 | **<0.001** | 0.185 | 0.052 | 0.161 | 0.109 | 0.045 | 1.000 |
| Inferior occipital gyrus | R | 0.274 | 0.051 | **<0.001** | 0.174 | 0.052 | 0.294 | 0.112 | 0.044 | 1.000 |
|  | L | 0.306 | 0.050 | **<0.001** | 0.230 | 0.051 | **0.003** | 0.091 | 0.044 | 1.000 |
| Inferior temporal gyrus | R | 0.382 | 0.050 | **<0.001** | 0.267 | 0.050 | **<0.001** | 0.132 | 0.043 | 0.864 |
|  | L | 0.389 | 0.050 | **<0.001** | 0.304 | 0.050 | **<0.001** | 0.105 | 0.043 | 1.000 |
| Lingual gyrus | R | 0.320 | 0.051 | **<0.001** | 0.253 | 0.052 | **<0.001** | 0.084 | 0.044 | 1.000 |
|  | L | 0.289 | 0.051 | **<0.001** | 0.228 | 0.052 | **0.005** | 0.076 | 0.045 | 1.000 |
| Lateral orbital gyrus | R | 0.311 | 0.051 | **<0.001** | 0.183 | 0.052 | 0.163 | 0.141 | 0.044 | 0.603 |
|  | L | 0.313 | 0.050 | **<0.001** | 0.204 | 0.051 | **0.026** | 0.123 | 0.044 | 1.000 |
| Middle cingulate gyrus | R | 0.361 | 0.050 | **<0.001** | 0.255 | 0.051 | **<0.001** | 0.124 | 0.044 | 1.000 |
|  | L | 0.411 | 0.049 | **<0.001** | 0.280 | 0.050 | **<0.001** | 0.149 | 0.043 | 0.216 |
| Medial frontal cortex | R | 0.284 | 0.051 | **<0.001** | 0.234 | 0.052 | **0.003** | 0.066 | 0.045 | 1.000 |
|  | L | 0.121 | 0.052 | 1.000 | 0.104 | 0.053 | 1.000 | 0.024 | 0.046 | 1.000 |
| Middle frontal gyrus | R | 0.375 | 0.050 | **<0.001** | 0.212 | 0.050 | **0.011** | 0.177 | 0.043 | **0.019** |
|  | L | 0.379 | 0.049 | **<0.001** | 0.247 | 0.050 | **<0.001** | 0.149 | 0.043 | 0.233 |
| Middle occipital gyrus | R | 0.353 | 0.050 | **<0.001** | 0.276 | 0.051 | **<0.001** | 0.095 | 0.044 | 1.000 |
|  | L | 0.334 | 0.049 | **<0.001** | 0.222 | 0.050 | **0.005** | 0.127 | 0.043 | 1.000 |
| Medial orbital gyrus | R | 0.287 | 0.051 | **<0.001** | 0.165 | 0.052 | 0.599 | 0.133 | 0.045 | 1.000 |
|  | L | 0.382 | 0.050 | **<0.001** | 0.249 | 0.051 | **<0.001** | 0.149 | 0.044 | 0.244 |
| Postcentral gyrus medial segment | R | 0.323 | 0.050 | **<0.001** | 0.194 | 0.051 | 0.064 | 0.142 | 0.044 | 0.499 |
|  | L | 0.244 | 0.052 | **0.001** | 0.183 | 0.052 | 0.190 | 0.073 | 0.045 | 1.000 |
| Precentral gyrus medial segment | R | 0.262 | 0.051 | **<0.001** | 0.171 | 0.052 | 0.396 | 0.103 | 0.045 | 1.000 |
|  | L | 0.327 | 0.051 | **<0.001** | 0.233 | 0.052 | **0.003** | 0.109 | 0.044 | 1.000 |
| Superior frontal gyrus medial segment | R | 0.364 | 0.050 | **<0.001** | 0.236 | 0.051 | **0.002** | 0.144 | 0.044 | 0.396 |
|  | L | 0.401 | 0.050 | **<0.001** | 0.259 | 0.051 | **<0.001** | 0.159 | 0.044 | 0.105 |
| Middle temporal gyrus | R | 0.345 | 0.050 | **<0.001** | 0.239 | 0.051 | **0.001** | 0.121 | 0.044 | 1.000 |
|  | L | 0.392 | 0.049 | **<0.001** | 0.287 | 0.050 | **<0.001** | 0.123 | 0.043 | 1.000 |
| Occipital pole | R | 0.275 | 0.051 | **<0.001** | 0.219 | 0.052 | **0.010** | 0.070 | 0.045 | 1.000 |
|  | L | 0.323 | 0.050 | **<0.001** | 0.213 | 0.051 | **0.011** | 0.124 | 0.044 | 1.000 |
| Occipital fusiform gyrus | R | 0.238 | 0.051 | **0.002** | 0.171 | 0.052 | 0.426 | 0.078 | 0.045 | 1.000 |
|  | L | 0.312 | 0.050 | **<0.001** | 0.229 | 0.051 | **0.003** | 0.098 | 0.044 | 1.000 |
| Opercular part of the inferior frontal gyrus | R | 0.322 | 0.050 | **<0.001** | 0.170 | 0.051 | 0.348 | 0.164 | 0.044 | 0.075 |
|  | L | 0.299 | 0.050 | **<0.001** | 0.156 | 0.051 | 0.926 | 0.154 | 0.044 | 0.199 |
| Orbital part of the inferior frontal gyrus | R | 0.277 | 0.051 | **<0.001** | 0.160 | 0.052 | 0.834 | 0.128 | 0.045 | 1.000 |
|  | L | 0.331 | 0.050 | **<0.001** | 0.220 | 0.051 | **0.006** | 0.126 | 0.044 | 1.000 |
| Posterior cingulate gyrus | R | 0.348 | 0.050 | **<0.001** | 0.287 | 0.051 | **<0.001** | 0.080 | 0.044 | 1.000 |
|  | L | 0.371 | 0.050 | **<0.001** | 0.278 | 0.051 | **<0.001** | 0.111 | 0.043 | 1.000 |
| Precuneus | R | 0.362 | 0.050 | **<0.001** | 0.292 | 0.051 | **<0.001** | 0.089 | 0.044 | 1.000 |
|  | L | 0.369 | 0.050 | **<0.001** | 0.285 | 0.051 | **<0.001** | 0.103 | 0.043 | 1.000 |
| Parahippocampal gyrus | R | 0.359 | 0.050 | **<0.001** | 0.265 | 0.051 | **<0.001** | 0.112 | 0.044 | 1.000 |
|  | L | 0.397 | 0.048 | **<0.001** | 0.279 | 0.049 | **<0.001** | 0.137 | 0.042 | 0.482 |
| Posterior insula | R | 0.347 | 0.050 | **<0.001** | 0.178 | 0.051 | 0.184 | 0.180 | 0.044 | **0.016** |
|  | L | 0.351 | 0.049 | **<0.001** | 0.222 | 0.050 | **0.004** | 0.144 | 0.043 | 0.325 |
| Parietal operculum | R | 0.307 | 0.051 | **<0.001** | 0.221 | 0.051 | **0.008** | 0.101 | 0.044 | 1.000 |
|  | L | 0.346 | 0.050 | **<0.001** | 0.241 | 0.051 | **0.001** | 0.121 | 0.044 | 1.000 |
| Postcentral gyrus | R | 0.364 | 0.050 | **<0.001** | 0.203 | 0.051 | **0.024** | 0.174 | 0.043 | **0.026** |
|  | L | 0.401 | 0.049 | **<0.001** | 0.271 | 0.050 | **<0.001** | 0.148 | 0.043 | 0.221 |
| Posterior orbital gyrus | R | 0.306 | 0.051 | **<0.001** | 0.202 | 0.052 | **0.041** | 0.117 | 0.045 | 1.000 |
|  | L | 0.288 | 0.050 | **<0.001** | 0.184 | 0.051 | 0.122 | 0.116 | 0.044 | 1.000 |
| Planum polare | R | 0.379 | 0.050 | **<0.001** | 0.219 | 0.050 | **0.006** | 0.174 | 0.043 | **0.025** |
|  | L | 0.360 | 0.049 | **<0.001** | 0.231 | 0.050 | **0.002** | 0.145 | 0.043 | 0.317 |
| Precentral gyrus | R | 0.376 | 0.050 | **<0.001** | 0.224 | 0.050 | **0.004** | 0.167 | 0.043 | **0.045** |
|  | L | 0.386 | 0.049 | **<0.001** | 0.253 | 0.050 | **<0.001** | 0.150 | 0.043 | 0.212 |
| Planum temporale | R | 0.304 | 0.051 | **<0.001** | 0.154 | 0.052 | 1.000 | 0.160 | 0.044 | 0.124 |
|  | L | 0.333 | 0.050 | **<0.001** | 0.221 | 0.051 | **0.006** | 0.126 | 0.044 | 1.000 |
| Subcallosal area | R | 0.315 | 0.051 | **<0.001** | 0.235 | 0.052 | **0.003** | 0.096 | 0.044 | 1.000 |
|  | L | 0.272 | 0.051 | **<0.001** | 0.179 | 0.052 | 0.242 | 0.105 | 0.045 | 1.000 |
| Superior frontal gyrus | R | 0.389 | 0.050 | **<0.001** | 0.230 | 0.050 | **0.002** | 0.174 | 0.043 | **0.025** |
|  | L | 0.404 | 0.049 | **<0.001** | 0.268 | 0.050 | **<0.001** | 0.154 | 0.043 | 0.141 |
| Supplementary motor cortex | R | 0.325 | 0.050 | **<0.001** | 0.207 | 0.051 | **0.022** | 0.132 | 0.044 | 0.985 |
|  | L | 0.370 | 0.050 | **<0.001** | 0.240 | 0.051 | **0.001** | 0.145 | 0.044 | 0.333 |
| Supramarginal gyrus | R | 0.368 | 0.050 | **<0.001** | 0.242 | 0.051 | **0.001** | 0.142 | 0.043 | 0.423 |
|  | L | 0.329 | 0.050 | **<0.001** | 0.255 | 0.051 | **<0.001** | 0.091 | 0.044 | 1.000 |
| Superior occipital gyrus | R | 0.367 | 0.050 | **<0.001** | 0.271 | 0.051 | **<0.001** | 0.114 | 0.044 | 1.000 |
|  | L | 0.353 | 0.049 | **<0.001** | 0.245 | 0.050 | **0.001** | 0.124 | 0.043 | 1.000 |
| Superior parietal lobule | R | 0.403 | 0.049 | **<0.001** | 0.268 | 0.050 | **<0.001** | 0.152 | 0.043 | 0.155 |
|  | L | 0.399 | 0.049 | **<0.001** | 0.261 | 0.050 | **<0.001** | 0.155 | 0.043 | 0.107 |
| Superior temporal gyrus | R | 0.338 | 0.050 | **<0.001** | 0.227 | 0.051 | **0.004** | 0.126 | 0.044 | 1.000 |
|  | L | 0.349 | 0.050 | **<0.001** | 0.225 | 0.051 | **0.004** | 0.139 | 0.043 | 0.545 |
| Temporal pole | R | 0.380 | 0.050 | **<0.001** | 0.275 | 0.051 | **<0.001** | 0.124 | 0.043 | 1.000 |
|  | L | 0.346 | 0.049 | **<0.001** | 0.191 | 0.050 | 0.057 | 0.167 | 0.043 | **0.045** |
| Triangular part of the inferior frontal gyrus | R | 0.309 | 0.051 | **<0.001** | 0.179 | 0.052 | 0.204 | 0.142 | 0.044 | 0.555 |
|  | L | 0.349 | 0.050 | **<0.001** | 0.233 | 0.051 | **0.002** | 0.132 | 0.044 | 0.961 |
| Transverse temporal gyrus | R | 0.336 | 0.050 | **<0.001** | 0.178 | 0.051 | 0.199 | 0.169 | 0.044 | **0.049** |
|  | L | 0.367 | 0.050 | **<0.001** | 0.188 | 0.051 | 0.097 | 0.118 | 0.044 | 1.000 |

**Abbreviations**: L= left, R = right, SE = standard error, FDR = False Discovery Rate.

**Supplementary Figure 5.** Regional loadings of independent components (IC) derived from of grey matter regional disconnection (number of patients examined = 580).

**
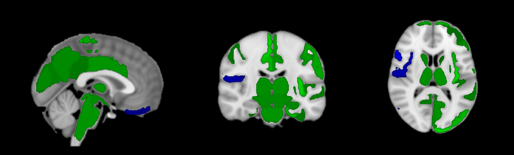

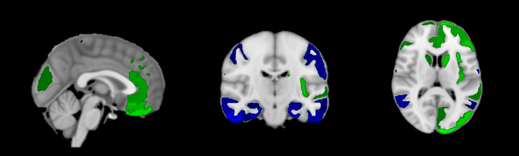

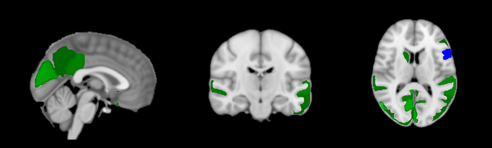

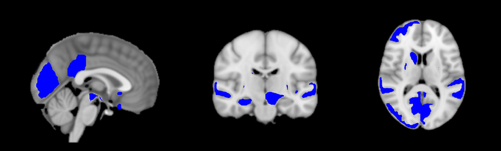

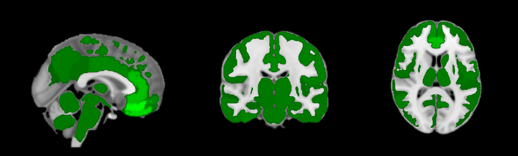

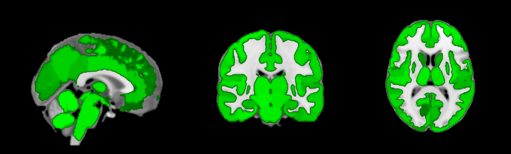

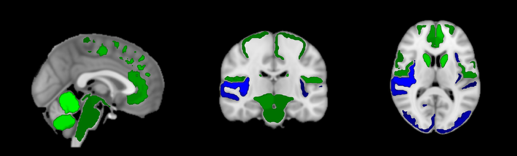
**
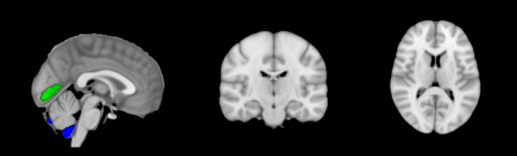
**
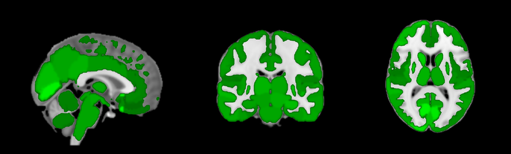

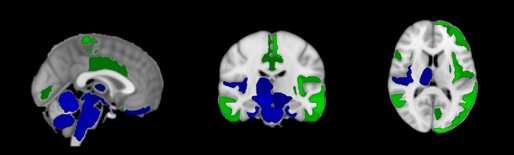

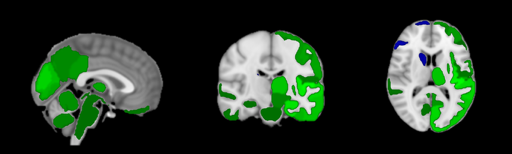

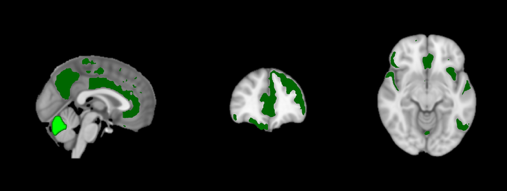
**
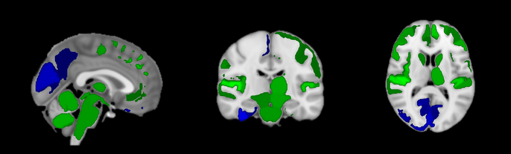

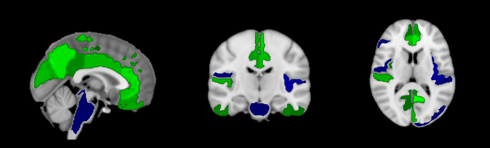

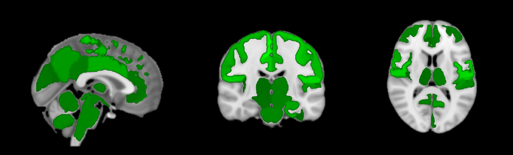

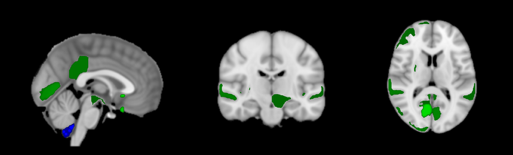

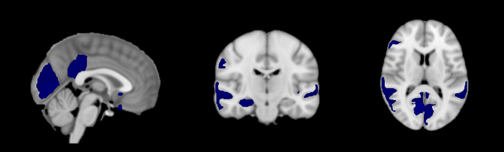

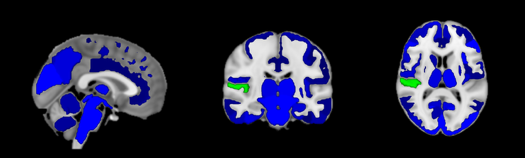

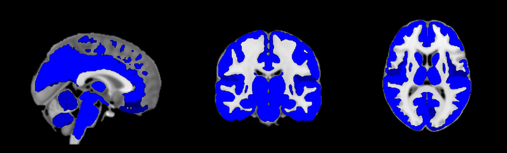

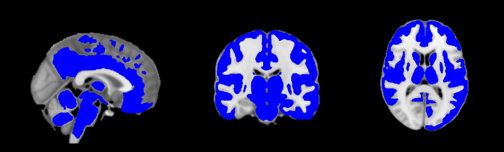

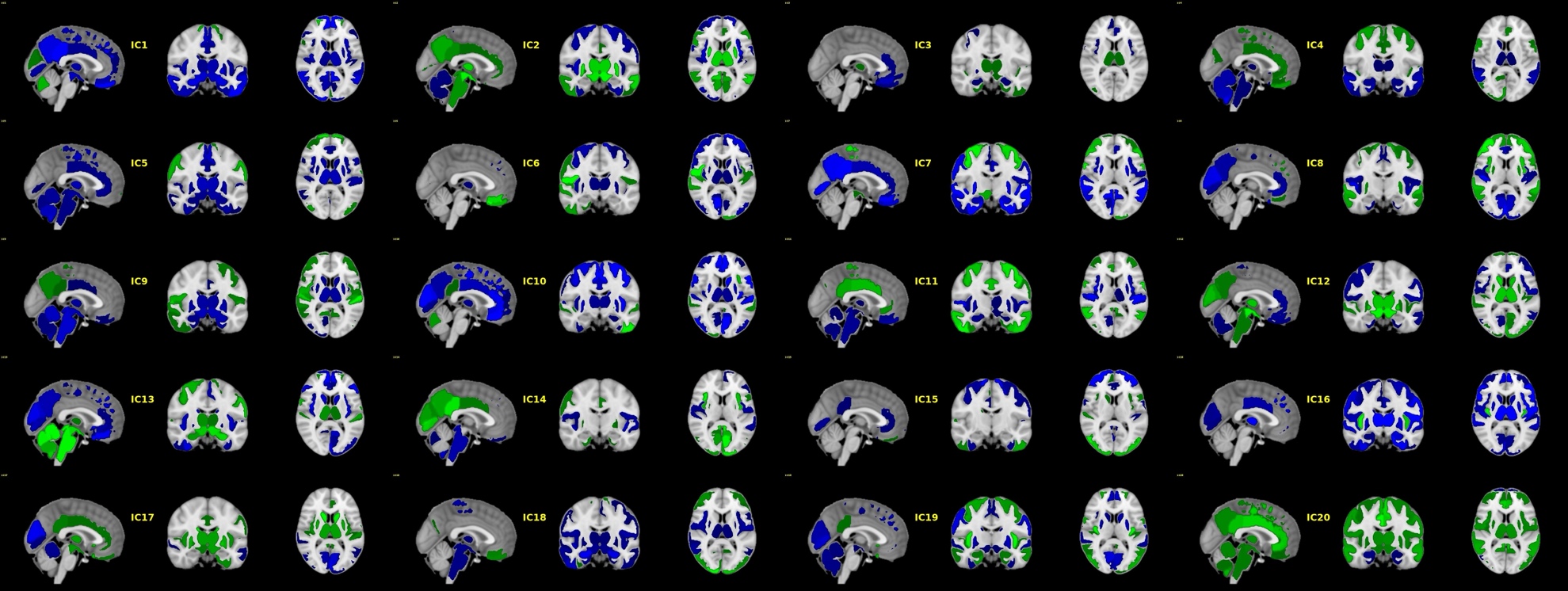


IC18

IC16

IC20

IC19

IC17

IC13

IC15

IC14

IC12

IC11

IC10

IC9

IC7

IC8

IC5

IC6

IC4

IC3

IC2

IC1

**Abbreviations:** IC=independent component.

**Supplementary Table 6.** Summary of Independent Component loading comparisons for regional disconnection patterns across the disability phenotypes.

|  | **Cognitive disability *vs***  **Motor disability** | | | **Global disability *vs* Motor disability** | | | **Cognitive disability *vs* Global disability** | | |
| --- | --- | --- | --- | --- | --- | --- | --- | --- | --- |
|  | **β coef** | **SE** | ***FDR-corrected***  ***p***  **values** | **β coef** | **SE** | ***FDR-corrected***  ***p***  **values** | **β coef** | **SE** | ***FDR-corrected* *p***  **values** |
| **IC 1** | -0.094 | 0.052 | 0.207 | 0.038 | 0.053 | 0.735 | -0.130 | 0.045 | **0.033** |
| **IC 2** | -0.079 | 0.052 | 0.355 | -0.088 | 0.053 | 0.279 | 0.003 | 0.046 | 0.979 |
| **IC 3** | 0.054 | 0.053 | 0.560 | -0.001 | 0.053 | 0.990 | 0.055 | 0.046 | 0.492 |
| **IC 4** | -0.073 | 0.053 | 0.413 | -0.066 | 0.054 | 0.492 | -0.012 | 0.046 | 0.960 |
| **IC 5** | 0.093 | 0.051 | 0.207 | 0.108 | 0.052 | 0.147 | -0.008 | 0.044 | 0.978 |
| **IC 6** | 0.062 | 0.052 | 0.492 | -0.031 | 0.053 | 0.817 | 0.091 | 0.046 | 0.167 |
| **IC 7** | 0.006 | 0.053 | 0.978 | -0.047 | 0.053 | 0.661 | 0.049 | 0.046 | 0.560 |
| **IC 8** | -0.014 | 0.053 | 0.960 | -0.034 | 0.054 | 0.798 | 0.018 | 0.046 | 0.927 |
| **IC 9** | 0.147 | 0.051 | 0.033 | 0.176 | 0.052 | **0.013** | -0.017 | 0.045 | 0.927 |
| **IC 10** | -0.001 | 0.052 | 0.990 | 0.126 | 0.053 | 0.099 | -0.118 | 0.046 | 0.059 |
| **IC 11** | -0.095 | 0.052 | 0.207 | -0.044 | 0.053 | 0.666 | -0.055 | 0.046 | 0.492 |
| **IC 12** | 0.200 | 0.052 | **0.004** | 0.117 | 0.053 | 0.136 | 0.090 | 0.045 | 0.167 |
| **IC 13** | 0.299 | 0.050 | **0.000** | 0.174 | 0.051 | **0.013** | 0.136 | 0.044 | **0.026** |
| **IC 14** | 0.028 | 0.053 | 0.859 | -0.012 | 0.054 | 0.960 | 0.039 | 0.046 | 0.661 |
| **IC 15** | 0.033 | 0.052 | 0.798 | 0.055 | 0.053 | 0.560 | -0.018 | 0.046 | 0.927 |
| **IC 16** | -0.074 | 0.053 | 0.413 | -0.073 | 0.054 | 0.416 | -0.006 | 0.046 | 0.978 |
| **IC 17** | -0.075 | 0.052 | 0.446 | 0.146 | 0.053 | **0.041** | 0.017 | 0.046 | 0.927 |
| **IC 18** | -0.003 | 0.052 | 0.952 | 0.056 | 0.053 | 0.560 | -0.039 | 0.046 | 0.661 |
| **IC 19** | 0.072 | 0.050 | 0.446 | -0.008 | 0.053 | 0.978 | 0.016 | 0.046 | 0.932 |
| **IC 20** | -0.125 | 0.051 | 0.095 | 0.115 | 0.053 | 0.143 | 0.003 | 0.046 | 0.979 |

**Abbreviations**: IC = independent component, SE = standard error, FDR = False Discovery Rate.

**Sensitivity analysis: (Latent Profile Analysis) LPA Profiles in PP and SPMS**

We re-run LPA in PP and SPMS separately, identifying similar profiles as shown in **Supplementary Figure 6.**

**Supplementary Figure 6.** Clinical scores across disability profiles in PP and SPMS

(n=122)

(n=88)

(n=67)

**A**

(n=137)

(n=95)

(n=71)

**B**

Clinical scores across disability profiles identified by latent profile analysis, performed separately in primary progressive (Panel A) and secondary progressive (Panel B) multiple sclerosis patients.

**Abbreviations:** PP=primary progressive; SP=secondary progressive; MS=multiple sclerosis; EDSS=Expanded Disability Status Scale, 9HPT=9-Hole Peg Test, 25FWT= timed 25-Foot Walking Test, SDMT=Symbol Digit Modalities Test, BVMT= Brief Visuospatial Memory Test Revised.

**Sensitivity analysis: Clinical phenotype influence on MRI measures among the disability profiles.**

For lesion and whole brain volumes, disability profile × clinical phenotype interaction terms were small in magnitude and non-significant. For global *vs* motor disability, interaction effects were close to zero for normalised brain, grey matter and white matter volumes (Lesion volume: β = 0.470, SE = 0.211, *p* = 0.26; normalised brain volume: β = 0.017, SE = 0.202, FDR-corrected *p* = 0.93; normalised grey matter volume: β = 0.051, SE = 0.198, FDR-corrected *p* = 0.80; normalised white matter volume: β = −0.010, SE = 0.204, FDR-corrected *p* = 0.96). Similarly, for cognitive versus motor disability, interaction terms for normalised brain, grey matter and white matter volumes remained very small and non-significant (lesion volume: β = 0.360, SE = 0.224, FDR-corrected *p* = 0.36; normalised brain volume: β = 0.076, SE = 0.214, FDR-corrected *p* = 0.72; normalised grey matter volume: β = 0.004, SE = 0.210, FDR-corrected *p* = 0.99; normalised white matter volume: β = 0.118, SE = 0.217, FDR-corrected *p* = 0.99), indicating no evidence that between-profile differences in global brain volume and lesion burden varied by SPMS versus PPMS phenotype.

Finally, interaction terms for the whole-brain disconnection index were modest and non-significant (global *vs* motor Disability: β = 0.373, SE = 0.202, FDR-corrected *p* = 0.33; cognitive *vs* motor disability: β = 0.165, SE = 0.214, FDR-corrected *p* = 0.99), again providing no evidence that profile-related differences in overall disconnection varied by SPMS versus PPMS.

Across all regional volumes and disconnection and their network-level components, disability profile × phenotype (SPMS *vs* PPMS) interaction terms were small in magnitude, and none survived FDR correction (**Supplementary Tables 7-10**).

**Supplementary Table 7.** Summarizes disability profile × clinical phenotype (SPMS vs PPMS) interaction terms for regional volumetric measures.

| **MRI metric** | **Side** | **Profile** | **Phenotype** | **β coef** | **SE** | **FDR-corrected p- values** |
| --- | --- | --- | --- | --- | --- | --- |
| Accumbens | R | Global disability vs Motor disability | SPMS vs PPMS | 0.048 | 0.218 | 0.997 |
|  | L | Global disability vs Motor disability | SPMS vs PPMS | -0.124 | 0.216 | 0.997 |
| Amygdala | R | Global disability vs Motor disability | SPMS vs PPMS | 0.147 | 0.217 | 0.997 |
|  | L | Global disability vs Motor disability | SPMS vs PPMS | 0.201 | 0.217 | 0.997 |
| Pons |  | Global disability vs Motor disability | SPMS vs PPMS | -0.130 | 0.211 | 0.997 |
| Brain Stem |  | Global disability vs Motor disability | SPMS vs PPMS | 0.017 | 0.212 | 0.997 |
| Caudate Nucleus | R | Global disability vs Motor disability | SPMS vs PPMS | 0.275 | 0.203 | 0.997 |
|  | L | Global disability vs Motor disability | SPMS vs PPMS | 0.184 | 0.211 | 0.997 |
| Cerebellum Exterior | R | Global disability vs Motor disability | SPMS vs PPMS | 0.016 | 0.213 | 0.997 |
|  | L | Global disability vs Motor disability | SPMS vs PPMS | 0.113 | 0.21 | 0.997 |
| Hippocampus | R | Global disability vs Motor disability | SPMS vs PPMS | 0.129 | 0.212 | 0.997 |
|  | L | Global disability vs Motor disability | SPMS vs PPMS | 0.050 | 0.217 | 0.997 |
| Pallidum | R | Global disability vs Motor disability | SPMS vs PPMS | -0.182 | 0.211 | 0.997 |
|  | L | Global disability vs Motor disability | SPMS vs PPMS | 0.019 | 0.199 | 0.997 |
| Putamen | R | Global disability vs Motor disability | SPMS vs PPMS | -0.095 | 0.207 | 0.997 |
|  | L | Global disability vs Motor disability | SPMS vs PPMS | -0.099 | 0.21 | 0.997 |
| Thalamus Proper | R | Global disability vs Motor disability | SPMS vs PPMS | -0.161 | 0.212 | 0.997 |
|  | L | Global disability vs Motor disability | SPMS vs PPMS | -0.372 | 0.212 | 0.997 |
| Ventral DC | R | Global disability vs Motor disability | SPMS vs PPMS | -0.111 | 0.213 | 0.997 |
|  | L | Global disability vs Motor disability | SPMS vs PPMS | -0.234 | 0.214 | 0.997 |
| Cerebellar Vermal Lobules I V |  | Global disability vs Motor disability | SPMS vs PPMS | 0.028 | 0.214 | 0.997 |
| Cerebellar Vermal Lobules VI VII |  | Global disability vs Motor disability | SPMS vs PPMS | 0.214 | 0.219 | 0.997 |
| Cerebellar Vermal Lobules VIII X |  | Global disability vs Motor disability | SPMS vs PPMS | 0.054 | 0.216 | 0.997 |
| Basal Forebrain | L | Global disability vs Motor disability | SPMS vs PPMS | -0.090 | 0.217 | 0.997 |
|  | R | Global disability vs Motor disability | SPMS vs PPMS | -0.380 | 0.215 | 0.997 |
| Anterior cingulate gyrus | R | Global disability vs Motor disability | SPMS vs PPMS | -0.454 | 0.214 | 0.997 |
|  | L | Global disability vs Motor disability | SPMS vs PPMS | -0.203 | 0.213 | 0.997 |
| Anterior insula | R | Global disability vs Motor disability | SPMS vs PPMS | 0.272 | 0.213 | 0.997 |
|  | L | Global disability vs Motor disability | SPMS vs PPMS | 0.302 | 0.209 | 0.997 |
| Anterior orbital gyrus | R | Global disability vs Motor disability | SPMS vs PPMS | 0.161 | 0.206 | 0.997 |
|  | L | Global disability vs Motor disability | SPMS vs PPMS | -0.074 | 0.209 | 0.997 |
| Angular gyrus | R | Global disability vs Motor disability | SPMS vs PPMS | -0.285 | 0.214 | 0.997 |
|  | L | Global disability vs Motor disability | SPMS vs PPMS | 0.176 | 0.216 | 0.997 |
| Calcarine cortex | R | Global disability vs Motor disability | SPMS vs PPMS | 0.062 | 0.214 | 0.997 |
|  | L | Global disability vs Motor disability | SPMS vs PPMS | -0.114 | 0.217 | 0.997 |
| Central operculum | R | Global disability vs Motor disability | SPMS vs PPMS | -0.220 | 0.214 | 0.997 |
|  | L | Global disability vs Motor disability | SPMS vs PPMS | -0.165 | 0.213 | 0.997 |
| Cuneus | R | Global disability vs Motor disability | SPMS vs PPMS | 0.121 | 0.214 | 0.997 |
|  | L | Global disability vs Motor disability | SPMS vs PPMS | -0.155 | 0.214 | 0.997 |
| Entorhinal area | R | Global disability vs Motor disability | SPMS vs PPMS | 0.090 | 0.216 | 0.997 |
|  | L | Global disability vs Motor disability | SPMS vs PPMS | -0.026 | 0.217 | 0.997 |
| Frontal operculum | R | Global disability vs Motor disability | SPMS vs PPMS | 0.022 | 0.213 | 0.997 |
|  | L | Global disability vs Motor disability | SPMS vs PPMS | 0.305 | 0.211 | 0.997 |
| Frontal pole | R | Global disability vs Motor disability | SPMS vs PPMS | 0.181 | 0.200 | 0.997 |
|  | L | Global disability vs Motor disability | SPMS vs PPMS | 0.106 | 0.202 | 0.997 |
| Fusiform gyrus | R | Global disability vs Motor disability | SPMS vs PPMS | -0.151 | 0.21 | 0.997 |
|  | L | Global disability vs Motor disability | SPMS vs PPMS | -0.147 | 0.214 | 0.997 |
| Gyrus rectus | R | Global disability vs Motor disability | SPMS vs PPMS | -0.001 | 0.215 | 0.998 |
|  | L | Global disability vs Motor disability | SPMS vs PPMS | -0.050 | 0.215 | 0.997 |
| Inferior occipital gyrus | R | Global disability vs Motor disability | SPMS vs PPMS | 0.160 | 0.219 | 0.997 |
|  | L | Global disability vs Motor disability | SPMS vs PPMS | -0.054 | 0.218 | 0.997 |
| Inferior temporal gyrus | R | Global disability vs Motor disability | SPMS vs PPMS | -0.368 | 0.214 | 0.997 |
|  | L | Global disability vs Motor disability | SPMS vs PPMS | -0.074 | 0.208 | 0.997 |
| Lingual gyrus | R | Global disability vs Motor disability | SPMS vs PPMS | 0.362 | 0.211 | 0.997 |
|  | L | Global disability vs Motor disability | SPMS vs PPMS | -0.004 | 0.209 | 0.997 |
| Lateral orbital gyrus | R | Global disability vs Motor disability | SPMS vs PPMS | -0.203 | 0.219 | 0.997 |
|  | L | Global disability vs Motor disability | SPMS vs PPMS | -0.259 | 0.218 | 0.997 |
| Middle cingulate gyrus | R | Global disability vs Motor disability | SPMS vs PPMS | -0.215 | 0.216 | 0.997 |
|  | L | Global disability vs Motor disability | SPMS vs PPMS | -0.081 | 0.213 | 0.997 |
| Medial frontal cortex | R | Global disability vs Motor disability | SPMS vs PPMS | 0.176 | 0.209 | 0.997 |
|  | L | Global disability vs Motor disability | SPMS vs PPMS | 0.194 | 0.214 | 0.997 |
| Middle frontal gyrus | R | Global disability vs Motor disability | SPMS vs PPMS | -0.004 | 0.215 | 0.997 |
|  | L | Global disability vs Motor disability | SPMS vs PPMS | 0.174 | 0.214 | 0.997 |
| Middle occipital gyrus | R | Global disability vs Motor disability | SPMS vs PPMS | 0.208 | 0.213 | 0.997 |
|  | L | Global disability vs Motor disability | SPMS vs PPMS | 0.313 | 0.214 | 0.997 |
| Medial orbital gyrus | R | Global disability vs Motor disability | SPMS vs PPMS | -0.136 | 0.215 | 0.997 |
|  | L | Global disability vs Motor disability | SPMS vs PPMS | -0.152 | 0.217 | 0.997 |
| Postcentral gyrus medial segment | R | Global disability vs Motor disability | SPMS vs PPMS | 0.010 | 0.218 | 0.997 |
|  | L | Global disability vs Motor disability | SPMS vs PPMS | -0.073 | 0.216 | 0.997 |
| Precentral gyrus medial segment | R | Global disability vs Motor disability | SPMS vs PPMS | 0.075 | 0.22 | 0.997 |
|  | L | Global disability vs Motor disability | SPMS vs PPMS | 0.048 | 0.217 | 0.997 |
| Superior frontal gyrus medial segment | R | Global disability vs Motor disability | SPMS vs PPMS | 0.275 | 0.208 | 0.997 |
|  | L | Global disability vs Motor disability | SPMS vs PPMS | 0.258 | 0.207 | 0.997 |
| Middle temporal gyrus | R | Global disability vs Motor disability | SPMS vs PPMS | -0.300 | 0.206 | 0.997 |
|  | L | Global disability vs Motor disability | SPMS vs PPMS | 0.119 | 0.212 | 0.997 |
| Occipital pole | R | Global disability vs Motor disability | SPMS vs PPMS | 0.164 | 0.203 | 0.997 |
|  | L | Global disability vs Motor disability | SPMS vs PPMS | -0.182 | 0.207 | 0.997 |
| Occipital fusiform gyrus | R | Global disability vs Motor disability | SPMS vs PPMS | 0.100 | 0.215 | 0.997 |
|  | L | Global disability vs Motor disability | SPMS vs PPMS | 0.390 | 0.212 | 0.997 |
| Opercular part of the inferior frontal gyrus | R | Global disability vs Motor disability | SPMS vs PPMS | -0.101 | 0.219 | 0.997 |
|  | L | Global disability vs Motor disability | SPMS vs PPMS | 0.193 | 0.215 | 0.997 |
| Orbital part of the inferior frontal gyrus | R | Global disability vs Motor disability | SPMS vs PPMS | 0.096 | 0.211 | 0.997 |
|  | L | Global disability vs Motor disability | SPMS vs PPMS | -0.006 | 0.217 | 0.997 |
| Posterior cingulate gyrus | R | Global disability vs Motor disability | SPMS vs PPMS | -0.079 | 0.212 | 0.997 |
|  | L | Global disability vs Motor disability | SPMS vs PPMS | -0.183 | 0.21 | 0.997 |
| Precuneus | R | Global disability vs Motor disability | SPMS vs PPMS | 0.055 | 0.212 | 0.997 |
|  | L | Global disability vs Motor disability | SPMS vs PPMS | 0.163 | 0.214 | 0.997 |
| Parahippocampal gyrus | R | Global disability vs Motor disability | SPMS vs PPMS | 0.400 | 0.216 | 0.997 |
|  | L | Global disability vs Motor disability | SPMS vs PPMS | 0.052 | 0.217 | 0.997 |
| Posterior insula | R | Global disability vs Motor disability | SPMS vs PPMS | 0.008 | 0.213 | 0.997 |
|  | L | Global disability vs Motor disability | SPMS vs PPMS | -0.041 | 0.214 | 0.997 |
| Parietal operculum | R | Global disability vs Motor disability | SPMS vs PPMS | -0.014 | 0.214 | 0.997 |
|  | L | Global disability vs Motor disability | SPMS vs PPMS | 0.033 | 0.216 | 0.997 |
| Postcentral gyrus | R | Global disability vs Motor disability | SPMS vs PPMS | 0.180 | 0.214 | 0.997 |
|  | L | Global disability vs Motor disability | SPMS vs PPMS | -0.299 | 0.217 | 0.997 |
| Posterior orbital gyrus | R | Global disability vs Motor disability | SPMS vs PPMS | 0.012 | 0.21 | 0.997 |
|  | L | Global disability vs Motor disability | SPMS vs PPMS | -0.136 | 0.209 | 0.997 |
| Planum polare | R | Global disability vs Motor disability | SPMS vs PPMS | -0.085 | 0.217 | 0.997 |
|  | L | Global disability vs Motor disability | SPMS vs PPMS | 0.013 | 0.217 | 0.997 |
| Precentral gyrus | R | Global disability vs Motor disability | SPMS vs PPMS | -0.237 | 0.214 | 0.997 |
|  | L | Global disability vs Motor disability | SPMS vs PPMS | -0.209 | 0.215 | 0.997 |
| Planum temporale | R | Global disability vs Motor disability | SPMS vs PPMS | -0.372 | 0.211 | 0.997 |
|  | L | Global disability vs Motor disability | SPMS vs PPMS | 0.372 | 0.215 | 0.997 |
| Subcallosal area | R | Global disability vs Motor disability | SPMS vs PPMS | 0.159 | 0.218 | 0.997 |
|  | L | Global disability vs Motor disability | SPMS vs PPMS | -0.008 | 0.219 | 0.997 |
| Superior frontal gyrus | R | Global disability vs Motor disability | SPMS vs PPMS | 0.167 | 0.202 | 0.997 |
|  | L | Global disability vs Motor disability | SPMS vs PPMS | 0.022 | 0.205 | 0.997 |
| Supplementary motor cortex | R | Global disability vs Motor disability | SPMS vs PPMS | 0.092 | 0.213 | 0.997 |
|  | L | Global disability vs Motor disability | SPMS vs PPMS | 0.124 | 0.212 | 0.997 |
| Supramarginal gyrus | R | Global disability vs Motor disability | SPMS vs PPMS | -0.057 | 0.214 | 0.997 |
|  | L | Global disability vs Motor disability | SPMS vs PPMS | 0.032 | 0.216 | 0.997 |
| Superior occipital gyrus | R | Global disability vs Motor disability | SPMS vs PPMS | -0.146 | 0.216 | 0.997 |
|  | L | Global disability vs Motor disability | SPMS vs PPMS | -0.157 | 0.219 | 0.997 |
| Superior parietal lobule | R | Global disability vs Motor disability | SPMS vs PPMS | 0.068 | 0.213 | 0.997 |
|  | L | Global disability vs Motor disability | SPMS vs PPMS | 0.122 | 0.213 | 0.997 |
| Superior temporal gyrus | R | Global disability vs Motor disability | SPMS vs PPMS | -0.326 | 0.21 | 0.997 |
|  | L | Global disability vs Motor disability | SPMS vs PPMS | 0.093 | 0.209 | 0.997 |
| Temporal pole | R | Global disability vs Motor disability | SPMS vs PPMS | 0.504 | 0.217 | 0.997 |
|  | L | Global disability vs Motor disability | SPMS vs PPMS | 0.461 | 0.216 | 0.997 |
| Triangular part of the inferior frontal gyrus | R | Global disability vs Motor disability | SPMS vs PPMS | -0.041 | 0.215 | 0.997 |
|  | L | Global disability vs Motor disability | SPMS vs PPMS | 0.049 | 0.214 | 0.997 |
| Transverse temporal gyrus | R | Global disability vs Motor disability | SPMS vs PPMS | 0.024 | 0.22 | 0.997 |
|  | L | Global disability vs Motor disability | SPMS vs PPMS | -0.043 | 0.217 | 0.997 |
| Accumbens Area | R | Cognitive disability vs Motor disability | SPMS vs PPMS | -0.008 | 0.232 | 0.997 |
|  | L | Cognitive disability vs Motor disability | SPMS vs PPMS | -0.242 | 0.229 | 0.997 |
| Amygdala | R | Cognitive disability vs Motor disability | SPMS vs PPMS | -0.021 | 0.229 | 0.997 |
|  | L | Cognitive disability vs Motor disability | SPMS vs PPMS | -0.109 | 0.23 | 0.997 |
| Pons |  | Cognitive disability vs Motor disability | SPMS vs PPMS | 0.168 | 0.224 | 0.997 |
| Brain Stem |  | Cognitive disability vs Motor disability | SPMS vs PPMS | 0.077 | 0.224 | 0.997 |
| Caudate | R | Cognitive disability vs Motor disability | SPMS vs PPMS | 0.142 | 0.215 | 0.997 |
|  | L | Cognitive disability vs Motor disability | SPMS vs PPMS | 0.005 | 0.224 | 0.997 |
| Cerebellum Exterior | R | Cognitive disability vs Motor disability | SPMS vs PPMS | 0.030 | 0.226 | 0.997 |
|  | L | Cognitive disability vs Motor disability | SPMS vs PPMS | 0.119 | 0.223 | 0.997 |
| Hippocampus | R | Cognitive disability vs Motor disability | SPMS vs PPMS | -0.159 | 0.224 | 0.997 |
|  | L | Cognitive disability vs Motor disability | SPMS vs PPMS | -0.145 | 0.23 | 0.997 |
| Pallidum | R | Cognitive disability vs Motor disability | SPMS vs PPMS | -0.202 | 0.224 | 0.997 |
|  | L | Cognitive disability vs Motor disability | SPMS vs PPMS | -0.106 | 0.211 | 0.997 |
| Putamen | R | Cognitive disability vs Motor disability | SPMS vs PPMS | 0.014 | 0.219 | 0.997 |
|  | L | Cognitive disability vs Motor disability | SPMS vs PPMS | -0.078 | 0.222 | 0.997 |
| Thalamus | R | Cognitive disability vs Motor disability | SPMS vs PPMS | -0.034 | 0.224 | 0.997 |
|  | L | Cognitive disability vs Motor disability | SPMS vs PPMS | -0.036 | 0.224 | 0.997 |
| Ventral diencephalon | R | Cognitive disability vs Motor disability | SPMS vs PPMS | 0.146 | 0.226 | 0.997 |
|  | L | Cognitive disability vs Motor disability | SPMS vs PPMS | 0.185 | 0.227 | 0.997 |
| Cerebellar Vermal Lobules I V |  | Cognitive disability vs Motor disability | SPMS vs PPMS | 0.416 | 0.227 | 0.997 |
| Cerebellar Vermal Lobules VI VII |  | Cognitive disability vs Motor disability | SPMS vs PPMS | 0.131 | 0.233 | 0.997 |
| Cerebellar Vermal Lobules VIII X |  | Cognitive disability vs Motor disability | SPMS vs PPMS | 0.013 | 0.229 | 0.997 |
| Basal Forebrain | L | Cognitive disability vs Motor disability | SPMS vs PPMS | -0.193 | 0.23 | 0.997 |
|  | R | Cognitive disability vs Motor disability | SPMS vs PPMS | -0.054 | 0.228 | 0.997 |
| Anterior cingulate gyrus | R | Cognitive disability vs Motor disability | SPMS vs PPMS | -0.227 | 0.227 | 0.997 |
|  | L | Cognitive disability vs Motor disability | SPMS vs PPMS | -0.608 | 0.226 | 0.997 |
| Anterior insula | R | Cognitive disability vs Motor disability | SPMS vs PPMS | 0.182 | 0.226 | 0.997 |
|  | L | Cognitive disability vs Motor disability | SPMS vs PPMS | 0.367 | 0.222 | 0.997 |
| Anterior orbital gyrus | R | Cognitive disability vs Motor disability | SPMS vs PPMS | 0.132 | 0.218 | 0.997 |
|  | L | Cognitive disability vs Motor disability | SPMS vs PPMS | -0.020 | 0.222 | 0.997 |
| Angular gyrus | R | Cognitive disability vs Motor disability | SPMS vs PPMS | -0.099 | 0.227 | 0.997 |
|  | L | Cognitive disability vs Motor disability | SPMS vs PPMS | 0.235 | 0.229 | 0.997 |
| Calcarine cortex | R | Cognitive disability vs Motor disability | SPMS vs PPMS | 0.054 | 0.227 | 0.997 |
|  | L | Cognitive disability vs Motor disability | SPMS vs PPMS | 0.189 | 0.23 | 0.997 |
| Central operculum | R | Cognitive disability vs Motor disability | SPMS vs PPMS | -0.197 | 0.227 | 0.997 |
|  | L | Cognitive disability vs Motor disability | SPMS vs PPMS | -0.127 | 0.226 | 0.997 |
| Cuneus | R | Cognitive disability vs Motor disability | SPMS vs PPMS | 0.025 | 0.227 | 0.997 |
|  | L | Cognitive disability vs Motor disability | SPMS vs PPMS | 0.008 | 0.227 | 0.997 |
| Entorhinal area | R | Cognitive disability vs Motor disability | SPMS vs PPMS | 0.043 | 0.23 | 0.997 |
|  | L | Cognitive disability vs Motor disability | SPMS vs PPMS | 0.089 | 0.23 | 0.997 |
| Frontal operculum | R | Cognitive disability vs Motor disability | SPMS vs PPMS | 0.097 | 0.226 | 0.997 |
|  | L | Cognitive disability vs Motor disability | SPMS vs PPMS | 0.132 | 0.224 | 0.997 |
| Frontal pole | R | Cognitive disability vs Motor disability | SPMS vs PPMS | -0.200 | 0.213 | 0.997 |
|  | L | Cognitive disability vs Motor disability | SPMS vs PPMS | -0.102 | 0.214 | 0.997 |
| Fusiform gyrus | R | Cognitive disability vs Motor disability | SPMS vs PPMS | -0.094 | 0.222 | 0.997 |
|  | L | Cognitive disability vs Motor disability | SPMS vs PPMS | -0.062 | 0.226 | 0.997 |
| Gyrus rectus | R | Cognitive disability vs Motor disability | SPMS vs PPMS | -0.174 | 0.228 | 0.997 |
|  | L | Cognitive disability vs Motor disability | SPMS vs PPMS | -0.101 | 0.228 | 0.997 |
| Inferior occipital gyrus | R | Cognitive disability vs Motor disability | SPMS vs PPMS | -0.122 | 0.232 | 0.997 |
|  | L | Cognitive disability vs Motor disability | SPMS vs PPMS | 0.073 | 0.231 | 0.997 |
| Inferior temporal gyrus | R | Cognitive disability vs Motor disability | SPMS vs PPMS | -0.255 | 0.226 | 0.997 |
|  | L | Cognitive disability vs Motor disability | SPMS vs PPMS | 0.139 | 0.22 | 0.997 |
| Lingual gyrus | R | Cognitive disability vs Motor disability | SPMS vs PPMS | 0.080 | 0.223 | 0.997 |
|  | L | Cognitive disability vs Motor disability | SPMS vs PPMS | 0.176 | 0.221 | 0.997 |
| Lateral orbital gyrus | R | Cognitive disability vs Motor disability | SPMS vs PPMS | -0.129 | 0.232 | 0.997 |
|  | L | Cognitive disability vs Motor disability | SPMS vs PPMS | -0.416 | 0.231 | 0.997 |
| Middle cingulate gyrus | R | Cognitive disability vs Motor disability | SPMS vs PPMS | -0.238 | 0.229 | 0.997 |
|  | L | Cognitive disability vs Motor disability | SPMS vs PPMS | 0.087 | 0.225 | 0.997 |
| Medial frontal cortex | R | Cognitive disability vs Motor disability | SPMS vs PPMS | 0.218 | 0.222 | 0.997 |
|  | L | Cognitive disability vs Motor disability | SPMS vs PPMS | -0.127 | 0.227 | 0.997 |
| Middle frontal gyrus | R | Cognitive disability vs Motor disability | SPMS vs PPMS | -0.325 | 0.227 | 0.997 |
|  | L | Cognitive disability vs Motor disability | SPMS vs PPMS | 0.051 | 0.227 | 0.997 |
| Middle occipital gyrus | R | Cognitive disability vs Motor disability | SPMS vs PPMS | 0.148 | 0.225 | 0.997 |
|  | L | Cognitive disability vs Motor disability | SPMS vs PPMS | 0.343 | 0.227 | 0.997 |
| Medial orbital gyrus | R | Cognitive disability vs Motor disability | SPMS vs PPMS | -0.132 | 0.228 | 0.997 |
|  | L | Cognitive disability vs Motor disability | SPMS vs PPMS | -0.231 | 0.23 | 0.997 |
| Postcentral gyrus medial segment | R | Cognitive disability vs Motor disability | SPMS vs PPMS | -0.060 | 0.231 | 0.997 |
|  | L | Cognitive disability vs Motor disability | SPMS vs PPMS | -0.342 | 0.229 | 0.997 |
| Precentral gyrus medial segment | R | Cognitive disability vs Motor disability | SPMS vs PPMS | -0.096 | 0.233 | 0.997 |
|  | L | Cognitive disability vs Motor disability | SPMS vs PPMS | 0.045 | 0.229 | 0.997 |
| Superior frontal gyrus medial segment | R | Cognitive disability vs Motor disability | SPMS vs PPMS | 0.042 | 0.221 | 0.997 |
|  | L | Cognitive disability vs Motor disability | SPMS vs PPMS | 0.337 | 0.219 | 0.997 |
| Middle temporal gyrus | R | Cognitive disability vs Motor disability | SPMS vs PPMS | -0.106 | 0.218 | 0.997 |
|  | L | Cognitive disability vs Motor disability | SPMS vs PPMS | 0.322 | 0.224 | 0.997 |
| Occipital pole | R | Cognitive disability vs Motor disability | SPMS vs PPMS | -0.005 | 0.216 | 0.997 |
|  | L | Cognitive disability vs Motor disability | SPMS vs PPMS | 0.020 | 0.22 | 0.997 |
| Occipital fusiform gyrus | R | Cognitive disability vs Motor disability | SPMS vs PPMS | 0.182 | 0.227 | 0.997 |
|  | L | Cognitive disability vs Motor disability | SPMS vs PPMS | 0.414 | 0.225 | 0.997 |
| Opercular part of the inferior frontal gyrus | R | Cognitive disability vs Motor disability | SPMS vs PPMS | 0.065 | 0.232 | 0.997 |
|  | L | Cognitive disability vs Motor disability | SPMS vs PPMS | 0.180 | 0.228 | 0.997 |
| Orbital part of the inferior frontal gyrus | R | Cognitive disability vs Motor disability | SPMS vs PPMS | 0.106 | 0.224 | 0.997 |
|  | L | Cognitive disability vs Motor disability | SPMS vs PPMS | 0.295 | 0.23 | 0.997 |
| Posterior cingulate gyrus | R | Cognitive disability vs Motor disability | SPMS vs PPMS | 0.236 | 0.224 | 0.997 |
|  | L | Cognitive disability vs Motor disability | SPMS vs PPMS | 0.001 | 0.223 | 0.998 |
| Precuneus | R | Cognitive disability vs Motor disability | SPMS vs PPMS | -0.010 | 0.225 | 0.997 |
|  | L | Cognitive disability vs Motor disability | SPMS vs PPMS | 0.070 | 0.227 | 0.997 |
| Parahippocampal gyrus | R | Cognitive disability vs Motor disability | SPMS vs PPMS | 0.555 | 0.229 | 0.997 |
|  | L | Cognitive disability vs Motor disability | SPMS vs PPMS | -0.013 | 0.23 | 0.997 |
| Posterior insula | R | Cognitive disability vs Motor disability | SPMS vs PPMS | -0.234 | 0.226 | 0.997 |
|  | L | Cognitive disability vs Motor disability | SPMS vs PPMS | -0.183 | 0.227 | 0.997 |
| Parietal operculum | R | Cognitive disability vs Motor disability | SPMS vs PPMS | -0.443 | 0.227 | 0.997 |
|  | L | Cognitive disability vs Motor disability | SPMS vs PPMS | -0.308 | 0.229 | 0.997 |
| Postcentral gyrus | R | Cognitive disability vs Motor disability | SPMS vs PPMS | 0.001 | 0.227 | 0.998 |
|  | L | Cognitive disability vs Motor disability | SPMS vs PPMS | -0.400 | 0.23 | 0.997 |
| Posterior orbital gyrus | R | Cognitive disability vs Motor disability | SPMS vs PPMS | -0.070 | 0.222 | 0.997 |
|  | L | Cognitive disability vs Motor disability | SPMS vs PPMS | -0.169 | 0.222 | 0.997 |
| Planum polare | R | Cognitive disability vs Motor disability | SPMS vs PPMS | -0.449 | 0.23 | 0.997 |
|  | L | Cognitive disability vs Motor disability | SPMS vs PPMS | -0.066 | 0.23 | 0.997 |
| Precentral gyrus | R | Cognitive disability vs Motor disability | SPMS vs PPMS | -0.178 | 0.227 | 0.997 |
|  | L | Cognitive disability vs Motor disability | SPMS vs PPMS | -0.339 | 0.228 | 0.997 |
| Planum temporale | R | Cognitive disability vs Motor disability | SPMS vs PPMS | -0.374 | 0.223 | 0.997 |
|  | L | Cognitive disability vs Motor disability | SPMS vs PPMS | 0.134 | 0.227 | 0.997 |
| Subcallosal area | R | Cognitive disability vs Motor disability | SPMS vs PPMS | -0.024 | 0.231 | 0.997 |
|  | L | Cognitive disability vs Motor disability | SPMS vs PPMS | -0.247 | 0.233 | 0.997 |
| Superior frontal gyrus | R | Cognitive disability vs Motor disability | SPMS vs PPMS | 0.088 | 0.214 | 0.997 |
|  | L | Cognitive disability vs Motor disability | SPMS vs PPMS | 0.072 | 0.217 | 0.997 |
| Supplementary motor cortex | R | Cognitive disability vs Motor disability | SPMS vs PPMS | -0.085 | 0.226 | 0.997 |
|  | L | Cognitive disability vs Motor disability | SPMS vs PPMS | -0.038 | 0.225 | 0.997 |
| Supramarginal gyrus | R | Cognitive disability vs Motor disability | SPMS vs PPMS | -0.220 | 0.227 | 0.997 |
|  | L | Cognitive disability vs Motor disability | SPMS vs PPMS | -0.310 | 0.229 | 0.997 |
| Superior occipital gyrus | R | Cognitive disability vs Motor disability | SPMS vs PPMS | -0.015 | 0.229 | 0.997 |
|  | L | Cognitive disability vs Motor disability | SPMS vs PPMS | 0.154 | 0.232 | 0.997 |
| Superior parietal lobule | R | Cognitive disability vs Motor disability | SPMS vs PPMS | 0.021 | 0.225 | 0.997 |
|  | L | Cognitive disability vs Motor disability | SPMS vs PPMS | 0.095 | 0.225 | 0.997 |
| Superior temporal gyrus | R | Cognitive disability vs Motor disability | SPMS vs PPMS | 0.026 | 0.223 | 0.997 |
|  | L | Cognitive disability vs Motor disability | SPMS vs PPMS | 0.144 | 0.222 | 0.997 |
| Temporal pole | R | Cognitive disability vs Motor disability | SPMS vs PPMS | 0.235 | 0.230 | 0.997 |
|  | L | Cognitive disability vs Motor disability | SPMS vs PPMS | 0.476 | 0.229 | 0.997 |
| Triangular part of the inferior frontal gyrus | R | Cognitive disability vs Motor disability | SPMS vs PPMS | -0.304 | 0.227 | 0.997 |
|  | L | Cognitive disability vs Motor disability | SPMS vs PPMS | -0.089 | 0.227 | 0.997 |
| Transverse temporal gyrus | R | Cognitive disability vs Motor disability | SPMS vs PPMS | -0.111 | 0.233 | 0.997 |
|  | L | Cognitive disability vs Motor disability | SPMS vs PPMS | -0.088 | 0.230 | 0.997 |

**Abbreviations**: L= left, R = right, SE = standard error, PPMS = Primary Progressive Multiple Sclerosis, SPMS = Secondary Progressive Multiple Sclerosis; FDR = False Discovery Rate.

**Supplementary Table 8.** Summarizes disability profile × clinical phenotype (SPMS vs PPMS) interaction terms for IC loadings of regional volume patterns.

| **MRI metric** | **Profile** | **Phenotype** | **β coef** | **SE** | **FDR-corrected**  **p-values** |
| --- | --- | --- | --- | --- | --- |
| **IC1** | Global disability vs Motor disability | SPMS vs PPMS | -0.169 | 0.196 | 0.934 |
| **IC2** | Global disability vs Motor disability | SPMS vs PPMS | -0.264 | 0.207 | 0.934 |
| **IC3** | Global disability vs Motor disability | SPMS vs PPMS | -0.276 | 0.218 | 0.934 |
| **IC4** | Global disability vs Motor disability | SPMS vs PPMS | 0.147 | 0.21 | 0.934 |
| **IC5** | Global disability vs Motor disability | SPMS vs PPMS | -0.103 | 0.218 | 0.934 |
| **IC6** | Global disability vs Motor disability | SPMS vs PPMS | 0.428 | 0.214 | 0.919 |
| **IC7** | Global disability vs Motor disability | SPMS vs PPMS | -0.146 | 0.21 | 0.934 |
| **IC8** | Global disability vs Motor disability | SPMS vs PPMS | 0.063 | 0.214 | 0.934 |
| **IC9** | Global disability vs Motor disability | SPMS vs PPMS | 0.008 | 0.214 | 0.994 |
| **IC10** | Global disability vs Motor disability | SPMS vs PPMS | 0.029 | 0.202 | 0.934 |
| **IC11** | Global disability vs Motor disability | SPMS vs PPMS | -0.186 | 0.215 | 0.934 |
| **IC12** | Global disability vs Motor disability | SPMS vs PPMS | -0.206 | 0.21 | 0.934 |
| **IC13** | Global disability vs Motor disability | SPMS vs PPMS | -0.103 | 0.214 | 0.934 |
| **IC14** | Global disability vs Motor disability | SPMS vs PPMS | 0.035 | 0.216 | 0.934 |
| **IC15** | Global disability vs Motor disability | SPMS vs PPMS | 0.053 | 0.203 | 0.934 |
| **IC16** | Global disability vs Motor disability | SPMS vs PPMS | 0.213 | 0.215 | 0.934 |
| **IC17** | Global disability vs Motor disability | SPMS vs PPMS | -0.141 | 0.214 | 0.934 |
| **IC18** | Global disability vs Motor disability | SPMS vs PPMS | 0.059 | 0.216 | 0.934 |
| **IC19** | Global disability vs Motor disability | SPMS vs PPMS | -0.103 | 0.209 | 0.934 |
| **IC20** | Global disability vs Motor disability | SPMS vs PPMS | -0.226 | 0.208 | 0.934 |
| **IC1** | Cognitive disability vs Motor disability | SPMS vs PPMS | 0.036 | 0.208 | 0.934 |
| **IC2** | Cognitive disability vs Motor disability | SPMS vs PPMS | 0.002 | 0.22 | 0.994 |
| **IC3** | Cognitive disability vs Motor disability | SPMS vs PPMS | -0.067 | 0.23 | 0.934 |
| **IC4** | Cognitive disability vs Motor disability | SPMS vs PPMS | 0.265 | 0.223 | 0.934 |
| **IC5** | Cognitive disability vs Motor disability | SPMS vs PPMS | -0.199 | 0.231 | 0.934 |
| **IC6** | Cognitive disability vs Motor disability | SPMS vs PPMS | 0.217 | 0.227 | 0.934 |
| **IC7** | Cognitive disability vs Motor disability | SPMS vs PPMS | -0.093 | 0.222 | 0.934 |
| **IC8** | Cognitive disability vs Motor disability | SPMS vs PPMS | 0.231 | 0.226 | 0.934 |
| **IC9** | Cognitive disability vs Motor disability | SPMS vs PPMS | -0.132 | 0.226 | 0.934 |
| **IC10** | Cognitive disability vs Motor disability | SPMS vs PPMS | 0.225 | 0.214 | 0.934 |
| **IC11** | Cognitive disability vs Motor disability | SPMS vs PPMS | -0.033 | 0.227 | 0.934 |
| **IC12** | Cognitive disability vs Motor disability | SPMS vs PPMS | 0.039 | 0.223 | 0.934 |
| **IC13** | Cognitive disability vs Motor disability | SPMS vs PPMS | -0.041 | 0.227 | 0.934 |
| **IC14** | Cognitive disability vs Motor disability | SPMS vs PPMS | 0.265 | 0.229 | 0.934 |
| **IC15** | Cognitive disability vs Motor disability | SPMS vs PPMS | 0.154 | 0.215 | 0.934 |
| **IC16** | Cognitive disability vs Motor disability | SPMS vs PPMS | 0.15 | 0.228 | 0.934 |
| **IC17** | Cognitive disability vs Motor disability | SPMS vs PPMS | -0.463 | 0.227 | 0.919 |
| **IC18** | Cognitive disability vs Motor disability | SPMS vs PPMS | 0.192 | 0.229 | 0.934 |
| **IC19** | Cognitive disability vs Motor disability | SPMS vs PPMS | -0.08 | 0.222 | 0.934 |
| **IC20** | Cognitive disability vs Motor disability | SPMS vs PPMS | 0.055 | 0.221 | 0.934 |

**Abbreviations**: IC = independent component, SE = standard error, PPMS = Primary Progressive Multiple Sclerosis; SPMS = Secondary Progressive Multiple Sclerosis, FDR=False Discovery Rate.

**Supplementary Table 9.** Summarizes disability profile × clinical phenotype (SPMS vs PPMS) interaction terms for regional disconnection.

| **MRI metric** | **Side** | **Profile** | **Phenotype** | **β coef** | **SE** | **FDR-corrected p- values** |
| --- | --- | --- | --- | --- | --- | --- |
| Accumbens | R | Global disability vs Motor disability | SPMS vs PPMS | 0.464 | 0.208 | 0.357 |
|  | L | Global disability vs Motor disability | SPMS vs PPMS | 0.420 | 0.209 | 0.357 |
| Amygdala | R | Global disability vs Motor disability | SPMS vs PPMS | 0.293 | 0.208 | 0.469 |
|  | L | Global disability vs Motor disability | SPMS vs PPMS | 0.312 | 0.206 | 0.469 |
| Pons |  | Global disability vs Motor disability | SPMS vs PPMS | 0.287 | 0.203 | 0.469 |
| Brain Stem |  | Global disability vs Motor disability | SPMS vs PPMS | 0.333 | 0.202 | 0.427 |
| Caudate Nucleus | R | Global disability vs Motor disability | SPMS vs PPMS | 0.332 | 0.209 | 0.445 |
|  | L | Global disability vs Motor disability | SPMS vs PPMS | 0.398 | 0.207 | 0.357 |
| Cerebellum Exterior | R | Global disability vs Motor disability | SPMS vs PPMS | 0.433 | 0.203 | 0.357 |
|  | L | Global disability vs Motor disability | SPMS vs PPMS | 0.251 | 0.203 | 0.533 |
| Hippocampus | R | Global disability vs Motor disability | SPMS vs PPMS | 0.276 | 0.209 | 0.506 |
|  | L | Global disability vs Motor disability | SPMS vs PPMS | 0.214 | 0.205 | 0.572 |
| Pallidum | R | Global disability vs Motor disability | SPMS vs PPMS | 0.437 | 0.206 | 0.357 |
|  | L | Global disability vs Motor disability | SPMS vs PPMS | 0.454 | 0.207 | 0.357 |
| Putamen | R | Global disability vs Motor disability | SPMS vs PPMS | 0.419 | 0.206 | 0.357 |
|  | L | Global disability vs Motor disability | SPMS vs PPMS | 0.442 | 0.203 | 0.357 |
| Thalamus Proper | R | Global disability vs Motor disability | SPMS vs PPMS | 0.375 | 0.204 | 0.357 |
|  | L | Global disability vs Motor disability | SPMS vs PPMS | 0.469 | 0.201 | 0.357 |
| Ventral DC | R | Global disability vs Motor disability | SPMS vs PPMS | 0.305 | 0.204 | 0.469 |
|  | L | Global disability vs Motor disability | SPMS vs PPMS | 0.338 | 0.202 | 0.423 |
| Cerebellar Vermal Lobules I V |  | Global disability vs Motor disability | SPMS vs PPMS | 0.487 | 0.202 | 0.357 |
| Cerebellar Vermal Lobules VI VII |  | Global disability vs Motor disability | SPMS vs PPMS | -0.059 | 0.216 | 0.878 |
| Cerebellar Vermal Lobules VIII X |  | Global disability vs Motor disability | SPMS vs PPMS | 0.501 | 0.204 | 0.357 |
| Basal Forebrain | L | Global disability vs Motor disability | SPMS vs PPMS | 0.431 | 0.207 | 0.357 |
|  | R | Global disability vs Motor disability | SPMS vs PPMS | 0.560 | 0.207 | 0.357 |
| Anterior cingulate gyrus | R | Global disability vs Motor disability | SPMS vs PPMS | 0.336 | 0.207 | 0.436 |
|  | L | Global disability vs Motor disability | SPMS vs PPMS | 0.247 | 0.208 | 0.546 |
| Anterior insula | R | Global disability vs Motor disability | SPMS vs PPMS | 0.274 | 0.207 | 0.506 |
|  | L | Global disability vs Motor disability | SPMS vs PPMS | 0.420 | 0.203 | 0.357 |
| Anterior orbital gyrus | R | Global disability vs Motor disability | SPMS vs PPMS | 0.332 | 0.210 | 0.45 |
|  | L | Global disability vs Motor disability | SPMS vs PPMS | 0.418 | 0.205 | 0.357 |
| Angular gyrus | R | Global disability vs Motor disability | SPMS vs PPMS | 0.211 | 0.207 | 0.576 |
|  | L | Global disability vs Motor disability | SPMS vs PPMS | 0.214 | 0.208 | 0.572 |
| Calcarine cortex | R | Global disability vs Motor disability | SPMS vs PPMS | 0.278 | 0.213 | 0.514 |
|  | L | Global disability vs Motor disability | SPMS vs PPMS | 0.326 | 0.209 | 0.459 |
| Central operculum | R | Global disability vs Motor disability | SPMS vs PPMS | 0.416 | 0.210 | 0.357 |
|  | L | Global disability vs Motor disability | SPMS vs PPMS | 0.181 | 0.209 | 0.634 |
| Cuneus | R | Global disability vs Motor disability | SPMS vs PPMS | 0.228 | 0.208 | 0.559 |
|  | L | Global disability vs Motor disability | SPMS vs PPMS | 0.364 | 0.209 | 0.386 |
| Entorhinal area | R | Global disability vs Motor disability | SPMS vs PPMS | 0.244 | 0.210 | 0.546 |
|  | L | Global disability vs Motor disability | SPMS vs PPMS | 0.011 | 0.211 | 0.972 |
| Frontal operculum | R | Global disability vs Motor disability | SPMS vs PPMS | 0.138 | 0.212 | 0.715 |
|  | L | Global disability vs Motor disability | SPMS vs PPMS | 0.355 | 0.207 | 0.406 |
| Frontal pole | R | Global disability vs Motor disability | SPMS vs PPMS | 0.262 | 0.208 | 0.524 |
|  | L | Global disability vs Motor disability | SPMS vs PPMS | 0.377 | 0.204 | 0.357 |
| Fusiform gyrus | R | Global disability vs Motor disability | SPMS vs PPMS | 0.195 | 0.210 | 0.607 |
|  | L | Global disability vs Motor disability | SPMS vs PPMS | 0.043 | 0.211 | 0.909 |
| Gyrus rectus | R | Global disability vs Motor disability | SPMS vs PPMS | 0.246 | 0.213 | 0.546 |
|  | L | Global disability vs Motor disability | SPMS vs PPMS | -0.144 | 0.215 | 0.704 |
| Inferior occipital gyrus | R | Global disability vs Motor disability | SPMS vs PPMS | 0.150 | 0.214 | 0.693 |
|  | L | Global disability vs Motor disability | SPMS vs PPMS | 0.263 | 0.207 | 0.521 |
| Inferior temporal gyrus | R | Global disability vs Motor disability | SPMS vs PPMS | 0.377 | 0.206 | 0.357 |
|  | L | Global disability vs Motor disability | SPMS vs PPMS | 0.362 | 0.205 | 0.38 |
| Lingual gyrus | R | Global disability vs Motor disability | SPMS vs PPMS | 0.213 | 0.211 | 0.579 |
|  | L | Global disability vs Motor disability | SPMS vs PPMS | 0.442 | 0.211 | 0.357 |
| Lateral orbital gyrus | R | Global disability vs Motor disability | SPMS vs PPMS | 0.204 | 0.210 | 0.594 |
|  | L | Global disability vs Motor disability | SPMS vs PPMS | 0.339 | 0.206 | 0.427 |
| Middle cingulate gyrus | R | Global disability vs Motor disability | SPMS vs PPMS | 0.303 | 0.206 | 0.469 |
|  | L | Global disability vs Motor disability | SPMS vs PPMS | 0.226 | 0.203 | 0.556 |
| Medial frontal cortex | R | Global disability vs Motor disability | SPMS vs PPMS | -0.159 | 0.213 | 0.666 |
|  | L | Global disability vs Motor disability | SPMS vs PPMS | -0.113 | 0.221 | 0.784 |
| Middle frontal gyrus | R | Global disability vs Motor disability | SPMS vs PPMS | 0.260 | 0.205 | 0.521 |
|  | L | Global disability vs Motor disability | SPMS vs PPMS | 0.434 | 0.203 | 0.357 |
| Middle occipital gyrus | R | Global disability vs Motor disability | SPMS vs PPMS | 0.100 | 0.209 | 0.803 |
|  | L | Global disability vs Motor disability | SPMS vs PPMS | 0.295 | 0.206 | 0.469 |
| Medial orbital gyrus | R | Global disability vs Motor disability | SPMS vs PPMS | 0.582 | 0.212 | 0.357 |
|  | L | Global disability vs Motor disability | SPMS vs PPMS | 0.368 | 0.204 | 0.36 |
| Postcentral gyrus medial segment | R | Global disability vs Motor disability | SPMS vs PPMS | 0.379 | 0.208 | 0.357 |
|  | L | Global disability vs Motor disability | SPMS vs PPMS | 0.198 | 0.214 | 0.607 |
| Precentral gyrus medial segment | R | Global disability vs Motor disability | SPMS vs PPMS | 0.340 | 0.210 | 0.436 |
|  | L | Global disability vs Motor disability | SPMS vs PPMS | 0.423 | 0.209 | 0.357 |
| Superior frontal gyrus medial segment | R | Global disability vs Motor disability | SPMS vs PPMS | 0.481 | 0.205 | 0.357 |
|  | L | Global disability vs Motor disability | SPMS vs PPMS | 0.415 | 0.205 | 0.357 |
| Middle temporal gyrus | R | Global disability vs Motor disability | SPMS vs PPMS | 0.264 | 0.208 | 0.521 |
|  | L | Global disability vs Motor disability | SPMS vs PPMS | 0.402 | 0.205 | 0.357 |
| Occipital pole | R | Global disability vs Motor disability | SPMS vs PPMS | 0.151 | 0.213 | 0.687 |
|  | L | Global disability vs Motor disability | SPMS vs PPMS | 0.380 | 0.206 | 0.357 |
| Occipital fusiform gyrus | R | Global disability vs Motor disability | SPMS vs PPMS | 0.306 | 0.217 | 0.469 |
|  | L | Global disability vs Motor disability | SPMS vs PPMS | 0.402 | 0.207 | 0.357 |
| Opercular part of the inferior frontal gyrus | R | Global disability vs Motor disability | SPMS vs PPMS | 0.230 | 0.206 | 0.556 |
|  | L | Global disability vs Motor disability | SPMS vs PPMS | 0.458 | 0.207 | 0.357 |
| Orbital part of the inferior frontal gyrus | R | Global disability vs Motor disability | SPMS vs PPMS | 0.176 | 0.213 | 0.637 |
|  | L | Global disability vs Motor disability | SPMS vs PPMS | 0.338 | 0.205 | 0.427 |
| Posterior cingulate gyrus | R | Global disability vs Motor disability | SPMS vs PPMS | 0.278 | 0.208 | 0.506 |
|  | L | Global disability vs Motor disability | SPMS vs PPMS | 0.192 | 0.208 | 0.607 |
| Precuneus | R | Global disability vs Motor disability | SPMS vs PPMS | 0.376 | 0.206 | 0.357 |
|  | L | Global disability vs Motor disability | SPMS vs PPMS | 0.278 | 0.206 | 0.501 |
| Parahippocampal gyrus | R | Global disability vs Motor disability | SPMS vs PPMS | 0.230 | 0.208 | 0.556 |
|  | L | Global disability vs Motor disability | SPMS vs PPMS | 0.193 | 0.201 | 0.602 |
| Posterior insula | R | Global disability vs Motor disability | SPMS vs PPMS | 0.299 | 0.205 | 0.469 |
|  | L | Global disability vs Motor disability | SPMS vs PPMS | 0.412 | 0.203 | 0.357 |
| Parietal operculum | R | Global disability vs Motor disability | SPMS vs PPMS | 0.178 | 0.210 | 0.635 |
|  | L | Global disability vs Motor disability | SPMS vs PPMS | 0.381 | 0.207 | 0.357 |
| Postcentral gyrus | R | Global disability vs Motor disability | SPMS vs PPMS | 0.314 | 0.206 | 0.469 |
|  | L | Global disability vs Motor disability | SPMS vs PPMS | 0.286 | 0.202 | 0.469 |
| Posterior orbital gyrus | R | Global disability vs Motor disability | SPMS vs PPMS | 0.229 | 0.215 | 0.57 |
|  | L | Global disability vs Motor disability | SPMS vs PPMS | 0.292 | 0.206 | 0.469 |
| Planum polare | R | Global disability vs Motor disability | SPMS vs PPMS | 0.261 | 0.206 | 0.521 |
|  | L | Global disability vs Motor disability | SPMS vs PPMS | 0.392 | 0.205 | 0.357 |
| Precentral gyrus | R | Global disability vs Motor disability | SPMS vs PPMS | 0.398 | 0.204 | 0.357 |
|  | L | Global disability vs Motor disability | SPMS vs PPMS | 0.253 | 0.204 | 0.533 |
| Planum temporale | R | Global disability vs Motor disability | SPMS vs PPMS | -0.070 | 0.210 | 0.858 |
|  | L | Global disability vs Motor disability | SPMS vs PPMS | 0.281 | 0.208 | 0.501 |
| Subcallosal area | R | Global disability vs Motor disability | SPMS vs PPMS | 0.210 | 0.214 | 0.593 |
|  | L | Global disability vs Motor disability | SPMS vs PPMS | 0.056 | 0.216 | 0.882 |
| Superior frontal gyrus | R | Global disability vs Motor disability | SPMS vs PPMS | 0.399 | 0.204 | 0.357 |
|  | L | Global disability vs Motor disability | SPMS vs PPMS | 0.425 | 0.202 | 0.357 |
| Supplementary motor cortex | R | Global disability vs Motor disability | SPMS vs PPMS | 0.265 | 0.207 | 0.521 |
|  | L | Global disability vs Motor disability | SPMS vs PPMS | 0.388 | 0.205 | 0.357 |
| Supramarginal gyrus | R | Global disability vs Motor disability | SPMS vs PPMS | 0.345 | 0.205 | 0.422 |
|  | L | Global disability vs Motor disability | SPMS vs PPMS | 0.320 | 0.208 | 0.469 |
| Superior occipital gyrus | R | Global disability vs Motor disability | SPMS vs PPMS | 0.228 | 0.209 | 0.559 |
|  | L | Global disability vs Motor disability | SPMS vs PPMS | 0.368 | 0.205 | 0.36 |
| Superior parietal lobule | R | Global disability vs Motor disability | SPMS vs PPMS | 0.307 | 0.203 | 0.469 |
|  | L | Global disability vs Motor disability | SPMS vs PPMS | 0.378 | 0.202 | 0.357 |
| Superior temporal gyrus | R | Global disability vs Motor disability | SPMS vs PPMS | 0.071 | 0.211 | 0.858 |
|  | L | Global disability vs Motor disability | SPMS vs PPMS | 0.332 | 0.206 | 0.437 |
| Temporal pole | R | Global disability vs Motor disability | SPMS vs PPMS | 0.549 | 0.206 | 0.357 |
|  | L | Global disability vs Motor disability | SPMS vs PPMS | 0.405 | 0.205 | 0.357 |
| Triangular part of the inferior frontal gyrus | R | Global disability vs Motor disability | SPMS vs PPMS | 0.181 | 0.211 | 0.635 |
|  | L | Global disability vs Motor disability | SPMS vs PPMS | 0.392 | 0.206 | 0.357 |
| Transverse temporal gyrus | R | Global disability vs Motor disability | SPMS vs PPMS | 0.135 | 0.208 | 0.715 |
|  | L | Global disability vs Motor disability | SPMS vs PPMS | 0.567 | 0.208 | 0.357 |
| Accumbens Area | R | Cognitive disability vs Motor disability | SPMS vs PPMS | 0.075 | 0.220 | 0.858 |
|  | L | Cognitive disability vs Motor disability | SPMS vs PPMS | 0.039 | 0.222 | 0.909 |
| Amygdala | R | Cognitive disability vs Motor disability | SPMS vs PPMS | 0.070 | 0.221 | 0.861 |
|  | L | Cognitive disability vs Motor disability | SPMS vs PPMS | 0.254 | 0.218 | 0.546 |
| Pons |  | Cognitive disability vs Motor disability | SPMS vs PPMS | 0.099 | 0.215 | 0.806 |
| Brain Stem |  | Cognitive disability vs Motor disability | SPMS vs PPMS | 0.111 | 0.214 | 0.784 |
| Caudate | R | Cognitive disability vs Motor disability | SPMS vs PPMS | -0.046 | 0.221 | 0.909 |
|  | L | Cognitive disability vs Motor disability | SPMS vs PPMS | -0.018 | 0.219 | 0.953 |
| Cerebellum Exterior | R | Cognitive disability vs Motor disability | SPMS vs PPMS | 0.169 | 0.215 | 0.661 |
|  | L | Cognitive disability vs Motor disability | SPMS vs PPMS | 0.096 | 0.215 | 0.808 |
| Hippocampus | R | Cognitive disability vs Motor disability | SPMS vs PPMS | 0.228 | 0.222 | 0.572 |
|  | L | Cognitive disability vs Motor disability | SPMS vs PPMS | 0.084 | 0.217 | 0.843 |
| Pallidum | R | Cognitive disability vs Motor disability | SPMS vs PPMS | 0.114 | 0.218 | 0.784 |
|  | L | Cognitive disability vs Motor disability | SPMS vs PPMS | 0.166 | 0.220 | 0.666 |
| Putamen | R | Cognitive disability vs Motor disability | SPMS vs PPMS | 0.060 | 0.218 | 0.878 |
|  | L | Cognitive disability vs Motor disability | SPMS vs PPMS | 0.223 | 0.216 | 0.572 |
| Thalamus | R | Cognitive disability vs Motor disability | SPMS vs PPMS | 0.083 | 0.216 | 0.843 |
|  | L | Cognitive disability vs Motor disability | SPMS vs PPMS | 0.188 | 0.213 | 0.624 |
| Ventral diencephalon | R | Cognitive disability vs Motor disability | SPMS vs PPMS | 0.040 | 0.217 | 0.909 |
|  | L | Cognitive disability vs Motor disability | SPMS vs PPMS | 0.137 | 0.214 | 0.719 |
| Cerebellar Vermal Lobules I V |  | Cognitive disability vs Motor disability | SPMS vs PPMS | 0.215 | 0.214 | 0.579 |
| Cerebellar Vermal Lobules VI VII |  | Cognitive disability vs Motor disability | SPMS vs PPMS | 0.041 | 0.229 | 0.909 |
| Cerebellar Vermal Lobules VIII X |  | Cognitive disability vs Motor disability | SPMS vs PPMS | 0.249 | 0.216 | 0.546 |
| Basal Forebrain | L | Cognitive disability vs Motor disability | SPMS vs PPMS | 0.101 | 0.219 | 0.806 |
|  | R | Cognitive disability vs Motor disability | SPMS vs PPMS | 0.120 | 0.219 | 0.776 |
| Anterior cingulate gyrus | R | Cognitive disability vs Motor disability | SPMS vs PPMS | -0.009 | 0.220 | 0.972 |
|  | L | Cognitive disability vs Motor disability | SPMS vs PPMS | 0.032 | 0.220 | 0.917 |
| Anterior insula | R | Cognitive disability vs Motor disability | SPMS vs PPMS | 0.041 | 0.219 | 0.909 |
|  | L | Cognitive disability vs Motor disability | SPMS vs PPMS | 0.316 | 0.215 | 0.469 |
| Anterior orbital gyrus | R | Cognitive disability vs Motor disability | SPMS vs PPMS | -0.009 | 0.223 | 0.972 |
|  | L | Cognitive disability vs Motor disability | SPMS vs PPMS | 0.207 | 0.218 | 0.607 |
| Angular gyrus | R | Cognitive disability vs Motor disability | SPMS vs PPMS | 0.163 | 0.219 | 0.666 |
|  | L | Cognitive disability vs Motor disability | SPMS vs PPMS | 0.204 | 0.220 | 0.607 |
| Calcarine cortex | R | Cognitive disability vs Motor disability | SPMS vs PPMS | 0.016 | 0.226 | 0.958 |
|  | L | Cognitive disability vs Motor disability | SPMS vs PPMS | 0.183 | 0.221 | 0.637 |
| Central operculum | R | Cognitive disability vs Motor disability | SPMS vs PPMS | 0.427 | 0.223 | 0.357 |
|  | L | Cognitive disability vs Motor disability | SPMS vs PPMS | 0.219 | 0.221 | 0.59 |
| Cuneus | R | Cognitive disability vs Motor disability | SPMS vs PPMS | 0.050 | 0.220 | 0.906 |
|  | L | Cognitive disability vs Motor disability | SPMS vs PPMS | 0.171 | 0.221 | 0.663 |
| Entorhinal area | R | Cognitive disability vs Motor disability | SPMS vs PPMS | -0.235 | 0.223 | 0.572 |
|  | L | Cognitive disability vs Motor disability | SPMS vs PPMS | -0.115 | 0.224 | 0.784 |
| Frontal operculum | R | Cognitive disability vs Motor disability | SPMS vs PPMS | -0.042 | 0.225 | 0.909 |
|  | L | Cognitive disability vs Motor disability | SPMS vs PPMS | 0.135 | 0.220 | 0.732 |
| Frontal pole | R | Cognitive disability vs Motor disability | SPMS vs PPMS | -0.129 | 0.221 | 0.746 |
|  | L | Cognitive disability vs Motor disability | SPMS vs PPMS | 0.169 | 0.216 | 0.661 |
| Fusiform gyrus | R | Cognitive disability vs Motor disability | SPMS vs PPMS | 0.037 | 0.222 | 0.913 |
|  | L | Cognitive disability vs Motor disability | SPMS vs PPMS | 0.084 | 0.223 | 0.843 |
| Gyrus rectus | R | Cognitive disability vs Motor disability | SPMS vs PPMS | -0.200 | 0.225 | 0.624 |
|  | L | Cognitive disability vs Motor disability | SPMS vs PPMS | -0.074 | 0.228 | 0.858 |
| Inferior occipital gyrus | R | Cognitive disability vs Motor disability | SPMS vs PPMS | 0.074 | 0.227 | 0.858 |
|  | L | Cognitive disability vs Motor disability | SPMS vs PPMS | 0.267 | 0.220 | 0.543 |
| Inferior temporal gyrus | R | Cognitive disability vs Motor disability | SPMS vs PPMS | 0.181 | 0.218 | 0.637 |
|  | L | Cognitive disability vs Motor disability | SPMS vs PPMS | 0.070 | 0.218 | 0.858 |
| Lingual gyrus | R | Cognitive disability vs Motor disability | SPMS vs PPMS | 0.033 | 0.224 | 0.917 |
|  | L | Cognitive disability vs Motor disability | SPMS vs PPMS | 0.192 | 0.224 | 0.635 |
| Lateral orbital gyrus | R | Cognitive disability vs Motor disability | SPMS vs PPMS | -0.115 | 0.223 | 0.784 |
|  | L | Cognitive disability vs Motor disability | SPMS vs PPMS | 0.249 | 0.218 | 0.546 |
| Middle cingulate gyrus | R | Cognitive disability vs Motor disability | SPMS vs PPMS | 0.254 | 0.218 | 0.546 |
|  | L | Cognitive disability vs Motor disability | SPMS vs PPMS | 0.150 | 0.215 | 0.693 |
| Medial frontal cortex | R | Cognitive disability vs Motor disability | SPMS vs PPMS | -0.109 | 0.226 | 0.803 |
|  | L | Cognitive disability vs Motor disability | SPMS vs PPMS | -0.179 | 0.234 | 0.665 |
| Middle frontal gyrus | R | Cognitive disability vs Motor disability | SPMS vs PPMS | -0.026 | 0.217 | 0.931 |
|  | L | Cognitive disability vs Motor disability | SPMS vs PPMS | 0.306 | 0.215 | 0.469 |
| Middle occipital gyrus | R | Cognitive disability vs Motor disability | SPMS vs PPMS | 0.076 | 0.221 | 0.858 |
|  | L | Cognitive disability vs Motor disability | SPMS vs PPMS | 0.148 | 0.218 | 0.698 |
| Medial orbital gyrus | R | Cognitive disability vs Motor disability | SPMS vs PPMS | 0.060 | 0.224 | 0.879 |
|  | L | Cognitive disability vs Motor disability | SPMS vs PPMS | 0.064 | 0.216 | 0.865 |
| Postcentral gyrus medial segment | R | Cognitive disability vs Motor disability | SPMS vs PPMS | 0.445 | 0.221 | 0.357 |
|  | L | Cognitive disability vs Motor disability | SPMS vs PPMS | 0.192 | 0.227 | 0.635 |
| Precentral gyrus medial segment | R | Cognitive disability vs Motor disability | SPMS vs PPMS | 0.539 | 0.223 | 0.357 |
|  | L | Cognitive disability vs Motor disability | SPMS vs PPMS | 0.443 | 0.222 | 0.357 |
| Superior frontal gyrus medial segment | R | Cognitive disability vs Motor disability | SPMS vs PPMS | 0.100 | 0.218 | 0.806 |
|  | L | Cognitive disability vs Motor disability | SPMS vs PPMS | 0.250 | 0.218 | 0.546 |
| Middle temporal gyrus | R | Cognitive disability vs Motor disability | SPMS vs PPMS | 0.086 | 0.220 | 0.843 |
|  | L | Cognitive disability vs Motor disability | SPMS vs PPMS | 0.198 | 0.217 | 0.615 |
| Occipital pole | R | Cognitive disability vs Motor disability | SPMS vs PPMS | -0.032 | 0.226 | 0.917 |
|  | L | Cognitive disability vs Motor disability | SPMS vs PPMS | 0.257 | 0.219 | 0.546 |
| Occipital fusiform gyrus | R | Cognitive disability vs Motor disability | SPMS vs PPMS | 0.032 | 0.230 | 0.917 |
|  | L | Cognitive disability vs Motor disability | SPMS vs PPMS | 0.319 | 0.219 | 0.469 |
| Opercular part of the inferior frontal gyrus | R | Cognitive disability vs Motor disability | SPMS vs PPMS | 0.084 | 0.218 | 0.843 |
|  | L | Cognitive disability vs Motor disability | SPMS vs PPMS | 0.333 | 0.219 | 0.469 |
| Orbital part of the inferior frontal gyrus | R | Cognitive disability vs Motor disability | SPMS vs PPMS | 0.003 | 0.226 | 0.989 |
|  | L | Cognitive disability vs Motor disability | SPMS vs PPMS | 0.195 | 0.218 | 0.622 |
| Posterior cingulate gyrus | R | Cognitive disability vs Motor disability | SPMS vs PPMS | 0.240 | 0.221 | 0.561 |
|  | L | Cognitive disability vs Motor disability | SPMS vs PPMS | 0.163 | 0.220 | 0.666 |
| Precuneus | R | Cognitive disability vs Motor disability | SPMS vs PPMS | 0.184 | 0.219 | 0.636 |
|  | L | Cognitive disability vs Motor disability | SPMS vs PPMS | 0.247 | 0.218 | 0.547 |
| Parahippocampal gyrus | R | Cognitive disability vs Motor disability | SPMS vs PPMS | 0.100 | 0.220 | 0.807 |
|  | L | Cognitive disability vs Motor disability | SPMS vs PPMS | 0.103 | 0.213 | 0.803 |
| Posterior insula | R | Cognitive disability vs Motor disability | SPMS vs PPMS | -0.068 | 0.218 | 0.861 |
|  | L | Cognitive disability vs Motor disability | SPMS vs PPMS | 0.290 | 0.215 | 0.501 |
| Parietal operculum | R | Cognitive disability vs Motor disability | SPMS vs PPMS | 0.170 | 0.223 | 0.665 |
|  | L | Cognitive disability vs Motor disability | SPMS vs PPMS | 0.311 | 0.220 | 0.469 |
| Postcentral gyrus | R | Cognitive disability vs Motor disability | SPMS vs PPMS | 0.248 | 0.218 | 0.546 |
|  | L | Cognitive disability vs Motor disability | SPMS vs PPMS | 0.264 | 0.214 | 0.534 |
| Posterior orbital gyrus | R | Cognitive disability vs Motor disability | SPMS vs PPMS | 0.040 | 0.227 | 0.909 |
|  | L | Cognitive disability vs Motor disability | SPMS vs PPMS | 0.196 | 0.218 | 0.622 |
| Planum polare | R | Cognitive disability vs Motor disability | SPMS vs PPMS | -0.082 | 0.218 | 0.843 |
|  | L | Cognitive disability vs Motor disability | SPMS vs PPMS | 0.204 | 0.217 | 0.607 |
| Precentral gyrus | R | Cognitive disability vs Motor disability | SPMS vs PPMS | 0.232 | 0.217 | 0.57 |
|  | L | Cognitive disability vs Motor disability | SPMS vs PPMS | 0.093 | 0.217 | 0.82 |
| Planum temporale | R | Cognitive disability vs Motor disability | SPMS vs PPMS | -0.167 | 0.223 | 0.666 |
|  | L | Cognitive disability vs Motor disability | SPMS vs PPMS | 0.132 | 0.221 | 0.739 |
| Subcallosal area | R | Cognitive disability vs Motor disability | SPMS vs PPMS | -0.082 | 0.227 | 0.853 |
|  | L | Cognitive disability vs Motor disability | SPMS vs PPMS | -0.118 | 0.229 | 0.784 |
| Superior frontal gyrus | R | Cognitive disability vs Motor disability | SPMS vs PPMS | 0.147 | 0.216 | 0.698 |
|  | L | Cognitive disability vs Motor disability | SPMS vs PPMS | 0.247 | 0.214 | 0.546 |
| Supplementary motor cortex | R | Cognitive disability vs Motor disability | SPMS vs PPMS | 0.320 | 0.219 | 0.469 |
|  | L | Cognitive disability vs Motor disability | SPMS vs PPMS | 0.313 | 0.217 | 0.469 |
| Supramarginal gyrus | R | Cognitive disability vs Motor disability | SPMS vs PPMS | 0.226 | 0.217 | 0.572 |
|  | L | Cognitive disability vs Motor disability | SPMS vs PPMS | 0.303 | 0.220 | 0.49 |
| Superior occipital gyrus | R | Cognitive disability vs Motor disability | SPMS vs PPMS | 0.047 | 0.221 | 0.909 |
|  | L | Cognitive disability vs Motor disability | SPMS vs PPMS | 0.253 | 0.217 | 0.546 |
| Superior parietal lobule | R | Cognitive disability vs Motor disability | SPMS vs PPMS | 0.173 | 0.215 | 0.653 |
|  | L | Cognitive disability vs Motor disability | SPMS vs PPMS | 0.249 | 0.214 | 0.546 |
| Superior temporal gyrus | R | Cognitive disability vs Motor disability | SPMS vs PPMS | -0.025 | 0.223 | 0.933 |
|  | L | Cognitive disability vs Motor disability | SPMS vs PPMS | 0.225 | 0.219 | 0.572 |
| Temporal pole | R | Cognitive disability vs Motor disability | SPMS vs PPMS | 0.137 | 0.219 | 0.724 |
|  | L | Cognitive disability vs Motor disability | SPMS vs PPMS | 0.170 | 0.217 | 0.661 |
| Triangular part of the inferior frontal gyrus | R | Cognitive disability vs Motor disability | SPMS vs PPMS | -0.042 | 0.224 | 0.909 |
|  | L | Cognitive disability vs Motor disability | SPMS vs PPMS | 0.133 | 0.218 | 0.732 |
| Transverse temporal gyrus | R | Cognitive disability vs Motor disability | SPMS vs PPMS | -0.067 | 0.220 | 0.864 |
|  | L | Cognitive disability vs Motor disability | SPMS vs PPMS | 0.415 | 0.220 | 0.357 |

**Abbreviations**: L = left; R = right, SE = standard error, PPMS = Primary Progressive Multiple Sclerosis; SPMS = Secondary Progressive Multiple Sclerosis, FDR = False Discovery Rate.

**Supplementary Table 10.** Summarizes disability profile × clinical phenotype (SPMS vs PPMS) interaction terms for IC loadings of regional disconnection patterns.

| **MRI metric** | **Profile** | **Phenotype** | **β coef** | **SE** | **FDR-corrected**  **p-values** |
| --- | --- | --- | --- | --- | --- |
| **IC1** | Global disability vs Motor disability | SPMS vs PPMS | -0.292 | 0.214 | 0.768 |
| **IC2** | Global disability vs Motor disability | SPMS vs PPMS | 0.057 | 0.220 | 0.931 |
| **IC3** | Global disability vs Motor disability | SPMS vs PPMS | 0.114 | 0.223 | 0.931 |
| **IC4** | Global disability vs Motor disability | SPMS vs PPMS | -0.115 | 0.219 | 0.931 |
| **IC5** | Global disability vs Motor disability | SPMS vs PPMS | 0.053 | 0.212 | 0.931 |
| **IC6** | Global disability vs Motor disability | SPMS vs PPMS | 0.088 | 0.217 | 0.931 |
| **IC7** | Global disability vs Motor disability | SPMS vs PPMS | -0.221 | 0.224 | 0.931 |
| **IC8** | Global disability vs Motor disability | SPMS vs PPMS | 0.044 | 0.220 | 0.931 |
| **IC9** | Global disability vs Motor disability | SPMS vs PPMS | 0.198 | 0.212 | 0.931 |
| **IC10** | Global disability vs Motor disability | SPMS vs PPMS | -0.042 | 0.218 | 0.931 |
| **IC11** | Global disability vs Motor disability | SPMS vs PPMS | 0.119 | 0.215 | 0.931 |
| **IC12** | Global disability vs Motor disability | SPMS vs PPMS | 0.252 | 0.218 | 0.898 |
| **IC13** | Global disability vs Motor disability | SPMS vs PPMS | 0.315 | 0.208 | 0.768 |
| **IC14** | Global disability vs Motor disability | SPMS vs PPMS | 0.182 | 0.224 | 0.931 |
| **IC15** | Global disability vs Motor disability | SPMS vs PPMS | 0.333 | 0.217 | 0.768 |
| **IC16** | Global disability vs Motor disability | SPMS vs PPMS | -0.345 | 0.224 | 0.768 |
| **IC17** | Global disability vs Motor disability | SPMS vs PPMS | -0.318 | 0.219 | 0.768 |
| **IC18** | Global disability vs Motor disability | SPMS vs PPMS | 0.055 | 0.214 | 0.931 |
| **IC19** | Global disability vs Motor disability | SPMS vs PPMS | -0.038 | 0.219 | 0.931 |
| **IC20** | Global disability vs Motor disability | SPMS vs PPMS | -0.042 | 0.224 | 0.931 |
| **IC1** | Cognitive disability vs Motor disability | SPMS vs PPMS | -0.007 | 0.227 | 0.994 |
| **IC2** | Cognitive disability vs Motor disability | SPMS vs PPMS | -0.002 | 0.233 | 0.994 |
| **IC3** | Cognitive disability vs Motor disability | SPMS vs PPMS | -0.181 | 0.237 | 0.931 |
| **IC4** | Cognitive disability vs Motor disability | SPMS vs PPMS | 0.114 | 0.232 | 0.931 |
| **IC5** | Cognitive disability vs Motor disability | SPMS vs PPMS | -0.066 | 0.224 | 0.931 |
| **IC6** | Cognitive disability vs Motor disability | SPMS vs PPMS | -0.120 | 0.230 | 0.931 |
| **IC7** | Cognitive disability vs Motor disability | SPMS vs PPMS | -0.069 | 0.238 | 0.931 |
| **IC8** | Cognitive disability vs Motor disability | SPMS vs PPMS | 0.582 | 0.233 | 0.506 |
| **IC9** | Cognitive disability vs Motor disability | SPMS vs PPMS | 0.270 | 0.225 | 0.898 |
| **IC10** | Cognitive disability vs Motor disability | SPMS vs PPMS | -0.027 | 0.231 | 0.954 |
| **IC11** | Cognitive disability vs Motor disability | SPMS vs PPMS | 0.391 | 0.228 | 0.768 |
| **IC12** | Cognitive disability vs Motor disability | SPMS vs PPMS | -0.167 | 0.231 | 0.931 |
| **IC13** | Cognitive disability vs Motor disability | SPMS vs PPMS | 0.178 | 0.220 | 0.931 |
| **IC14** | Cognitive disability vs Motor disability | SPMS vs PPMS | -0.058 | 0.238 | 0.931 |
| **IC15** | Cognitive disability vs Motor disability | SPMS vs PPMS | 0.189 | 0.230 | 0.931 |
| **IC16** | Cognitive disability vs Motor disability | SPMS vs PPMS | -0.339 | 0.238 | 0.768 |
| **IC17** | Cognitive disability vs Motor disability | SPMS vs PPMS | -0.169 | 0.232 | 0.931 |
| **IC18** | Cognitive disability vs Motor disability | SPMS vs PPMS | 0.310 | 0.227 | 0.768 |
| **IC19** | Cognitive disability vs Motor disability | SPMS vs PPMS | -0.081 | 0.232 | 0.931 |
| **IC20** | Cognitive disability vs Motor disability | SPMS vs PPMS | -0.058 | 0.237 | 0.931 |

**Abbreviations**: ICC = independent component, SE = standard error, PPMS = Primary Progressive Multiple Sclerosis; SPMS = Secondary Progressive Multiple Sclerosis, FDR=False Discovery Rate.

**Sensitivity analysis: feature selection**

**Methods**

To identify the most relevant neuroanatomical predictors of distinct multiple sclerosis (MS) disability phenotypes, we applied Least Absolute Shrinkage and Selection Operator (LASSO) regression within a One-vs-All (OVA) classification framework, incorporating a bootstrapping strategy to enhance feature stability and reproducibility. This approach was chosen due to its ability to perform both variable selection and regularization, improving model interpretability while mitigating multicollinearity issues.^2, 3^

The LASSO model was implemented using the glmnet package^4^ with a fixed elastic-net mixing parameter (α = 0.5) to balance L1 (LASSO) and L2 (ridge regression) penalties, ensuring feature sparsity while preserving stability. A fixed λ = 0.1 was used to control the degree of regularization, preventing overfitting while retaining the most informative predictors.

**Results**

Features selected for each disability profiles are summarized below in **Supplementary Figure 7.**

**Supplementary Figure 7.** Summarizes selected features by using least absolute shrinkage and selection operator (LASSO) for each disability profile.

Supplementary Figure 7 displays the non-zero coefficients from the least absolute shrinkage and selection operator (LASSO) models fitted within a one-vs-all (OVA) classification framework for the motor (Panel A), cognitive (Panel B), and global (Panel C) disability profiles. Horizontal bars denote the standardised regression coefficients for each selected predictor: positive coefficients indicate that higher feature values are associated with the target profile, whereas negative coefficients indicate an inverse association.

**Abbreviations:** IC=independent component; NBV=normalized brain volume; NGMV=normalized grey matter volume.

**References**

1. Austin PC. Optimal caliper widths for propensity-score matching when estimating differences in means and differences in proportions in observational studies. Pharm Stat 2011;10:150-161.

2. Tibshirani R. Regression Shrinkage and Selection via the Lasso. Journal of the Royal Statistical Society Series B (Methodological) 1996;58:267-288.

3. Zou H, Hastie T. Regularization and Variable Selection Via the Elastic Net. Journal of the Royal Statistical Society Series B: Statistical Methodology 2005;67:301-320.

4. Friedman JH, Hastie T, Tibshirani R. Regularization Paths for Generalized Linear Models via Coordinate Descent. Journal of Statistical Software 2010;33:1 - 22.
