# Supplementary material for: Disability profiles in progressive multiple sclerosis reflect pathology distribution, independent of clinical phenotype
Source: Brain Commun. 2026 May 2;8(3):fcag162. doi: 10.1093/braincomms/fcag162 (PMC13201091; doi:10.1093/braincomms/fcag162)
Supplement: fcag162_Supplementary_Data [file fcag162_supplementary_data.zip › Supplementary_Video_1_legend.docx]

**Supplementary Video 1.** Examples of registration of T1-weighted images and lesion masks to the ICBM152 2009a space.
